# Nonlinear characteristics of gait signals in neurodegenerative diseases

**DOI:** 10.3389/fneur.2025.1607273

**Published:** 2025-06-16

**Authors:** Yang Yue, Na Chang, Zonglin Shi

**Affiliations:** ^1^Department of Mechanical Engineering, University College London, London, United Kingdom; ^2^Department of Neurology, Huaihe Hospital, Henan University, Kaifeng, China

**Keywords:** gait signal, ratio of left to right sequences, median filtering, nonlinear characteristics, complexity

## Abstract

Based on the asymmetric characteristics of left and right movements in patients with neurodegenerative diseases and their inherent coupling relationships, as well as the inevitable internal connection between them according to the principles of mechanical kinematics, and a processing method for the ratio of gait signals to left and right limb data is proposed. Using gait time series data collected from left and right limbs via pressure-sensitive insoles, a comparison was conducted among patients with Parkinson's disease (PD), Amyotrophic Lateral Sclerosis (ALS), Huntington's disease (HD), and a healthy control group (Ctrl) in terms of the average, standard deviation, and coefficient of variation of the left and right sequences, as well as the ratios between them. It was discovered that there exists a close correlation between the ratios of left to right sequences and the actual standard deviation and coefficient of variation of these sequences. These ratios can be utilized for identifying the categories of PD, ALS, and HD patients. After using a median filter (*n* = 3) to filter four sets of stride ratio data (Ctr1, A1s, PD, and HD), it was found that the data before filtering generally showed significant fluctuations, with many peaks and valleys, indicating that the original data may contain a lot of noise or outliers. In contrast, the filtered data showed relatively smaller fluctuations and a smoother curve, indicating that the filtering process effectively reduced noise in the data and enhanced its stability. The raw data distribution for the left and right limbs of patients with PD, ALS, HD, and the Ctrl was relatively large, posing certain difficulties in analyzing the patients' diseases. The use of the ratio of left to right data effectively improves the discreteness of the data. The ranking of CO complexity features from highest to lowest is ALS, HD, PD, and Ctrl. The ranking of sample entropy features from largest to smallest is ALS, HD, PD, and Ctrl. The ranking of wavelet coefficient features from largest to smallest is ALS, PD, HD, and Ctrl.

## 1 Introduction

At present, the scale assessment technique is that doctors observe the patient's gait, balance ability and other information based on the items in the standard scale to determine whether there are any abnormalities, and thereby score the severity of different types of neurological diseases. UPDRS and MDS-UPDRS are widely used in the condition monitoring, treatment effect evaluation and clinical research of patients with Parkinson's disease (PD) (1). UHDRS is used to assess the severity of Huntington's disease (HD) in patients (2). ALSFRS-R is a standardized scale used to evaluate functional disorders in patients with amyotrophic lateral sclerosis (ALS) (3).

Although these scales are widely used in clinical practice, these methods have significant subjectivity, making it difficult to accurately capture subtle changes in patients. With low reliability, they can only provide qualitative analysis and struggle to achieve objective and accurate quantitative analysis. To overcome these limitations, the medical community has begun exploring more precise and reliable monitoring methods and data processing techniques in recent years. For example, gait analysis-based technologies are being developed for the assessment of neurodegenerative diseases. By analyzing the gait characteristics of patients and combining machine learning and deep learning algorithms, rapid quantitative evaluations of dyskinesia can be achieved.

Gait is the external manifestation of posture and body state during human walking, reflecting individual differences in health status and physical function. Therefore, gait-based monitoring and analysis technology has been widely applied in multiple fields, including sports science, identity recognition, posture recognition, and health monitoring (4). In clinical medicine, gait analysis is particularly important. It can not only be used for the assessment and diagnosis of gait disorders, but also help doctors comprehensively understand the patient's walking ability and health status. This enables the formulation of precise diagnostic and treatment plans for patients with gait abnormalities, as well as the effective evaluation of treatment outcomes and rehabilitation progress.

Currently, gait acquisition and analysis technologies for patients mainly include scale evaluation technology, visual technology, sensing technology, and gait analysis systems based on pressure detection. (1) The scale assessment technique is used by doctors to observe the patient's gait, balance ability, and other information based on the items in a standard scale, and determine whether there are abnormalities, thereby assigning a fixed score to diagnose the severity of PD. The commonly used gait analysis scales include the Unified PD Rating Scale (UPDRS 3.0) (5), Hoehn Yahr Scale (6), and MDS Unified PD Rating Scale (MDS-UPDRS) (7). However, the assessment results of the scale depend on the clinical experience of the doctor, leading to a degree of subjectivity, and they struggle to capture detailed and subtle changes in gait abnormality. (2) Visual technology mainly utilizes optical motion capture systems, which involve placing multiple high-precision cameras within a fixed area to capture the spatial position changes of reflective markers worn on key parts of the human body or directly extract contour changes during human movement (8). This technology can provide relatively accurate three-dimensional motion data. However, its disadvantages include limited monitoring range and significant influence from factors such as light, shadow, and subject clothing (9). (3) Sensing technology collects gait information through sensors in contact with the human body, which can avoid interference from most environmental factors. Among them, wearable sensing technology integrates micro or flexible sensors into clothing or attaches them to the human body surface, collects gait information during walking without affecting comfort (10, 11). The collected data is then transmitted to a host computer via wireless transmission. This technology has the advantages of a wide monitoring range and being unaffected by external conditions. (4) The gait analysis system based on pressure detection reflects the interaction force between the sole and the ground during walking. With the widespread adoption of computer and pressure sensor technology, the measurement of sole pressure signals has become an important means of gait analysis. Common plantar pressure measuring devices include force measuring platforms, force measuring plates, and force measuring insole systems. For example, Shirakawa et al. (12) analyzed gait changes caused by aging using support vector machines by extracting temporal, spatial, dynamic, and kinematic features of gait. Kim et al. (13) analyzed the changes in gait of dementia patients based on plantar pressure and found that their walking speed and gait stability were significantly lower than those of healthy individuals. Mohamad et al. (14, 15) discussed the basic principles of piezoresistive pressure sensors and plantar measurement systems. Zhao et al. (16) evaluated the rehabilitation status of hemiplegic patients using a three-dimensional plantar pressure testing platform.

Although existing gait acquisition and analysis techniques for patients have been widely applied in clinical and research settings, each technique has its own advantages and disadvantages. Rating scale techniques rely heavily on the experience of doctors and are highly subjective. Visual techniques are greatly influenced by environmental factors. Sensor technologies and systems based on pressure detection offer advantages in objectivity and accuracy, but there is still room for improvement. In the future, a comprehensive gait analysis system combining multiple technological means is expected to overcome the limitations of a single technology, providing more comprehensive and accurate support for early diagnosis and rehabilitation evaluation of diseases.

With the advancement of computer and sensor technology, gait analysis systems based on pressure detection have been widely used in disease research in fields such as orthopedics, neurology, sports rehabilitation, and other fields. Gait information, as an important kinematic indicator, can be used for the diagnosis and evaluation of various diseases. As early as the 1960s, Murry established a comparative motion pattern between normal individuals and patients by splitting gait into multiple components, discovering that the gait of healthy people exhibits regular periodic signals, whereas the gait of patients shows irregular periodic signals (17, 18). Gait dynamics is regulated by a complex nervous system, and its feedback loop needs to span multiple spatiotemporal scales to adapt to environmental changes. Therefore, real-time analysis of gait parameters is of great significance for understanding the mechanisms of motion disorders. In recent years, researchers have conducted in-depth studies on patients with neurodegenerative diseases (NDDs) using gait analysis techniques. For example, Hausdorff et al. (19, 20) found that patients with amyotrophic lateral sclerosis (ALS) have longer strides, while HD patients have more random fluctuations in stride intervals. Vikram (21, 22) found that gait symmetry was significantly impaired in NDDs patients. Nakagawa (23–25) pointed out that patients with PD have significantly reduced walking speed, stride, and range of motion. These studies demonstrate that there are notable differences in gait dynamics between patients with NDDs and healthy individuals. In terms of gait complexity analysis, Aziz and Schache et al. (26–29) found that gait complexity was significantly reduced in ALS patients. Umar et al. (30–32) classified the gait rhythm of ALS patients and healthy individuals using statistical analysis methods, with an accuracy rate of 82.8%. By using the Swinging Interval Rotation Count (SWITC) parameter, gait pattern differentiation between ALS patients and healthy subjects can be achieved with a resolution of 89.66% (33). It can be seen that the gait analysis system based on pressure detection provides an important tool for the diagnosis and rehabilitation treatment of NDDs. With the advancement of technology, it has shown broad application prospects in the clinical field.

At present, the application of gait signals for neurodegenerative diseases mainly relies on direct analysis of collected data, which leads to the disconnection of internal connections between various collected data groups and affects the diagnosis of the disease. This article is based on the asymmetric characteristics of left and right movements in patients with neurodegenerative diseases and their inherent coupling relationship. Nonlinear dynamics theory is used to study the intrinsic relationship and application of gait signals in neurodegenerative diseases. The complexity, entropy, wavelet coefficients and other features of gait signals in the neurodegenerative disease database are extracted from the time series data of left and right limb gait collected by pressure-sensitive insoles. The original data and data considering left and right limb coupling are compared to explore the differences in feature distribution between neurodegenerative disease patients and Ctrl, providing a theoretical basis for the classification research of these diseases.

## 2 Source and processing methods of gait data

### 2.1 Data source of gait signals

This study is based on gait analysis using a neurodegenerative diseases (NDDs) database provided by Harvard Medical School in the United States, which is one of the most widely used databases in the field of movement information. This database can be accessed at (http://www.physionet.org/physiobank/database/gaitndd) (34). This database contains a total of 64 gait analysis data, covering the following population groups: 13 patients with amyotrophic lateral sclerosis (ALS), 15 patients with PD, 20 patients with Huntington's disease (HD), and 16 healthy control group (Ctrl) subjects. During the data review process, it was found that there were abnormalities in the records of the 20th HD patient, specifically the presence of negative values in the collected time series. Therefore, the patient's data was excluded from this study.The four groups of subjects were controlled and screened in terms of independent walking for 5 min, height, weight, etc., to ensure the reliability of the gait data of the subjects (35). Given the high prevalence of cognitive impairment in Parkinson's disease (PD), Huntington's disease (HD), and amyotrophic lateral sclerosis (ALS) populations–along with its well-documented independent effects on gait control–cognitive impairment represents a significant potential confounding factor in analyses. To address this limitation in future research, we strongly recommend using standardized cognitive assessment tools (e.g., the Montreal Cognitive Assessment [MoCA] or the University of Minnesota Battery [UMN-Battery]) to enable cognition-stratified analyses. Incorporating such assessments would also facilitate a deeper understanding of the interplay between cognitive function and gait disturbances in these neurodegenerative diseases.

The collection method of the database is as follows: the subjects walked continuously at their daily pace for 5 min on a 77 m long corridor. The gait signal is measured through pressure sensitive insoles placed inside the subjects' shoes, which record the ground reaction force acting on the feet in the vertical direction and are sampled at a frequency of 300Hz. After 12 bit analog-to-digital conversion, the collected signal is used to calculate the step measurement of foot contact time. Finally, gait analysis yielded the following 13 time series pairs: (1) Elapsed time (in seconds); (2) Left foot stride duration (seconds); (3) Duration of right foot stride (seconds); (4) Left foot swing duration (seconds); (5) Duration of right foot swing (seconds); (6) Duration of left foot swing (percentage of stride); (7) Duration of right foot swing (percentage of stride); (8) Duration of standing with left foot (seconds); (9) Duration of standing on the right foot (seconds); (10) Duration of standing with left foot (percentage of stride); (11) Duration of standing on the right foot (percentage of stride); (12) Duration of foot support (seconds); (13) Duration of foot support (percentage of stride).

The specific information for patients with different types of Neurodegenerative Diseases (NDDs) in the gait database is shown in Table 1, where the indicators vary among patients with different NDDs. For ALS patients, the duration since onset is used to indicate the severity of the disease. For PD patients, the Hoehn-Yahr scale is employed, with higher scores indicating more severe conditions. For HD patients, the composite capacity score is used, with lower scores implying a more advanced stage of the disease.

**Table 1:** Information on different populations in the neurodegenerative database (mean standard error).

**Category**	**Ctrl**	**ALS**	**PD**	**HD**
Number of people (individual)	16	13	15	20
Male (individual)	2	10	10	6
Female (individual)	14	3	5	14
Average age (year)	39.31 ± 4.62	55.61 ± 3.56	66.80 ± 2.80	46.65 ± 2.81
Average weight (kg)	66.80 ± 2.76	77.10 ± 6.10	75.07 ± 4.36	72.05 ± 3.81
Average height (m)	1.83 ± 0.02	1.74 ± 0.026	1.87 ± 0.04	1.83 ± 0.02
Average walking speed (m/s)	1.35 ± 0.04	1.05 ± 0.066	0.999 ± 0.052	1.15 ± 0.080
Average severity	0	18.30 ± 4.94	2.80 ± 0.22	6.90 ± 0.85

The data adopted by the research group was from the neurodegenerative Disease (NDDs) database provided by Harvard Medical School in the United States. Hausdorff et al. collected the data from this database based on the standards for obtaining medical data and controlled the four groups of subjects in terms of independent walking for 5 min, height, weight, etc.According to the paper published by Hausdorff et al. (35), we obtained the relevant data as shown in the following Table 2. Kruskal-Wallis tests detected significant differences among the 4 groups for all measures. Compared with the healthy control group, there were significant differences in the gait speed and fluctuation amplitude indicators between HD and PD patients, but the gait time was similar in these groups. Compared with ALS patients, there are also significant differences in the fluctuation dynamic indicators of HD patients. Gait speed and gait time are similar in HD and PD patients, but all fluctuation amplitudes and dynamic indicators are significantly different in these two types of patients. The Kruskal-Wallis test showed that among all the measured indicators, there were significant differences among the four groups. These differences may be caused by factors such as age, gender, height, weight and walking speed, and these differences will bring certain difficulties to accurately distinguish neurological diseases. To reduce the influence of these differences on the differentiation of neurological diseases, the research group, based on the principles of mechanical kinematics and the asymmetry of human body's own motor symptoms, adopted the data ratio of the left and right limbs of the body to re-obtain the method of differentiating neurological disease data, and reduced the influence of factors such as age, gender, height and weight on the results.

**Table 2:** Gait rhythm dynamics.

**Category**	**Ctrl**	**Als**	**PD**	**HD**
**Average values**				
Stride time, ms	1.091 ± 23^*###*^	1.370 ± 61	1.118 ± 30^**^	1.138 ± 38^***^
Speed, m/s	1.35 ± 0.04^#^	1.02 ± 0.07	1.00 ± 0.05	1.15 ± 0.08
**Fluctuation magnitude**				
Stride time CV, (%)	2.3 ± 0.1^**^	4.5 ± 0.6	4.4 ± 0.6	7.6 ± 1.2
Stride time SD-dt, ms	27 ± 2^#^	65 ± 10	52 ± 6	120 ± 25
**Fluctuation dynamics**				
α	0.91 ± 0.05	0.74 ± 0.07	0.82 ± 0.06	0.60 ± 0.04
Autocorrelation decay time	5.9 ± 0.4^*^	4.2 ± 0.6	7.2 ± 1.6	3.2 ± 0.5
Nonstationarity index	0.647 ± 0.02	0.69 ± 0.05	0.64 ± 0.03	0.54 ± 0.03^*^

Values are means ± SE. CV, coefficient of variation; α, fractal scaling index; SD-dt, SD of the detrended time series. *P < 0.05, **P < 0.01, ^#^*P* < 0.001, ^*##*^*P* < 0.0001 compared with subjects with ALS.

### 2.2 Processing method of gait signals

To achieve efficient intelligent classification and detection of neurodegenerative diseases, a processing method for gait signal data is proposed. Firstly, the gait time series is extracted from the bipedal force data measured by pressure-sensitive insoles, and the first 11 columns of data are selected for the study of human gait feature parameters. Subsequently, these time series signals are subjected to noise processing to remove interference and preserve key information. On this basis, the nonlinear dynamic characteristics embedded within the gait signals are deeply explored. These characteristics can effectively reflect the complexity and inherent laws of neurodegenerative diseases.

### 2.3 Conversion method of gait signals

The motor symptoms of neurological diseases such as PD often first appear on one side of the body and gradually affect the other side. Therefore, the left and right limb movements of neurological diseases such as PD exhibits the following characteristics: (1) Asymmetry of motor symptoms: The motor symptoms of PD include resting tremor, muscle rigidity, bradykinesia, and abnormal posture and gait, among others. These symptoms may initially present asymmetry. This means that patients may first experience these symptoms on one side of their body and then gradually on the other side. (2) Asymmetry of tremor: Tremors usually start from the distal end of one upper limb, mainly from the thumb, index finger, and middle finger, and may then extend to the ipsilateral lower limb and contralateral limb. Tremors in the upper limbs are usually more severe than those in the lower limbs. (3) Asymmetry of muscle stiffness: Muscle stiffness is characterized by increased muscle tone in both active and antagonistic muscle groups, and uniform resistance can be felt during passive joint movement. This stiffness may first appear in one limb and gradually affect the other. (4) Asymmetry of bradykinesia: Due to muscle stiffness, patients are unable to make continuous movements, resulting in inflexibility and reduced or even absent swing amplitude of the affected upper limb. (5) Asymmetry of abnormal posture and gait: PD patients may exhibit inflexibility in turning and turning, and as the condition progresses, they are prone to falls. These symptoms usually first affect one side and then gradually spread to the other side. It can be seen that neurological diseases such as PD have significant asymmetry in the movement of the left and right limbs, resulting in significant differences in gait data between the left and right limbs. This discrepancy introduces complexity and unreadability in the analysis of gait data, yet there is an inherent and inevitable connection between them based on the principles of mechanical kinematics. Therefore, based on the comparison of the mean, standard deviation, and coefficient of variation of the left and right sequences and their ratios between PD, ALS, HD and the healthy Ctrl in Table 3. It was found that there is a close correlation between the ratio of left and right sequences and the actual standard deviation and coefficient of variation of the left and right sequences. To enhance the efficient utilization of gait data, the research team derived the ratio of left to right step length intervals from the original data. Similarly, the ratio of left to right swing intervals, left to right swing intervals, left to right stance intervals, and left to right standing intervals were obtained. Therefore, these ratios of left-right sequences were adopted for the identification of PD, ALS, and HD categories.

**Table 3:** Statistical analysis information of gait data [mean ± SD (CV)].

**Gait data**	**ALS**	**PD**	**HD**	**Ctrl**
Left stride interval	1.3911 ± 0.2073	1.1421 ± 0.1106	1.1605 ± 0.1635	1.0932 ± 0.0887
Right stride interval	1.4251 ± 0.2748	1.1371 ± 0.1062	1.1616 ± 0.1679	1.0926 ± 0.0887
Ratio of left to right stride	1.0177 ± 0.0331	1.0008 ± 0.0031	1.0003 ± 0.0067	0.9969 ± 0.0015
Left waggle interval	0.4336 ± 0.0463	0.3690 ± 0.0536	0.3827 ± 0.0595	0.3892 ± 0.0396
Right waggle interval	0.4336 ± 0.0463	0.3690 ± 0.0536	0.3827 ± 0.0595	0.3892 ± 0.0396
Ratio of left to right waggle	1.0196 ± 0.0718	1.0412 ± 0.1147	1.0900 ± 0.1675	1.0170 ± 0.0636
Left swing interval	32.3393 ± 2.938	33.2843 ± 2.397	34.6533 ± 3.319	36.0294 ± 1.636
Right swing interval	32.0340 ± 2.938	32.6906 ± 3.644	33.2371 ± 3.894	35.4661 ± 1.771
Ratio of left to right swing	1.0386 ± 0.0915	1.0306 ± 0.1102	1.0705 ± 0.1615	1.0161 ± 0.0635
Left stance interval	0.9553 ± 0.1843	0.7677 ± 0.0955	0.7596 ± 0.1162	0.6979 ± 0.0576
Right stance interval	0.9914 ± 0.2475	0.7681 ± 0.0929	0.7789 ± 0.1316	0.7034 ± 0.056
Ratio of left to right stance	1.0242 ± 0.0559	0.9953 ± 0.0609	0.9839 ± 0.0606	0.9891 ± 0.0311
Left stance interval	67.7190 ± 3.057	66.72 ± 2.3971	65.3467 ± 3.319	63.576 ± 1.6412
Right stance interval	68.0243 ± 2.412	67.3094 ± 3.664	66.7628 ± 3.894	64.1390 ± 1.776
Ratio of left to right stance	0.9978 ± 0.0322	0.9969 ± 0.0639	0.9822 ± 0.0582	0.9883 ± 0.0305

### 2.4 Processing results of gait data

The experimental design for neurodegenerative diseases involves the collector walking back and forth for 5 min on a 77 m channel, resulting in multiple turns during the walking process. To eliminate the unstable data caused by the stationary start of the collector during the testing process, it is necessary to preprocess the entire gait signal before analyzing and extracting features from the gait data.

The theoretical basis for using median filtering method is to replace the value of a point with the median of each point in its neighborhood. Suppose there exists a one-dimensional time series x=x(1), x(2),...x(m) with a length of m. Assuming the window width of the median filter is n, in order to ensure the existence of the median, n must be an odd number. For the padded signal x=x(-k+1), x(-k+2),...x(-k+m), as shown in Equation 1.


(1) $$x=\{\begin{array}{ll} x(1) & \text{if }-k\leq n<1\\ x(n) & \text{if }1\leq n<m\\ x(m-1) & \text{if }m\leq n<m+1 \end{array}$$

After applying the median filter to a time series, a sliding window will be formed. The sequence of *n* = 2k + 1 point values in the window is x(i-k), x(i-k+1), x(i-k+2),...x(i-k). Sort them from small to large to obtain a new sequence F(i-k), F(i-k+1), F(i-k+2),...F(i-k). The middle point F(i) is the value obtained after median filtering. Using X(i) to represent the value after median filtering, the time series after median filtering for the original time series x = x(1), x(2),...x(m) is X=X(1), X(2),...X(m). According to research, the filtering effect is best when *n* = 3. The Ctor1 stride ratio fluctuates between 0.95 and 1.05 at most time points. After filtering, the A1s stride ratio shows a significant upward trend toward 250. Similarly, the PD step length ratios also exhibit a clear upward trend near the 250-time point after filtering. The HD step length ratios display notable upward trends at times close to both 100 and 200 after filtering.

**Figure 1:**
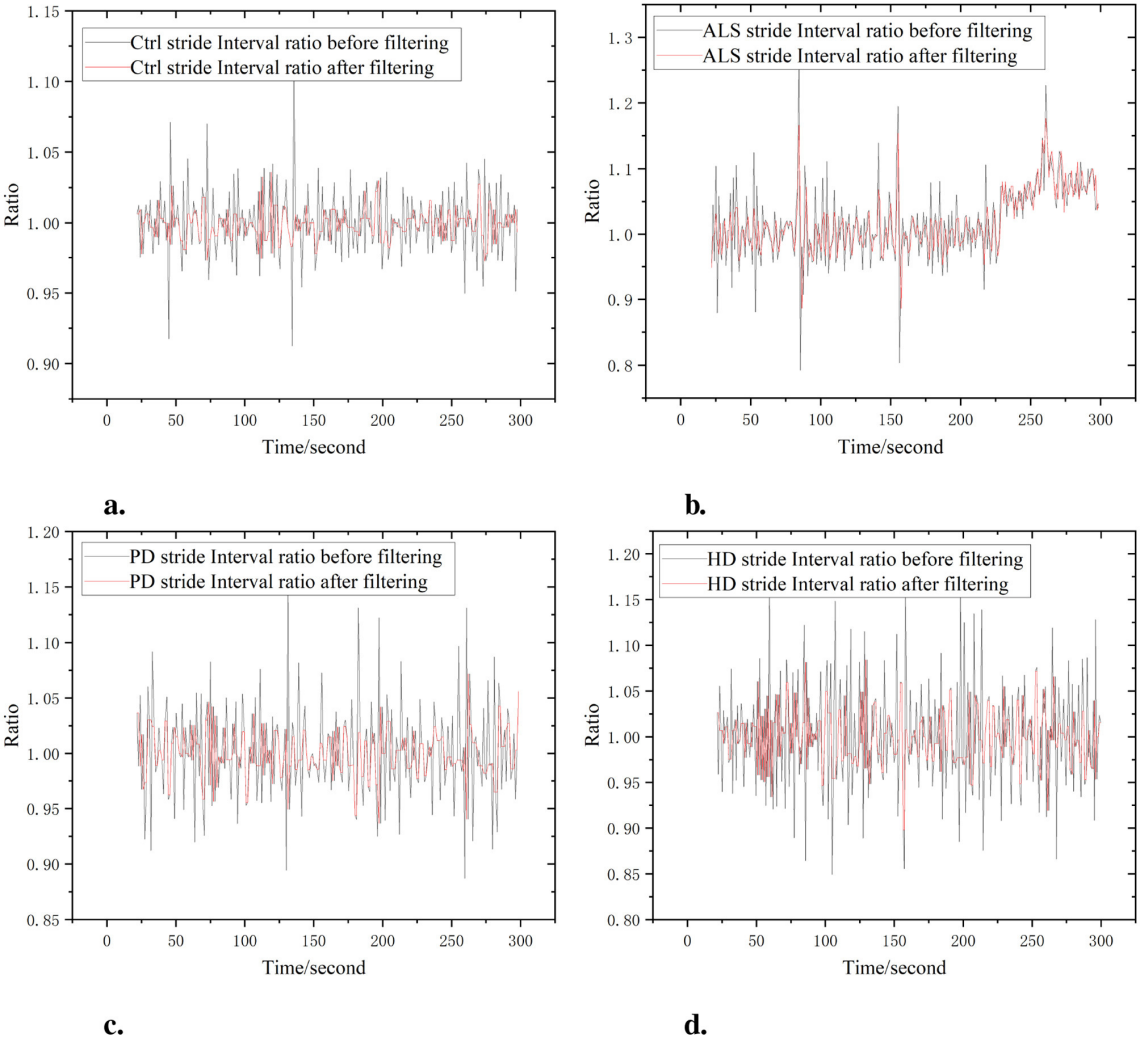
Comparison of left and right limb data before and after filtering. **(a)** Ctrl. **(b)** ALS. **(c)** PD. **(d)** HD.

As shown in Figure 1, after the filtering effect of the four sets of stride ratio data (Ctr1, ALS, PD, HD), it can be found that the data before filtering (black lines) generally show large fluctuations, with many peaks and valleys, indicating that the original data may contain a lot of noise or outliers. The filtered data (red lines) has relatively small fluctuations and a smoother curve, indicating that the filtering process effectively reduces noise in the data and makes it more stable. The overall trends of the data sequences before and after filtering are similar, indicating that the filtering process has not significantly altered the basic trends of the data, only removing some fluctuations. At certain time points, the filtered data shows more obvious trend changes. The ratio range of the filtered data is slightly reduced, further indicating that the filtering process reduces the volatility of the data. The data before filtering showed significant outliers at certain time points, which are well suppressed after filtering.

## 3 Gait feature extraction based on nonlinear dynamics

The human body is a complex integration of physiological systems at various levels, and the physiological signals generated are highly complex, especially in time series. Gait data collection belongs to limited data samples, and it is very appropriate to use complexity and entropy to reveal the nonlinear dynamic characteristics of gait systems in neurodegenerative diseases. Meanwhile, wavelet coefficients are utilized in the time- frequency domain to explore gait information. By comparing the duration of left foot stride, right foot stride, left foot swing, right foot swing, left foot swing (percentage of stride), right foot swing (percentage of stride), left foot standing, right foot standing, left foot standing (percentage of stride), and right foot standing (percentage of stride) with their corresponding ratios of left to right stride intervals, left to right swing intervals, left to right swing intervals, left to right stance intervals, and left to right stance intervals. A new method for gait signal data processing and accurate application is proposed.

### 3.1 C0 complexity

Complexity is an indicator used to quantitatively describe the degree of signal complexity, with its numerical value reflecting the degree of disorder in the signal. In gait signal analysis, complexity can effectively describe the degree of disorder in gait time series and indirectly reflect the activity of human gait. For example, the complexity of gait signals generated by the human body during acceleration walking is significantly higher than that during uniform walking, indicating that complexity can sensitively capture the characteristics of gait dynamic changes. C0 complexity is an improved method based on LZ complexity. The traditional LZ complexity may result in information loss during the calculation process due to excessive coarsening, thereby masking the inherent features of the system. In contrast, the core idea of C0 complexity is to separate the regular and irregular parts of the signal. This method does not have strict requirements for data length, and can obtain relatively reliable results even in short data, while avoiding computational errors introduced by coarse-grained processing. The calculation formula for C0 complexity is shown in Equation 2.


(2) $$\begin{array}{l} CO=\frac{\sum_{t=0}^{N-1}|x\left(t\right)-\tilde{x}\left(t\right){|}^{2}}{\sum_{t=0}^{N-1}|x\left(t\right){|}^{2}}. \end{array}$$

Based on the distribution of CO complexity characteristics provided in Table 4, a detailed analysis of the gait data for the ALS, Park, Hunt, and Ctrl groups is conducted. In terms of the left stride interval, the ALS group has a CO complexity of 0.0190 ± 0.0333, showing significant variability, indicating a larger difference in stride intervals and irregular gait. This study found that ALS patients exhibited significantly increased gait variability, a phenomenon that may be associated with multi-system dysfunction. Dubbioso et al. demonstrated through rigorous dual-task paradigms that ALS patients with mild cognitive impairment (MCI) showed significantly greater gait variability compared to cognitively intact patients, particularly under task conditions requiring executive function (33, 36). The study specifically noted that this increased variability was independent of the degree of motor dysfunction, suggesting that cognitive factors may influence gait control through the prefrontal-basal ganglia circuit.The Park group has a CO complexity of 0.0087 ± 0.0109, with gait being more stable but still showing irregularity compared to other groups; the Hunt group has a CO complexity of 0.0282 ± 0.0481, with stride interval differences slightly less than the Park group, but still showing some irregularity; the Ctrl group has the lowest CO complexity of 0.0019 ± 0.0013 among the four groups, indicating more consistent and symmetric gait, with stable stride intervals.

**Table 4:** CO complexity characteristics (mean ± standard deviation).

**Gait data**	**ALS**	**PD**	**HD**	**Ctrl**
Left stride interval	0.0190 ± 0.0333	0.0087 ± 0.0109	0.0282 ± 0.0481	0.0019 ± 0.0013
Right stride interval	0.0978 ± 0.2288	0.0089 ± 0.0086	0.0274 ± 0.0494	0.0017 ± 0.0010
Ratio of left to right stride	0.0057 ± 0.0116	0.0019 ± 0.0031	0.0046 ± 0.0077	0.00013 ± 0.0001
Left waggle interval	0.0089 ± 0.0069	0.0132 ± 0.0108	0.0369 ± 0.00457	0.0024 ± 0.0012
Right waggle interval	0.0087 ± 0.0075	0.0183 ± 0.0234	0.0352 ± 0.0355	0.0027 ± 0.0012
Ratio of left to right waggle	0.0099 ± 0.0079	0.0213 ± 0.0130	0.0322 ± 0.0388	0.0015 ± 0.0011
Left swing interval	0.0085 ± 0.0068	0.0085 ± 0.0059	0.0195 ± 0.0244	0.0016 ± 0.0010
Right swing interval	0.0079 ± 0.0063	0.0090 ± 0.0055	0.0237 ± 0.0242	0.0016 ± 0.0009
Ratio of left to right swing	0.1510 ± 0.0219	0.0083 ± 0.0057	0.0236 ± 0.0246	0.0014 ± 0.0011
Left stance interval	0.0270 ± 0.0606	0.0136 ± 0.0201	0.0427 ± 0.0923	0.0033 ± 0.0026
Right stance interval	0.0591 ± 0.0967	0.0132 ± 0.0135	0.0472 ± 0.0923	0.0028 ± 0.0018
Ratio of left to right stance	0.0083 ± 0.0177	0.0053 ± 0.0060	0.0086 ± 0.0128	0.0005 ± 0.0003
Left stance interval	0.0019 ± 0.0016	0.0019 ± 0.0011	0.0048 ± 0.0057	0.0005 ± 0.0003
Right stance interval	0.0018 ± 0.0015	0.0021 ± 0.0016	0.0052 ± 0.0057	0.0005 ± 0.0004
Ratio of left to right stance	0.0015 ± 0.0010	0.0026 ± 0.0051	0.0046 ± 0.0054	0.0005 ± 0.0007

In terms of the right stride interval, the ALS group has a sample entropy of 0.0978 ± 0.2288, similar to the left stride interval, indicating poor consistency in stride intervals and asymmetric gait; the PD group has a sample entropy of 0.0089 ± 0.0086, showing more symmetric gait; the HD group has a sample entropy of 0.0274 ± 0.0494, demonstrating stronger symmetry; the Ctrl group has a sample entropy of 0.0017 ± 0.0010, confirming good gait control in the Ctrl. Regarding the stride ratio, the ALS group has a sample entropy of 0.0057 ± 0.0116, indicating asymmetric gait with larger differences in stride intervals; the PD group has a sample entropy of 0.0019 ± 0.0031, slightly lower than the ALS group, showing better gait symmetry; the HD group has a sample entropy of 0.0046 ± 0.0077, similar to the PD group, showing good gait symmetry; the Ctrl group has a sample entropy of 0.00013 ± 0.0001, showing the least complexity and the most symmetric gait. In terms of the left waggle interval, the ALS group has a sample entropy of 0.0089 ± 0.0069, showing some variability but not as significant as the stride intervals; the PD group has a sample entropy of 0.0132 ± 0.0108, with gait being more stable but still showing irregularity; the HD group has a sample entropy of 0.0369 ± 0.0047, with stride interval differences slightly less than the PD group, but still showing some irregularity; the Ctrl group has a sample entropy of 0.0024 ± 0.0012, indicating more consistent and symmetric gait, with stable stride intervals.

Regarding the waggle ratio, the ALS group has a sample entropy of 0.0099 ± 0.0079, indicating asymmetric gait with larger differences in stride intervals; the PD group has a sample entropy of 0.0213 ± 0.0130, slightly lower than the ALS group, showing better gait symmetry; the HD group has a sample entropy of 0.0322 ± 0.0388, similar to the PD group, showing good gait symmetry; the Ctrl group has a sample entropy of 0.0015 ± 0.0011, showing the least complexity and the most symmetric gait. In terms of the left stance interval, the ALS group has a sample entropy of 0.0270 ± 0.0606, showing significant variability, indicating irregular gait; the PD group has a sample entropy of 0.0136 ± 0.0201, with gait being more stable but still showing irregularity compared to other groups; the HD group has a sample entropy of 0.0427 ± 0.0923, with stride interval differences slightly less than the PD group, but still showing some irregularity; the Ctrl group has a sample entropy of 0.0033 ± 0.0026, indicating more consistent and symmetric gait, with stable stride intervals. The Ctrl group shows the lowest sample entropy in all gait data, with the most consistent and symmetric gait. The ALS group shows higher sample entropy in most gait data, with irregular and highly asymmetric gait. The PD and HD groups have gait characteristics between the ALS and Ctrl groups, showing some irregularity and asymmetry.

**Figure 2:**
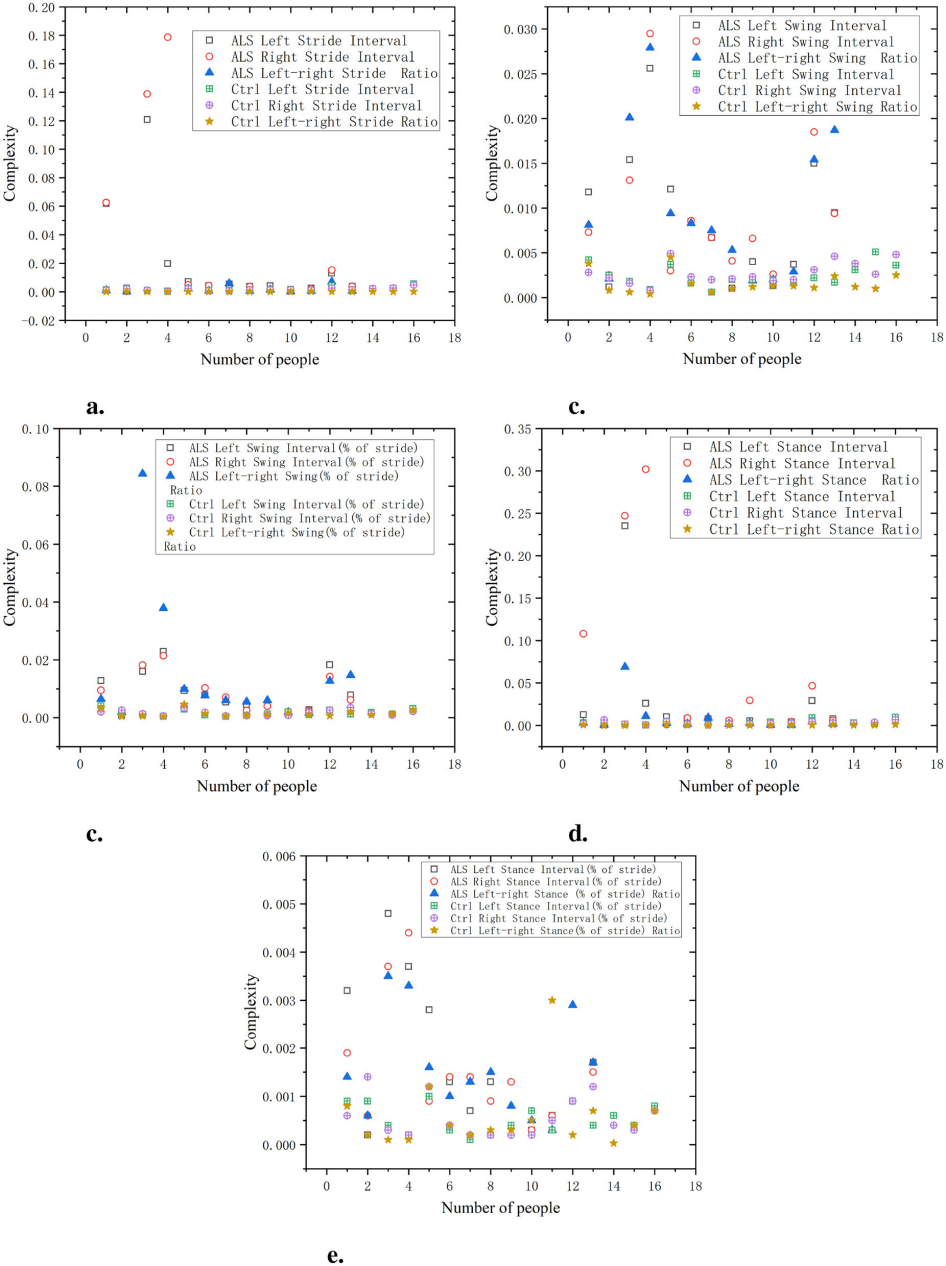
Comparison of C0 complexity characteristics between Ctrl and ALS patients. **(a)** Stride interval. **(b)** Swing interval. **(c)** Swing interval of stride. **(d)** Stance interval. **(e)** Stance interval of stride.

As shown in Figure 2, the left/right stride interval complexity of the ALS group is relatively high, with a complexity close to 0.14–0.18, indicating significant stride variations and irregular gait patterns. In contrast, the stride interval complexity of the Ctrl group always remains at a low level, close to 0.02 or below. The gait swing interval of the ALS group has a significant complexity, approaching 0.03. The complexity of gait swing intervals in the Ctrl group is relatively low, approaching 0.005, demonstrating a higher degree of gait regularity. The ALS group had higher complexity in the standardized gait swing interval, while the Ctrl group had consistently lower complexity, almost unchanged and maintained below 0.01. The gait support interval complexity of the ALS group was relatively high, approaching 0.35, while the complexity of the Ctrl group remained consistently low, exhibiting a highly consistent gait with complexity kept below 0.02. Additionally, the gait stance interval complexity in the ALS group can approach 0.006, while the complexity of the Ctrl group is consistently low, close to 0.001. The ratio of left and right signals has a lower complexity and is positively distributed with the original signal, suggesting that this ratio can reflect the disease characteristics of the original data in a relatively simple manner.

**Figure 3:**
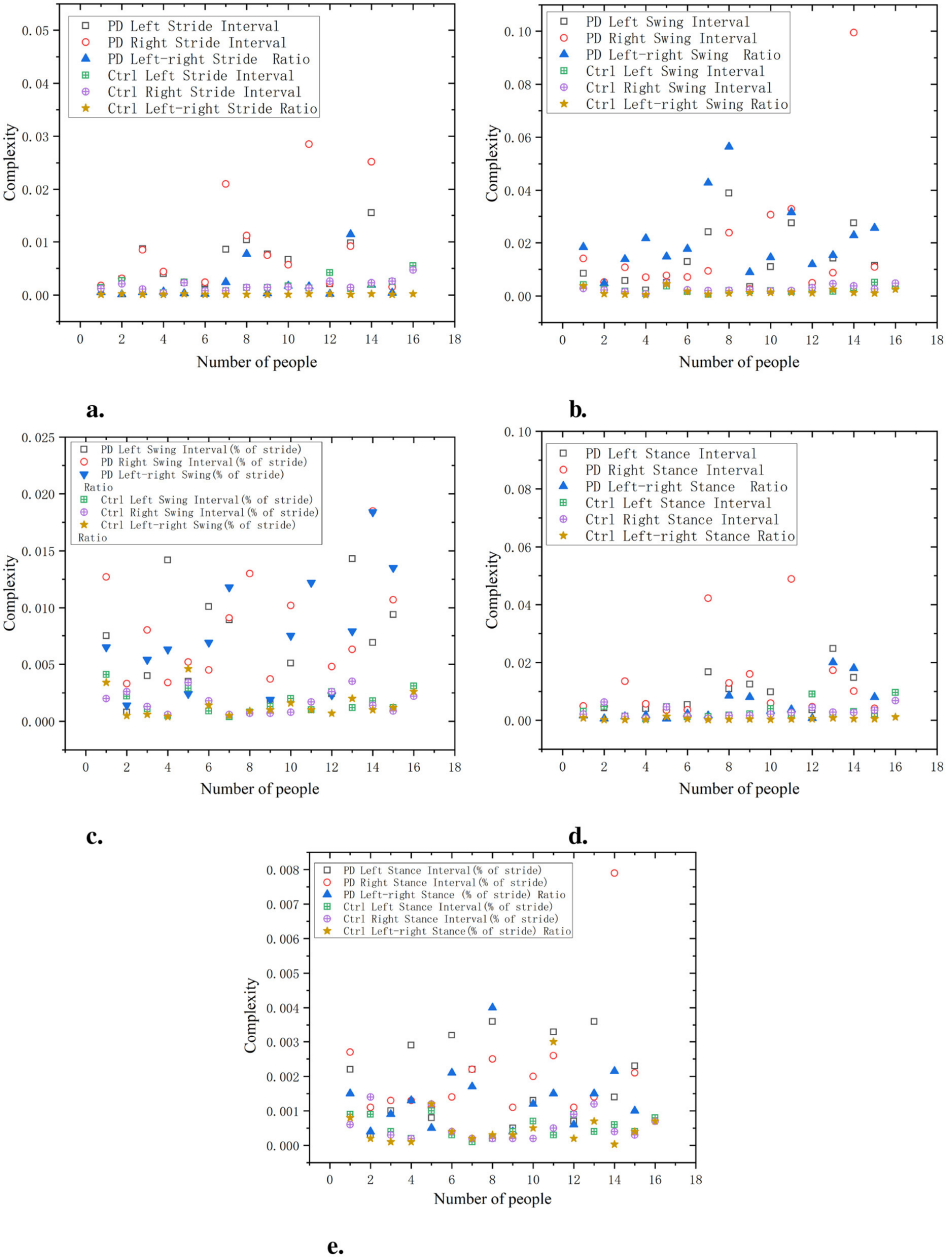
Comparison of C0 complexity characteristics between Ctrl and PD. **(a)** Stride interval. **(b)** Swing interval. **(c)** Swing interval of stride. **(d)** Stance interval. **(e)** Stance interval of stride.

As shown in Figure 3, in the PD group, the complexity of both the left stride interval and the right stride interval is relatively high, approaching values between 0.03 and 0.05. In the Ctrl group, the complexity of stride intervals is always low and remains below 0.01. The complexity of the Swing Ratio in the PD group is more pronounced, approaching 0.05, indicating a high degree of gait asymmetry. In contrast, the complexity of the Ctrl group has always been low, almost approaching 0.01. In terms of gait support interval, the PD group showed a high level of complexity, approaching 0.08. The complexity of the Ctrl group is relatively low, consistently maintained between 0.01 and 0.02, showing small gait differences and more regular gait control. In the standardized gait support interval, the complexity of the PD group is consistently high, ranging from approximately 0.005–0.008, with gait exhibiting higher asymmetry and irregularity. The complexity of the gait stance interval in the Ctrl group remains consistently low, close to 0.001–0.003, demonstrating high consistency and gait symmetry. The left to right stride ratio of the Ctrl group exhibits better regularity and less dispersion compared to the PD group.

**Figure 4:**
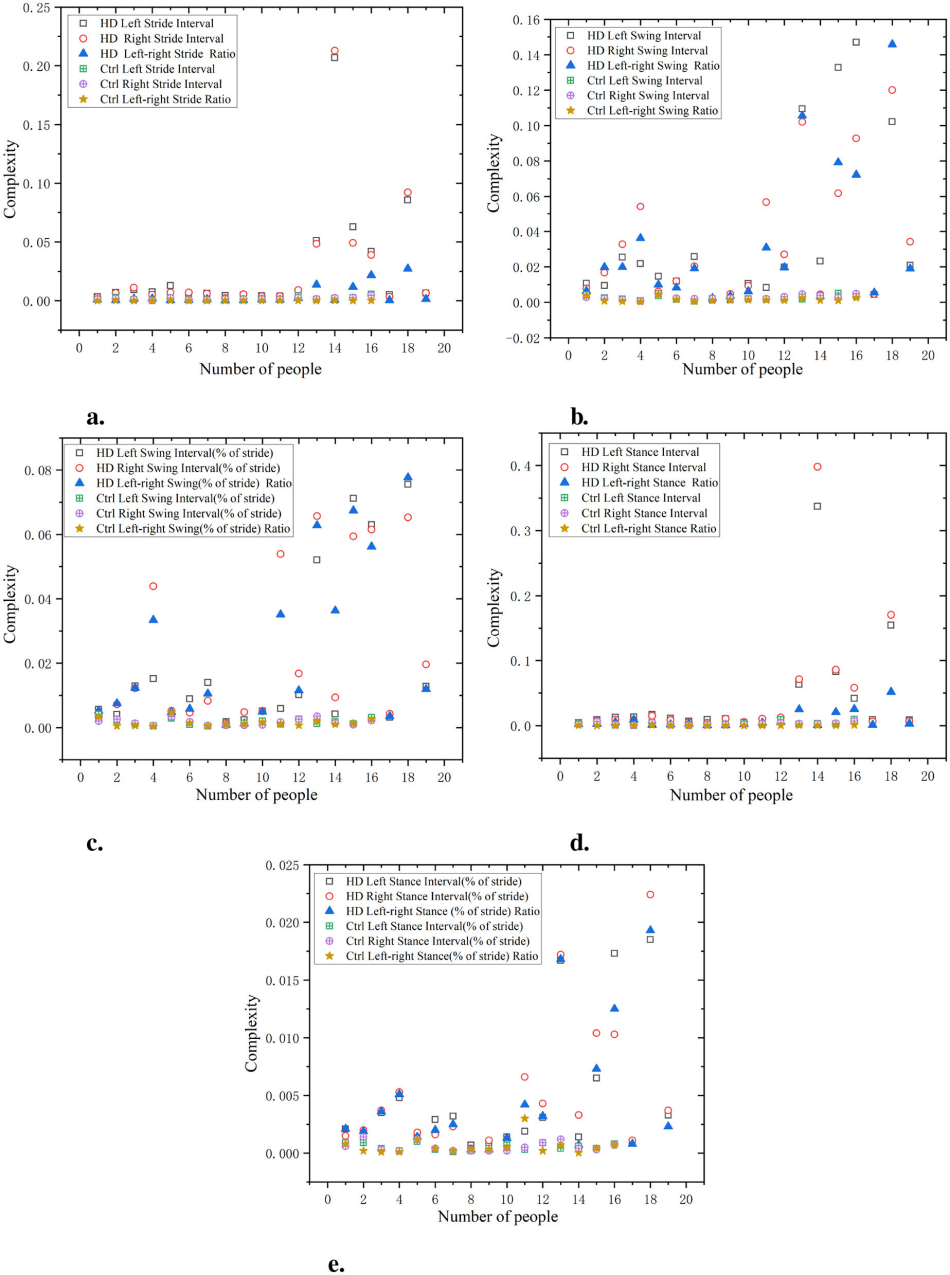
Comparison of C0 complexity characteristics between Ctrl and HD patients. **(a)** Stride interval. **(b)** Swing interval. **(c)** Swing interval of stride. **(d)** Stance interval. **(e)** Stance interval of stride.

As shown in Figure 4, the complexity of the HD left and right stride intervals is relatively high, particularly in the variations between the left and right strides, with a complexity approaching 0.20–0.25. This indicates significant stride variations and irregular gait patterns. In contrast, the stride interval complexity of the Ctrl group remained at around 0.01, indicating that the gait of this group was relatively consistent and the difference in left and right stride was small. The complexity of the HD left and right swing intervals is close to 0.16–0.18, while the complexity of the Ctrl left and right swing intervals consistently approaches 0.01. In normalized gait swing intervals, the HD group exhibits higher complexity, while the complexity in the Ctrl group remains close to 0.005. The complexity of the HD left and right stance intervals is also high, approaching 0.35. In comparison, the gait stance interval complexity in the Ctrl group is lower, consistently staying below 0.2. In normalized gait stance intervals, the HD group had higher complexity, while the Ctrl group had consistently lower gait support interval complexity, approaching 0.001, demonstrating a highly consistent gait pattern. The ratio of left and right signals has a lower complexity and is positively distributed with the original signal, indicating that this ratio can reflect the disease characteristics of the original data in a relatively simple manner.

### 3.2 Wavelet entropy

Wavelet entropy, grounded in the theory of wavelet transform, is an entropy value calculated by decomposing a signal sequence through wavelet analysis and obtained from wavelet coefficients. Because wavelet transform can reflect the time-frequency information contained in signals, wavelet entropy theory is based on wavelet theory analysis similar to information entropy theory. Therefore, the wavelet entropy generated on this basis can quantitatively describe the energy analysis of signals in the time-frequency do- main. A smaller wavelet entropy value indicates that the signal sequence is more ordered, representing less variation in the signal. The calculation formula is as follows:


(3) $$\begin{array}{l} S_{\text{WT}}=\underset{j}{\sum }P_{j}\log P_{j} \end{array}$$

Based on the calculation of wavelet entropy above, the distribution of characteristic values is shown in Table 5. In terms of left stride, the ALS group had a left stride width of 0.0678 ± 0.1063, indicating significant gait differences. The left stride width of the PD group is 0.0351 ± 0.0393, which is relatively low and shows little gait difference. The left stride width of the HD group was 0.1006 ± 0.1413, showing significant gait differences. The left stride width of the Ctrl group is 0.0097 ± 0.0055, indicating highly consistent gait.

**Table 5:** Wavelet entropy (mean ± standard deviation).

**Gait data**	**ALS**	**PD**	**HD**	**Ctrl**
Left stride interval	0.0678 ± 0.1063	0.0351 ± 0.0393	0.1006 ± 0.1413	0.0097 ± 0.0055
Right stride interval	0.1163 ± 0.1554	0.0570 ± 0.0289	0.1142 ± 0.1356	0.0368 ± 0.0060
Ratio of left to right stride	0.7517 ± 0.0215	0.7247 ± 0.0118	0.7264 ± 0.0203	0.7177 ± 0.0098
Left waggle interval	0.5393 ± 0.0421	0.5734 ± 0.0843	0.5730 ± 0.0723	0.5308 ± 0.0386
Right waggle interval	0.6784 ± 0.0446	0.7142 ± 0.0762	0.7338 ± 0.0660	0.6854 ± 0.0422
Ratio of left to right waggle	0.7866 ± 0.0454	0.7650 ± 0.0494	1.0681 ± 0.077	0.7482 ± 0.0323
Left swing interval	0.0798 ± 0.0327	0.0728 ± 0.0249	0.1125 ± 0.0844	0.0441 ± 0.0058
Right swing interval	0.0684 ± 0.0231	0.0710 ± 0.0196	0.1253 ± 0.0881	0.0417 ± 0.0054
Ratio of left to right swing	0.8172 ± 0.0370	0.7866 ± 0.0460	0.7689 ± 0.0538	0.7819 ± 0.0299
Left stance interval	0.6169 ± 0.1012	0.6531 ± 0.0553	0.6868 ± 0.0794	0.6760 ± 0.0323
Right stance interval	0.6959 ± 0.1002	0.7137 ± 0.0363	0.7320 ± 0.0865	0.7332 ± 0.0289
Ratio of left to right stance	0.8511 ± 0.0278	0.8236 ± 0.0274	0.8335 ± 0.0252	0.8208 ± 0.0123
Left stance interval	0.0522 ± 0.0114	0.0455 ± 0.0076	0.0555 ± 0.025	0.0372 ± 0.0028
Right stance interval	0.0497 ± 0.0121	0.0428 ± 0.0039	0.0609 ± 0.029	0.0365 ± 0.0025
Ratio of left to right stance	0.8663 ± 0.0192	0.8456 ± 0.0266	0.8561 ± 0.0234	0.8450 ± 0.0113

For right stride, the ALS group had a right stride width of 0.1163 ± 0.1554, with significant differences in gait. The width of the right stride in the PD group is 0.0570 ± 0.0289, with a smaller stride and a more consistent gait. The width of the right stride in the HD group is 0.1142 ± 0.1356, which is relatively large and has strong gait inconsistency. The width of the right stride in the Ctrl group is 0.0368 ± 0.0060, showing the most consistent stride.

In terms of the ratio of left to right stride intervals, the ALS group had a ratio of 0.7866 ± 0.0454, indicating a relatively asymmetric stride and significant differences. The PD group ratio is 0.7650 ± 0.0494, showing a small difference. The ratio of HD group is 1.0681 ± 0.077, which is relatively high, indicating significant differences in gait. The Ctrl group ratio is 0.7482 ± 0.0323, demonstrating high gait symmetry.

**Figure 5:**
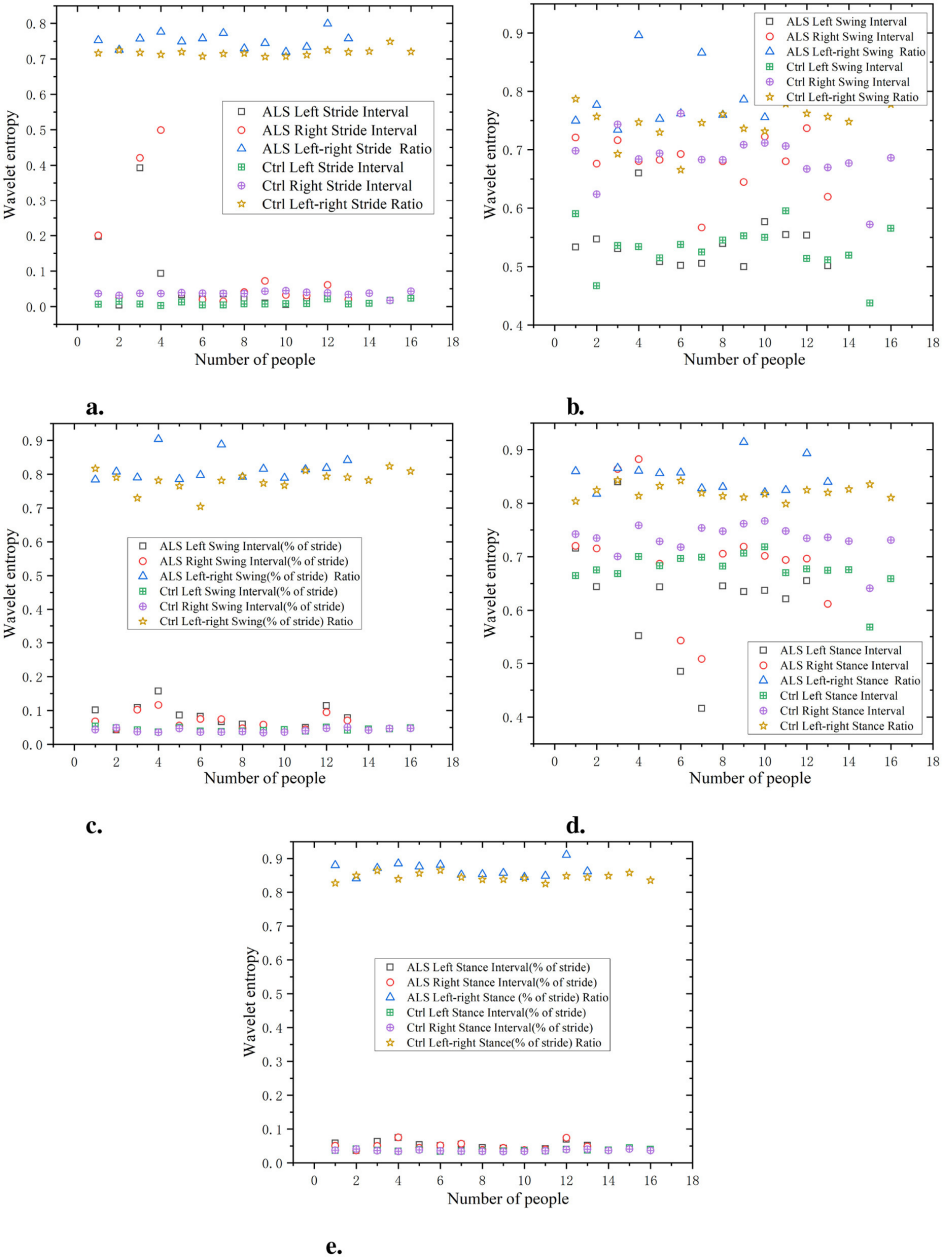
Comparison of wavelet entropy characteristics between Ctrl and ALS patients. **(a)** Stride interval. **(b)** Swing interval. **(c)** Swing interval of stride. **(d)** Stance interval. **(e)** Stance interval of stride.

As shown in Figure 5, the waveform entropy of the ALS group is usually higher, indicating greater fluctuations in the patient's stride. For example, the waveform entropy of a certain data point (such as ALS left stride interval) may be 0.7, while the waveform entropy of the Ctrl is 0.3, indicating that ALS patients have significant instability in controlling stride. The waveform entropy of swing intervals in the ALS group is also typically higher, revealing greater variability in the oscillation part of gait. For example, a data point may show a waveform entropy of 0.8 for the right swing interval in the ALS group, while the Ctrl has a waveform entropy of only 0.4.

According to the data on the percentage of swing to stride, the waveform entropy of the ALS group is higher, indicating larger fluctuations in the percentage of the swing phase in gait. For example, the left swing interval (% of stride) data point for the ALS group may be 0.6, while the Ctrl may be 0.4. During the support phase, the waveform entropy of the ALS group is relatively high, and the unevenness of the support phase time is significant. For example, the waveform entropy of a data point (such as ALS left stance interval) may be 0.75, while the waveform entropy of the Ctrl is 0.3.

Similar to the stance interval, the waveform entropy of support as a percentage of stride may also be high, indicating gait instability. For example, the right stance interval (% of stride) for the ALS group may be 0.7, while for the Ctrl it may be 0.5. It can be seen that the stride interval waveform entropy of the ALS group is higher, especially on the right stride, indicating greater instability in stride, which may be caused by the lack of motor coordination in ALS patients. The waveform entropy of the Ctrl is lower, indicating that the stride interval is relatively stable and the gait is more regular. The ratio of left and right signals has a low wavelet entropy and is positively distributed with the original signal, indicating that the ratio of left and right signals can reflect the disease characteristics of the original data and is relatively simple.

**Figure 6:**
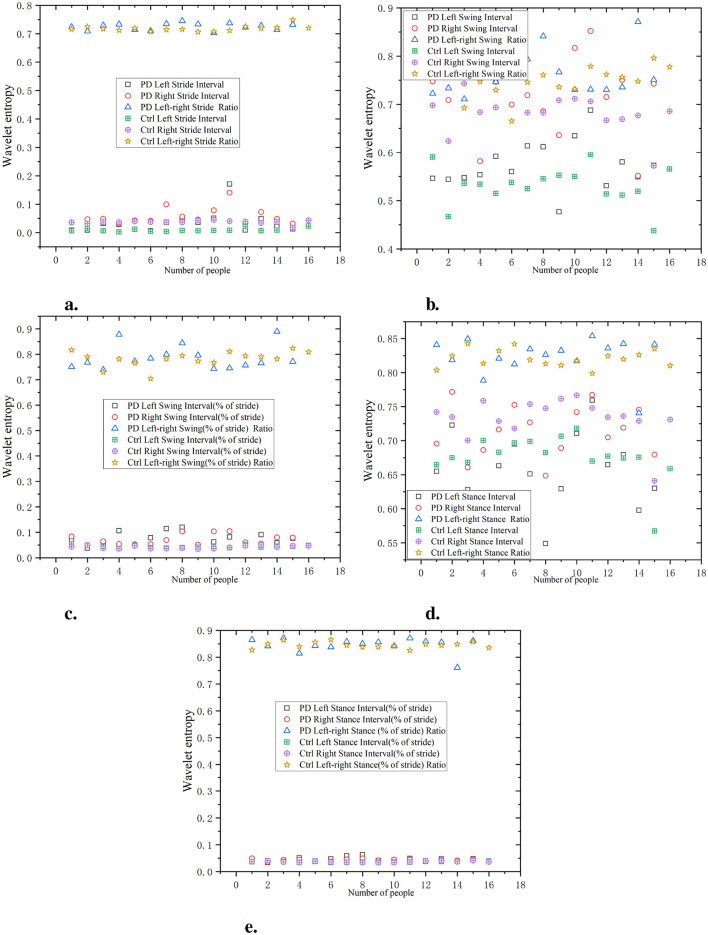
Comparison of wavelet entropy characteristics between Ctrl and PD. **(a)** Stride interval. **(b)** Swing interval. **(c)** Swing interval of stride. **(d)** Stance interval. **(e)** Stance interval of stride.

As shown in Figure 6, the waveform entropy of the PD group at stride intervals is usually higher, indicating greater gait instability in this group. For example, the data point of PD Left Stride Interval may be 0.7, while the stride waveform entropy of the Ctrl is 0.3, indicating that PD have significant fluctuations in stride control.

The waveform entropy of the swing interval shows a larger change in the PD group, while the Ctrl has lower waveform entropy, indicating more stable swing intervals in the Ctrl. For example, the waveform entropy of PD left swing interval may be 0.75, while the waveform entropy of the swing interval in the Ctrl may be 0.45, indicating that PD have significant irregularity during the swing phase.

The PD group usually has a higher waveform entropy in the proportion of swing, indicating a greater fluctuation in the proportion of swing in gait. Assuming a certain data point shows that the waveform entropy of PD left swing interval (% of stride) is 0.8, while the Ctrl is 0.5, this indicates larger variations in the proportion of swing during gait in the PD.

The waveform entropy of the stance interval is higher in the PD group, indicating uneven timing in the stance phase. For example, the waveform entropy of PD left stance interval may be 0.75, while the waveform entropy of the Ctrl is 0.4, indicating gait instability in PD during the support phase.

The waveform entropy of the percentage of stance within the stride demonstrates larger data fluctuations in the PD, indicating irregularities in the proportion of stance stages in gait. Assuming a certain data point shows that the waveform entropy of PD left stance interval (% of stride) is 0.85, while the Ctrl is 0.6, it indicates that the proportion of stance in the PD group changes significantly during gait. The wavelet entropy of the ratio of left-to-right signals is lower and positively correlated with the original signals, indicating that the ratio of left and right signals can reflect the disease characteristics of the original data and is relatively simple.

**Figure 7:**
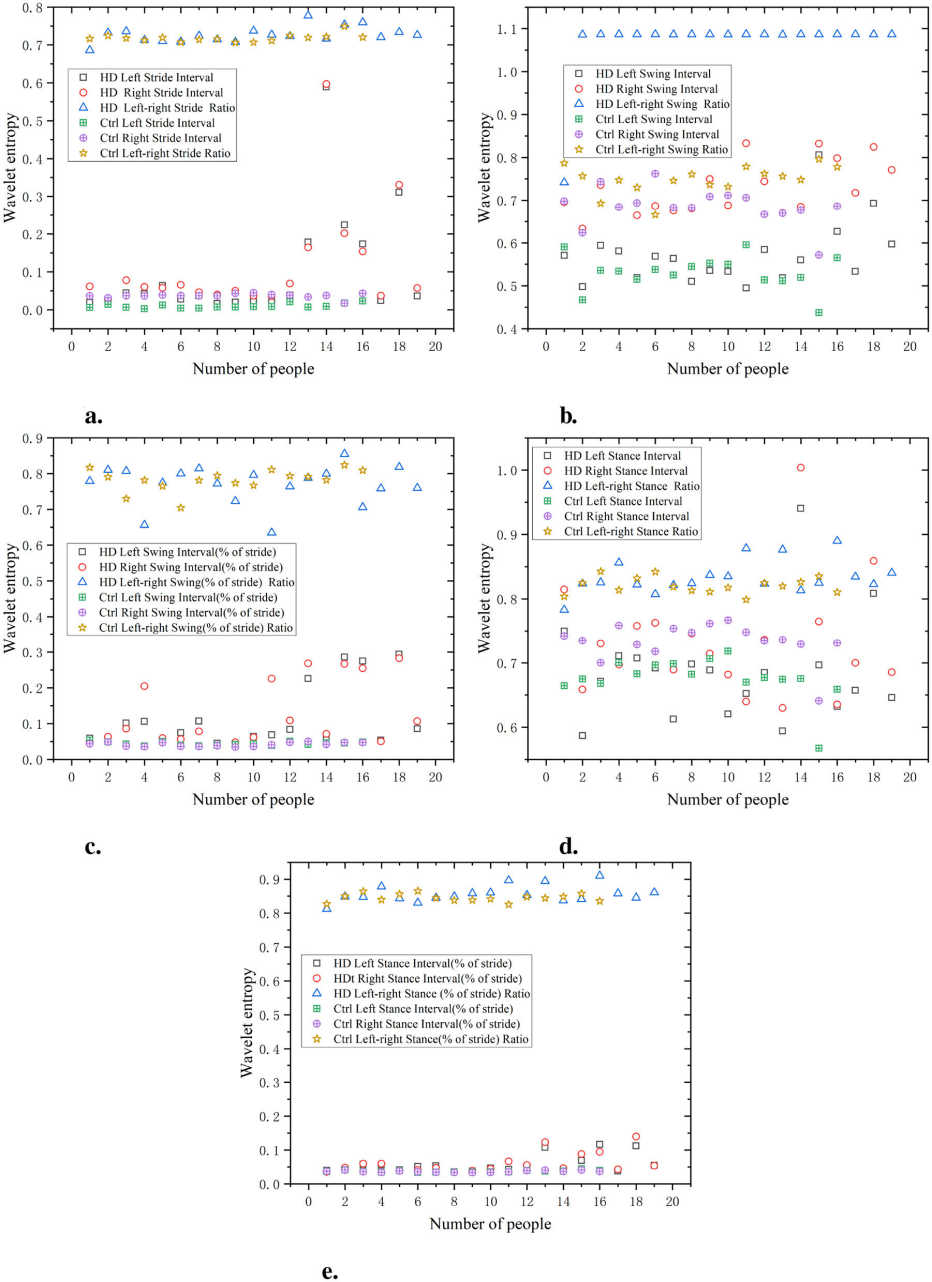
Comparison of wavelet entropy characteristics between Ctrl and HD patients. **(a)** Stride interval. **(b)** Swing interval. **(c)** Swing interval of stride. **(d)** Stance interval. **(e)** Stance interval of stride.

As shown in Figure 7 the waveform entropy of the HD group is generally higher at various stages of stride interval, swing interval, and support interval. This indicates that the gait of the HD group exhibits significant variability at various stages (stride, swing, support, etc.), manifesting as instability. The specific data, such as waveform entropy for stride intervals of 0.65 and 0.6, swing intervals of 0.9 and 0.85, and support intervals of 0.8 and 0.75, indicate irregularities and significant fluctuations in gait. Compared with the HD group, the waveform entropy of the Ctrl was lower (e.g. stride intervals of 0.3 and 0.35, swing intervals of 0.4 and 0.45, and support intervals of 0.45 and 0.5), indicating that the gait of the Ctrl was more stable and consistent. The gait of the Ctrl showed minimal fluctuations in each stage, demonstrating good coordination and stability. The ratio of left and right signals has a low wavelet entropy and is positively distributed with the original signal, indicating that the ratio of left and right signals can reflect the disease characteristics of the original data and is relatively simple.

### 3.3 Wavelet coefficients

Given that the nonlinear dynamical characteristics mentioned earlier are derived from a physical perspective, they provide the intrinsic characteristic parameters of gait signals. In recent years, with the development of wavelet transform theory and technology, wavelet transform has been widely applied in the medical field. Because some lesions in patients with neurodegenerative diseases may manifest as tiny changes in an instant, wavelet transform can combine time and frequency for better results. The gait signals of patients with neurodegenerative diseases can be transformed into wavelet transform coefficients through different scales and displacements, which can improve the discrimination results in detecting neurodegenerative diseases. The calculation formula is as follows:


(4) $$\begin{array}{l} f\left(t\right)=\overset{N}{\underset{k=1}{\sum }}\underset{n\in \mathbb{Z}}{\sum }d_{n}^{k}J_{N,n}+\underset{n\in \mathbb{Z}}{\sum }c_{n}^{k}h_{N,n} \end{array}$$

where $d_{n}^{k}=\left(f\left(t\right),J_{N,n}\right)$ is the wavelet coefficient; $c_{n}^{k}=\left(f\left(t\right),j_{N,n}\right)$ is the scale coefficient.

Through the calculation of wavelet coefficients, the distribution of characteristic numerical values is shown in Table 6. In terms of left step length intervals, the wavelet coefficient for the ALS group was 0.0237 ± 0.0302, indicating relatively high variability in step length intervals. This suggests poor consistency in gait patterns among individuals in this group, which may reflect movement disorders associated with ALS. The coefficient for the PD group was 0.0088 ± 0.0103. Compared with the ALS group, their gait patterns were more stable, but still showed some irregularities. The coefficient for the HD group was 0.0154 ± 0.0172. The gait patterns were slightly unstable, showing some irregularities, which may be related to neuromuscular control issues associated with HD syndrome. The coefficient for the Ctrl group was 0.0039 ± 0.0031, the lowest among the four groups. This indicates that the gait patterns were relatively consistent and symmetrical, with stable step length intervals.

**Table 6:** Wavelet coefficients (mean ± standard deviation).

**Gait data**	**ALS**	**PD**	**HD**	**Ctrl**
**Left stride interval**	**0.0237 ± 0.0302**	**0.0088 ± 0.0103**	**0.0154 ± 0.0172**	**0.0039 ± 0.0031**
Right stride interval	0.0177 ± 0.0260	0.0093 ± 0.0080	0.0145 ± 0.0164	0.0029 ± 0.0022
Ratio of left to right stride	0.0088 ± 0.0132	0.0038 ± 0.0043	0.0035 ± 0.005	0.0011 ± 0.0005
Left waggle interval	0.0037 ± 0.0036	0.0047 ± 0.0043	0.0054 ± 0.0048	0.0017 ± 0.0012
Right waggle interval	0.0049 ± 0.0072	0.0042 ± 0.0034	0.0066 ± 0.0065	0.0016 ± 0.0013
Ratio of left to right waggle	0.0128 ± 0.0160	0.0197 ± 0.0141	0.0003 ± 0.0007	0.0021 ± 0.0021
Left swing interval	0.3168 ± 0.3182	0.3085 ± 0.2810	0.3204 ± 0.2813	0.1033 ± 0.1056
Right swing interval	0.3210 ± 0.4677	0.2838 ± 0.2190	0.3571 ± 0.3533	0.1049 ± 0.0721
Ratio of left to right swing	0.0183 ± 0.0251	0.0085 ± 0.0064	0.0101 ± 0.0110	0.0017 ± 0.0017
Left stance interval	0.0195 ± 0.0245	0.0076 ± 0.0076	0.0130 ± 0.0169	0.0029 ± 0.0026
Right stance interval	0.0165 ± 0.0240	0.0083 ± 0.0082	0.0107 ± 0.0140	0.0049 ± 0.0016
Ratio of left to right stance	0.0111 ± 0.0140	0.0053 ± 0.0042	0.0048 ± 0.0065	0.0011 ± 0.0008
Left stance interval	0.3354 ± 0.3704	0.3080 ± 0.2810	0.3204 ± 0.2813	0.1033 ± 0.1056
Right stance interval	0.2203 ± 0.1256	0.2838 ± 0.2190	0.3739 ± 0.3354	0.1100 ± 0.0896
Ratio of left to right stance	0.0050 ± 0.0072	0.0033 ± 0.0024	0.0029 ± 0.0026	0.0009 ± 0.0007

In terms of step length ratio, the wavelet coefficient for the ALS group was 0.0088 ± 0.0132, indicating asymmetry in gait and larger differences in step lengths between the left and right sides. The coefficient for the PD group was 0.0038 ± 0.0043, slightly lower than that of the ALS group, showing better gait symmetry. The coefficient for the HD group was 0.0035 ± 0.005, similar to the PD group, showing better gait symmetry. The coefficient for the Ctrl group was 0.0011 ± 0.0005, showing the least complexity and the most symmetrical gait.

Regarding left support intervals, the wavelet coefficient for the ALS group was 0.0195 ± 0.0245, showing significant variability, indicating irregular gait patterns. The coefficient for the PD group was 0.0076 ± 0.0076, with a relatively stable gait but still showing some irregularities compared to other groups. The coefficient for the HD group was 0.0130 ± 0.0169, with slightly smaller differences in step length intervals than the PD group, but still showing some irregularities. The coefficient for the Ctrl group was 0.0029 ± 0.0026, with a relatively consistent and symmetrical gait and stable step length intervals.

For stance interval, the ALS group had a stance interval of 0.0799 ± 0.0054, showing high complexity and variability. The stance interval of the PD group is 0.0728 ± 0.0034, and the gait difference is relatively small. The stance interval of the HD group is 0.1253 ± 0.0881, with a significant difference. The stance interval of the Ctrl group is 0.0417 ± 0.0054, showing the smallest gait difference and strong gait consistency.

**Figure 8:**
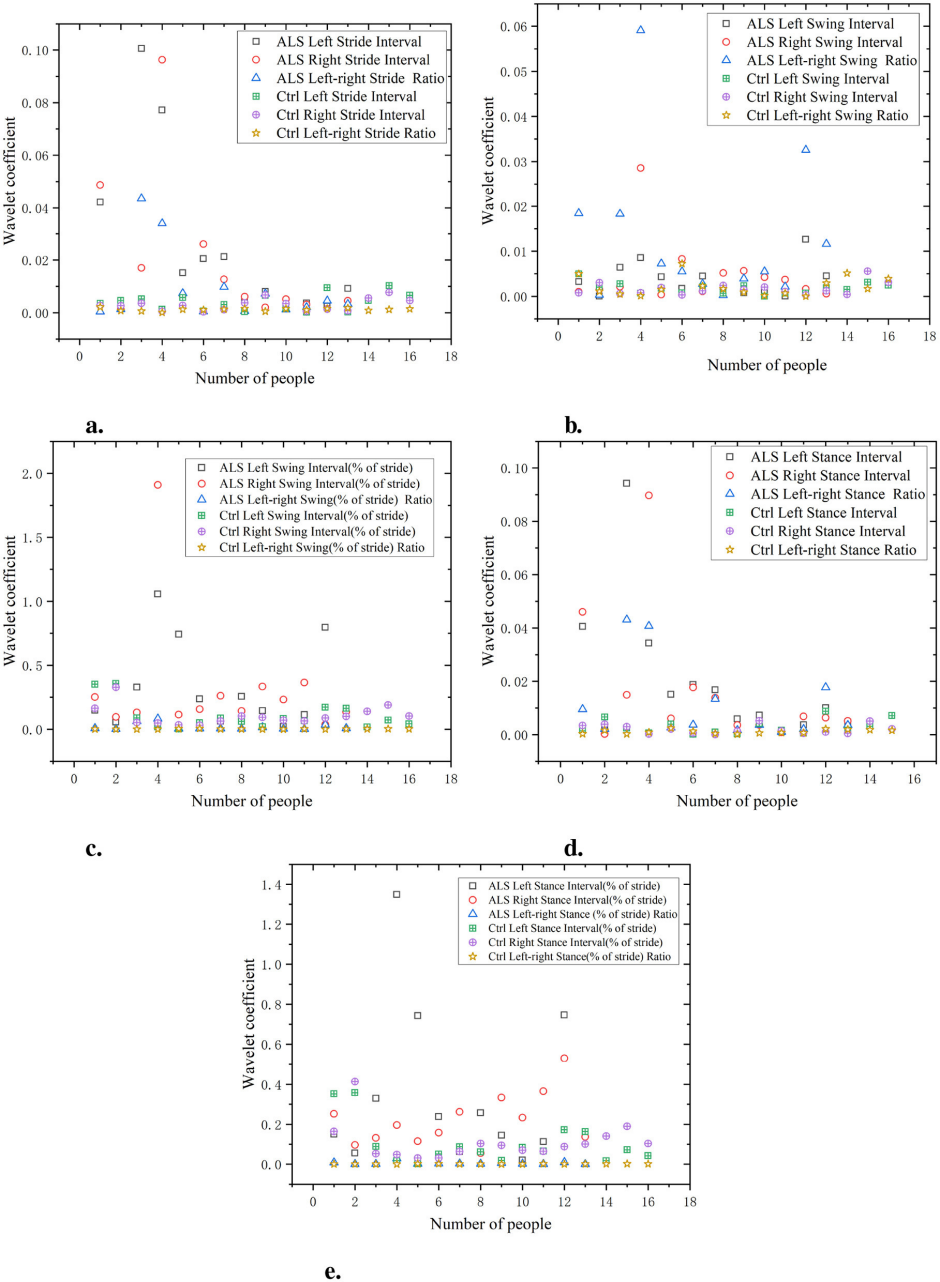
Comparison of wavelet coefficient characteristics between Ctrl and ALS patients. **(a)** Stride interval. **(b)** Swing interval. **(c)** Swing interval of stride. **(d)** Stance interval. **(e)** Stance interval of stride.

As shown in Figure 8, the waveform coefficients of the ALS group are generally high in all data, especially in data such as swing interval, stride interval, and support interval, where the waveform coefficients fluctuate greatly. The ALS group showed significant gait instability, indicating difficulties in gait control and high variability in gait. For example, the waveform coefficients of ALS left swing interval and ALS right swing interval are 0.06 and 0.05, respectively, which are much higher than those of the Ctrl (0.03 and 0.02). The waveform coefficient of the Ctrl is lower, indicating that its gait is more stable and has smaller fluctuations in each stage. The gait of the Ctrl is more regular, with smaller changes observed in different stages of gait (stride, swing, stance, etc.). For example, the waveform coefficients of ctrl left swing interval and ctrl right swing interval are 0.03 and 0.02, respectively, which are significantly lower than those of the ALS group. The wavelet coefficients of the ratio of left and right signals are low and positively distributed with the original signal, indicating that the ratio of left and right signals can reflect the disease characteristics of the original data and is relatively simple.

**Figure 9:**
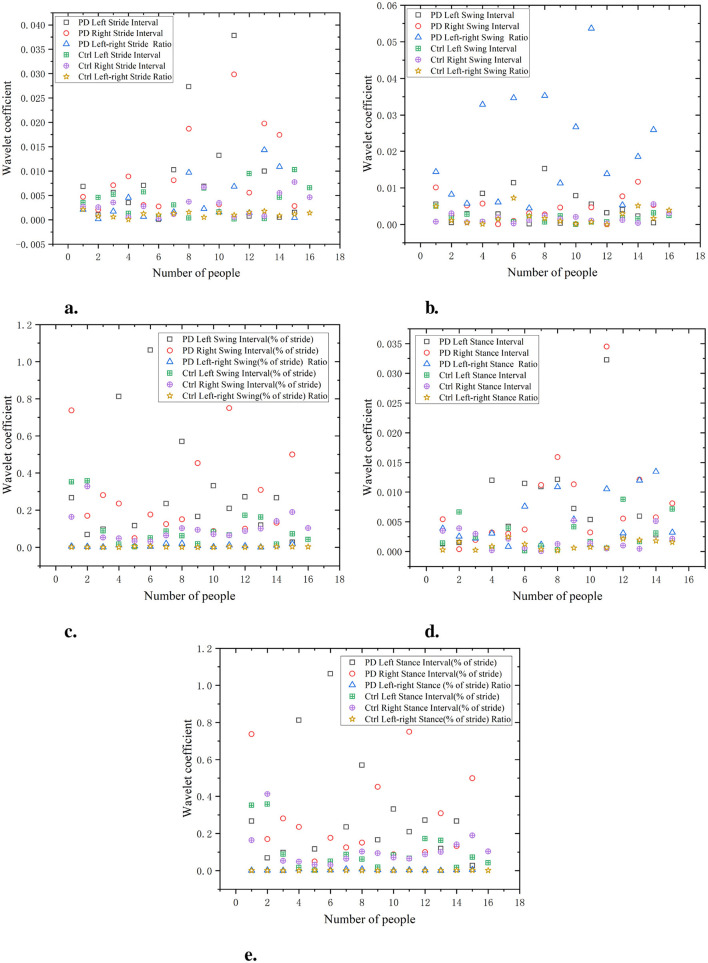
Comparison of wavelet coefficient characteristics between Ctrl and PD. **(a)** Stride interval. **(b)** Swing interval. **(c)** Swing interval of stride. **(d)** Stance interval. **(e)** Stance interval of stride.

As shown in Figure 9, the waveform coefficients of the PD group are generally high, especially in data such as swing interval, stride interval, and stance interval. The fluctuation of waveform coefficients is large, indicating that their gait is unstable, which may be related to the movement disorders and gait control ability of PD. For example, the waveform coefficient of PD left swing interval is 0.055, significantly higher than that of the Ctrl (0.025). The waveform coefficients of the Ctrl are generally lower, indicating a more stable gait. For example, the waveform coefficient of Ctrl left stride interval is 0.015, showing small fluctuations, indicating a more regular gait. The wavelet coefficients of the ratio of left and right signals are low and positively distributed with the original signal, suggesting that the ratio of left-to-right signals can reflect the disease characteristics of the original data in a relatively simple manner.

**Figure 10:**
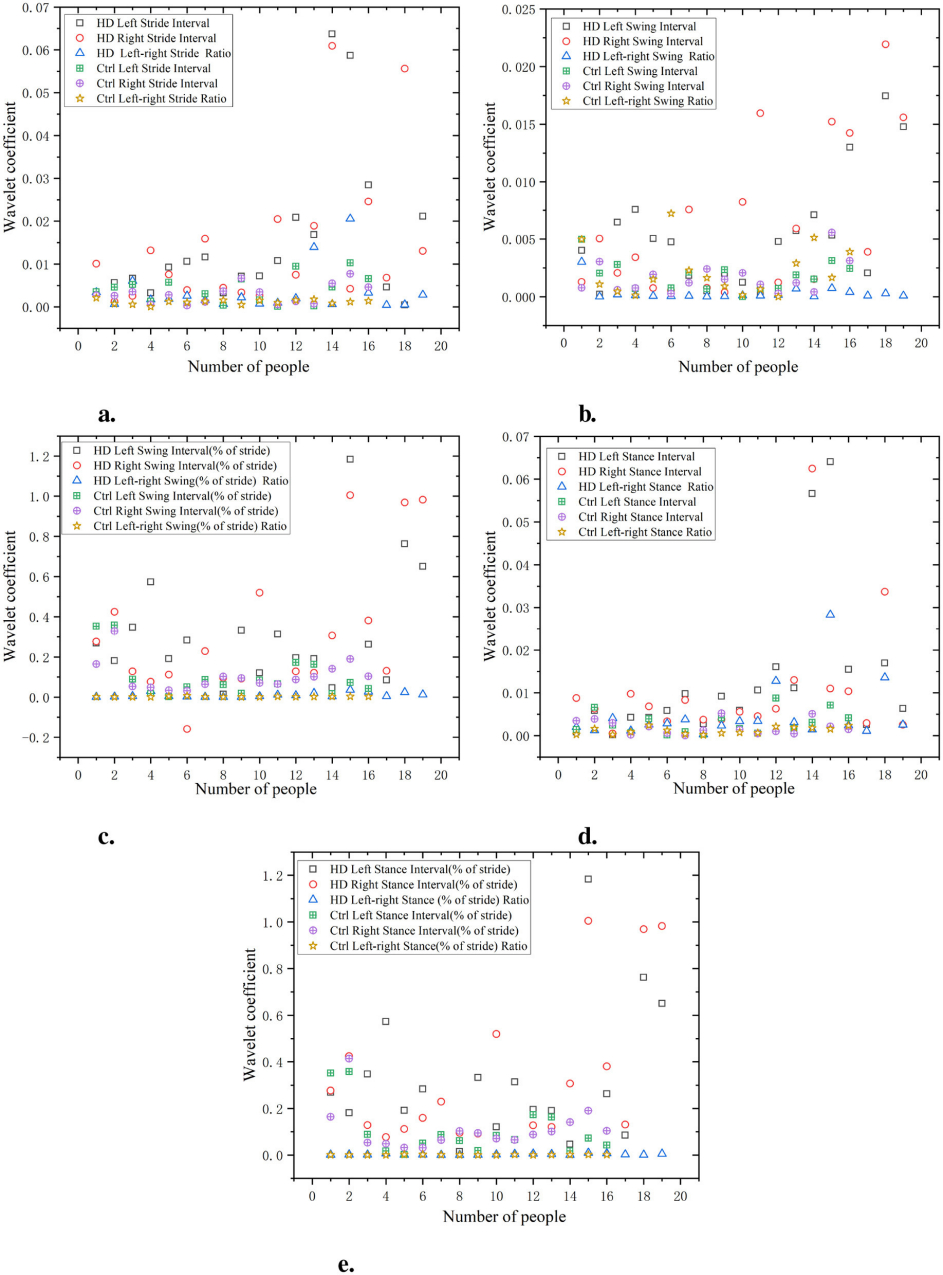
Comparison of wavelet coefficient characteristics between Ctrl and HD patients. **(a)** Stride interval. **(b)** Swing interval. **(c)** Swing interval of stride. **(d)** Stance interval. **(e)** Stance interval of stride.

As shown in Figure 10, the gait of the HD group exhibits high waveform coefficients, especially in data such as stride, swing, and support intervals (for example, stride interval waveform coefficient is 0.065, swing interval waveform coefficient is 0.02, and support interval waveform coefficient is 0.06). This indicates that there are significant fluctuations and instability in the gait stages of the HD group, which may be related to poor motor coordination or the influence of diseases. Compared with the HD group, the Ctrl had lower waveform coefficients (e.g. stride interval waveform coefficient of 0.03, swing interval waveform coefficient of 0.01, and support interval waveform coefficient of 0.03). This indicates that the gait of the Ctrl is more regular, with smaller and more stable changes in each stage of gait. The wavelet coefficients of the ratio of left and right signals are low and positively distributed with the original signal, indicating that the ratio of left and right signals can reflect the disease characteristics of the original data in a relatively simple manner.

## 4 Conclusions

(1) Based on the significant asymmetry of left and right limb movements in neurological diseases, which introduces complexity and unreadability to the analysis of gait data. According to the principles of mechanical kinematics, there is an inherent connection between the two, and a processing method for the ratio of left and right limb data in gait signals is proposed.
(2) Based on the comparison of the mean, standard deviation, and coefficient of variation of the left and right sequences and their ratios between PD, ALS, and HD and a healthy Ctrl, it is found that there is a close correlation between the ratios of left and right sequences and the standard deviations and coefficients of variation of the actual left and right sequences. These ratios of left and right sequences can be used to identify PD, ALS, and HD categories.
(3) After using a median filter (*n* = 3) to filter four sets of stride ratio data (Ctr1, ALS, PD, HD), it is found that the data before filtering (black lines) generally showed significant fluctuations, with many peaks and valleys, indicating that the original data may contain a lot of noise or outliers. The filtered data (red lines) have relatively smaller fluctuations and smoother curves, indicating that the filtering process effectively reduces the noise in the data and makes it more stable.
(4) Comparative analysis of the C0 complexity, sample entropy, wavelet entropy, and wavelet coefficients between PD, ALS, HD and the Ctrl reveals distinct features of the four data sets. The ALS group showed significant differences in stride, swing, and stance intervals, with strong gait asymmetry. The PD group exhibited higher complexity during the swing and stance phases. The HD group shows significant variability in gait complexity, particularly during the swing and stance phases. The gait of the Ctrl group showed the lowest complexity, indicating high regularity and symmetry.
(5) The raw data distribution of the left and right limbs among PD, ALS, HD, and the healthy Ctrl group is relatively large, posing certain difficulties in analyzing the patient's diseases. Using the ratio of left and right data effectively reduces the dispersion of the data. The ranking of CO complexity features from highest to lowest is ALS, HD, PD, and Ctrl. The ranking of sample entropy features from largest to smallest is ALS, HD, PD, and Ctrl. The ranking of wavelet coefficient features from largest to smallest is ALS, PD, HD, and Ctrl.

## Data Availability

The original contributions presented in the study are included in the article/Supplementary material, further inquiries can be directed to the corresponding author.

## References

[1] Ebersbach G Baas H Csoti I Müngersdorf M Deuschl G. Scales in Parkinson's disease. J Neurol. (2006) 253:32–5. 10.1007/s00415-006-4008-0 16944355

[2] Scheid BH Aradi S Pierson RM Baldassano S Tivon I Litt B. Predicting severity of Huntington's disease with wearable sensors. Front Digit Health. (2022) 4:874208. 10.3389/fdgth.2022.874208 35445206 PMC9013843

[3] Ramaker C Marinus J Stiggelbout AM Van Hilten BJ. Systematic evaluation of rating scales for impairment and disability in Parkinson's disease. Mov Disord. (2002) 17:867–76. 10.1002/mds.10248 12360535

[4] Saadion NW Adib MAHM. Experimental study of gait monitoring on wearable shoes insole and analysis: a review. In: International Human Engineering Symposium. Cham: Springer (2023). p. 273–287.

[5] Stebbins GT Goetz CG Burn DJ Jankovic J Khoo TK Tilley BC. How to identify tremor dominant and postural instability/gait difficulty groups with the movement disorder society unified Parkinson's disease rating scale: comparison with the unified Parkinson's disease rating scale. Mov Disord. (2013) 28:668–70. 10.1002/mds.25383 23408503

[6] Hoehn MM Yahr MD. Parkinsonism: onset, progression, and mortality. Neurology. (1967) 17:427–427. 10.1212/WNL.17.5.427 6067254

[7] Goetz CG Tilley BC Shaftman SR Stebbins GT Fahn S Martinez-Martin P. Movement Disorder Society-sponsored revision of the Unified Parkinson's Disease Rating Scale (MDS-UPDRS): scale presentation and clinimetric testing results. Mov Disord. (2008) 23:2129–70. 10.1002/mds.22340 19025984

[8] Rao PS Parida P Sahu G Dash S A. multi-view human gait recognition using hybrid whale and gray wolf optimization algorithm with a random forest classifier. Image Vis Comput. (2023) 136:104721. 10.1016/j.imavis.2023.104721

[9] Uddin MZ Muramatsu D Takemura N Ahad MAR Yagi Y. Spatio-temporal silhouette sequence reconstruction for gait recognition against occlusion. IPSJ Trans Comp Vision Appl. (2019) 11:1–18. 10.1186/s41074-019-0061-3

[10] Kim HW Lee GH Nam WJ Jin KM Kang TK Yang GJ. MHCanonNet: Multi-Hypothesis Canonical lifting Network for self-supervised 3D human pose estimation in the wild video. Pattern Recognit. (2024) 145:109908. 10.1016/j.patcog.2023.109908

[11] Hua R Wang Y. Monitoring insole (MONI): A low power solution toward daily gait monitoring and analysis. IEEE Sens J. (2019) 19:6410–20. 10.1109/JSEN.2019.2910105

[12] Shirakawa T Sugiyama N Sato H Sakurai K Sato E. Gait analysis and machine learning classification on healthy subjects in normal walking. Int J Parall Emerg Distrib Syst. (2017) 32:185–94. 10.1080/17445760.2015.1044007

[13] Kim YJ Park I Choi HC Ahn ME Ryu OH Jang D. Relationship of neural correlates of gait characteristics and cognitive dysfunction in patients with mild cognitive impairment. J Clin Med. (2023) 12:5347. 10.3390/jcm12165347 37629389 PMC10455461

[14] Rashidi FRM Hussein O Hasan W. Investigation on developing of a piezoresistive pressure sensor for foot plantar measurement system. In: 2015 IEEE Regional Symposium on Micro and Nanoelectronics (RSM). Kuala Terengganu: IEEE (2015). p. 1–4.

[15] Sawant N Vaidya S. Reliability of OHM 3000 plantar pressure system for measure-ment of plantar pressures in healthy Indian population. Int J Physioth Res. (2022) 10:4095–101. 10.16965/ijpr.2021.210

[16] Zhao S Liu R Fei C Zia AW Jing L. Flexible sensor matrix film-based wearable plantar pressure force measurement and analysis system. PLoS ONE. (2020) 15:e0237090. 10.1371/journal.pone.0237090 32764796 PMC7413492

[17] Murray MP Drought AB Kory RC. Walking patterns of normal men. JBJS. (1964) 46:335–60. 10.2106/00004623-196446020-00009 14129683

[18] Murray MP. Gait as a total pattern of movement: Including a bibliography on gait. Am J Phys Med Rehabil. (1967) 46:290–333. 5336886

[19] Hausdorff J Mitchell S Firtion R Peng C Cudkowicz M Wei J. Altered fractal dynamics of gait: reduced stride-interval correlations with aging and Huntington's disease. J Appl Physiol. (1997) 82:262–9. 10.1152/jappl.1997.82.1.262 9029225

[20] Lipsitz LA. Dynamics of stability: the physiologic basis of functional health and frailty. J Gerontol Series A: Biol Sci Med Sci. (2002) 57:B115–25. 10.1093/gerona/57.3.B115 11867648

[21] Mulligan VK Chakrabartty A. Protein misfolding in the late-onset neurodegenerative diseases: common themes and the unique case of amyotrophic lateral sclerosis. Proteins: Struct Funct Bioinform. (2013) 81:1285–303. 10.1002/prot.24285 23508986

[22] Mead S Reilly MM A. new prion disease: relationship with central and peripheral amyloidoses. Nat Rev Neurol. (2015) 11:90–7. 10.1038/nrneurol.2014.263 25623792

[23] Morris ME McGinley J Huxham F Collier J Iansek R. Constraints on the kinetic, kinematic and spatiotemporal parameters of gait in Parkinson's disease. Hum Mov Sci. (1999) 18:461–83. 10.1016/S0167-9457(99)00020-2

[24] Nakagawa K Kanai S Kitakaze S Okamura H. Discriminant accuracy of standing balance tests for the level of gait dependency in hospitalized patients with Alzheimer's disease. Dement Geriatr Cogn Disord. (2024) 53:135–42. 10.1159/000538541 38599186

[25] Ranchet M Hoang I Derollepot R Paire-Ficout L. Between-sessions test-retest reliability of prefrontal cortical activity during usual walking in patients with Parkinson's Disease: a fNIRS study. Gait & *Posture*. (2023) 103:99–105. 10.1016/j.gaitpost.2023.05.003 37156165

[26] Aziz W Arif M. Complexity analysis of stride interval time series by threshold dependent symbolic entropy. Eur J Appl Physiol. (2006) 98:30–40. 10.1007/s00421-006-0226-5 16841202

[27] Schache AG Dorn TW Blanch PD Brown NA Pandy MG. Mechanics of the human hamstring muscles during sprinting. Med Sci Sports Exerc. (2012) 44:647–58. 10.1249/MSS.0b013e318236a3d2 21912301

[28] Riches SP Spencer B Jones T Clay M Bush T Image I. Park and Stride for Health and Wellbeing: Evaluation of a wayfinding intervention to promote active travel to school in Oxfordshire, UK. J Transp Health. (2024) 35:101769. 10.1016/j.jth.2024.101769

[29] Tytgat O Fauvart M Stakenborg T Deforce D Van Nieuwerburgh F. STRide probes: single-labeled short tandem repeat identification probes. Biosens Bioelectron. (2021) 180:113135. 10.1016/j.bios.2021.113135 33690100

[30] Werneck LC Bezerra R Silveira Neto Od Scola RH. A clinical epidemiological study of 251 cases of amyotrophic lateral sclerosis in the south of Brazil. Arquivos de neuro-psiquiatria. (2007) 65:189–95. 10.1590/S0004-282X2007000200001 17607412

[31] Umar TP Jain N Papageorgakopoulou M Shaheen RS Alsamhori JF Muzzamil M. Artificial intelligence for screening and diagnosis of amyotrophic lateral sclerosis: a systematic review and meta-analysis. Amyotroph Lateral Scler Front Degener. (2024) 25:425–36. 10.1080/21678421.2024.2334836 38563056

[32] Wu Y Shi L. Analysis of altered gait cycle duration in amyotrophic lateral sclerosis based on nonparametric probability density function estimation. Med Eng Phys. (2011) 33:347–55. 10.1016/j.medengphy.2010.10.023 21130016

[33] Dubbioso R Spisto M Hausdorff JM Aceto G Iuzzolino VV Senerchia G. Cognitive impairment is associated with gait variability and fall risk in amyotrophic lateral sclerosis. Eur J Neurol. (2023) 30:3056–67. 10.1111/ene.15936 37335396

[34] Hausdroff J. Gait variability: methods, modeling and meaning. J Neuro Eng Rehabil. (2005) 20:1–9. 10.1186/1743-0003-2-19 16033650 PMC1185560

[35] Hausdorff JM Lertratanakul A Cudkowicz ME Peterson AL Kaliton D Goldberger AL. Dynamic markers of altered gait rhythm in amyotrophic lateral sclerosis. J Appl Physiol. (2000). 10.1152/jappl.2000.88.6.2045 10846017

[36] Bahureksa L Najafi B Saleh A Sabbagh M Coon D Mohler MJ. The impact of mild cognitive impairment on gait and balance: a systematic review and meta-analysis of studies using instrumented assessment. Gerontology. (2016) 63:67–83. 10.1159/000445831 27172932 PMC5107359

