# Supplementary figures and images for: Nonlinear characteristics of gait signals in neurodegenerative diseases
Source: Front Neurol. 2025 Jun 16;16:1607273. doi: 10.3389/fneur.2025.1607273 (PMC12206782; doi:10.3389/fneur.2025.1607273)

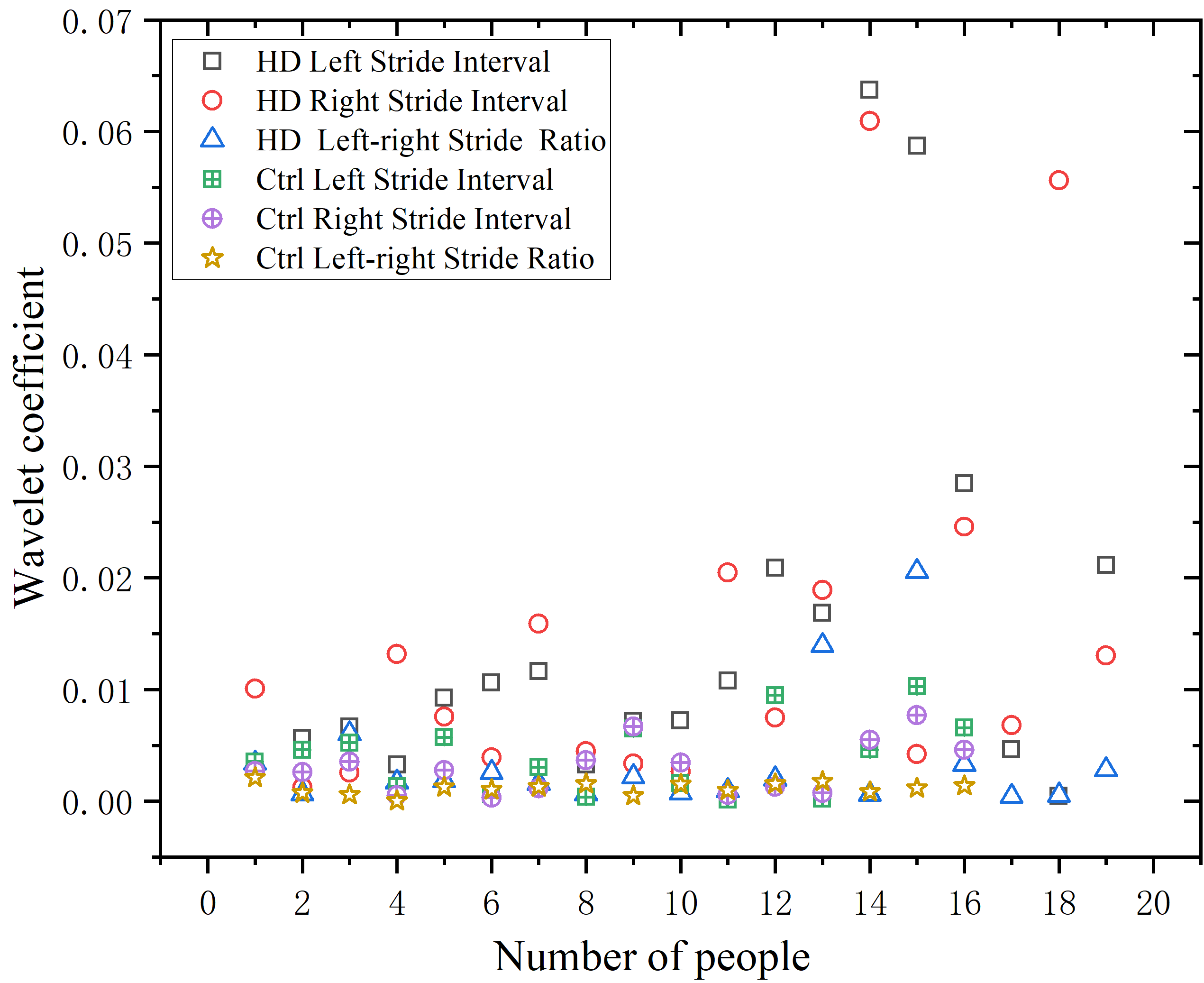

Supplement: Supplementary file 2 [file Data_Sheet_2.zip › Data Sheet 1/10a.png]

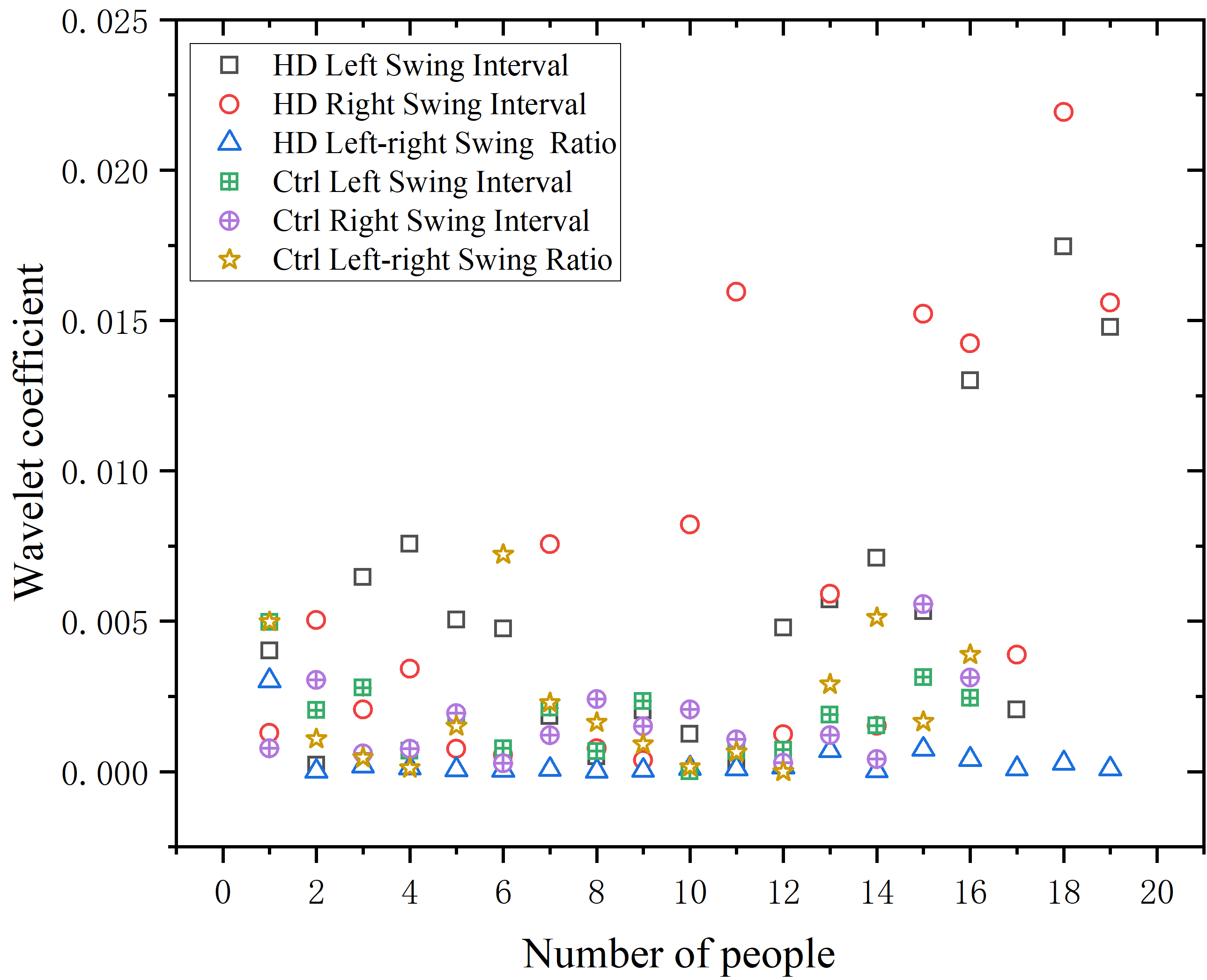

Supplement: Supplementary file 2 [file Data_Sheet_2.zip › Data Sheet 1/10b.png]

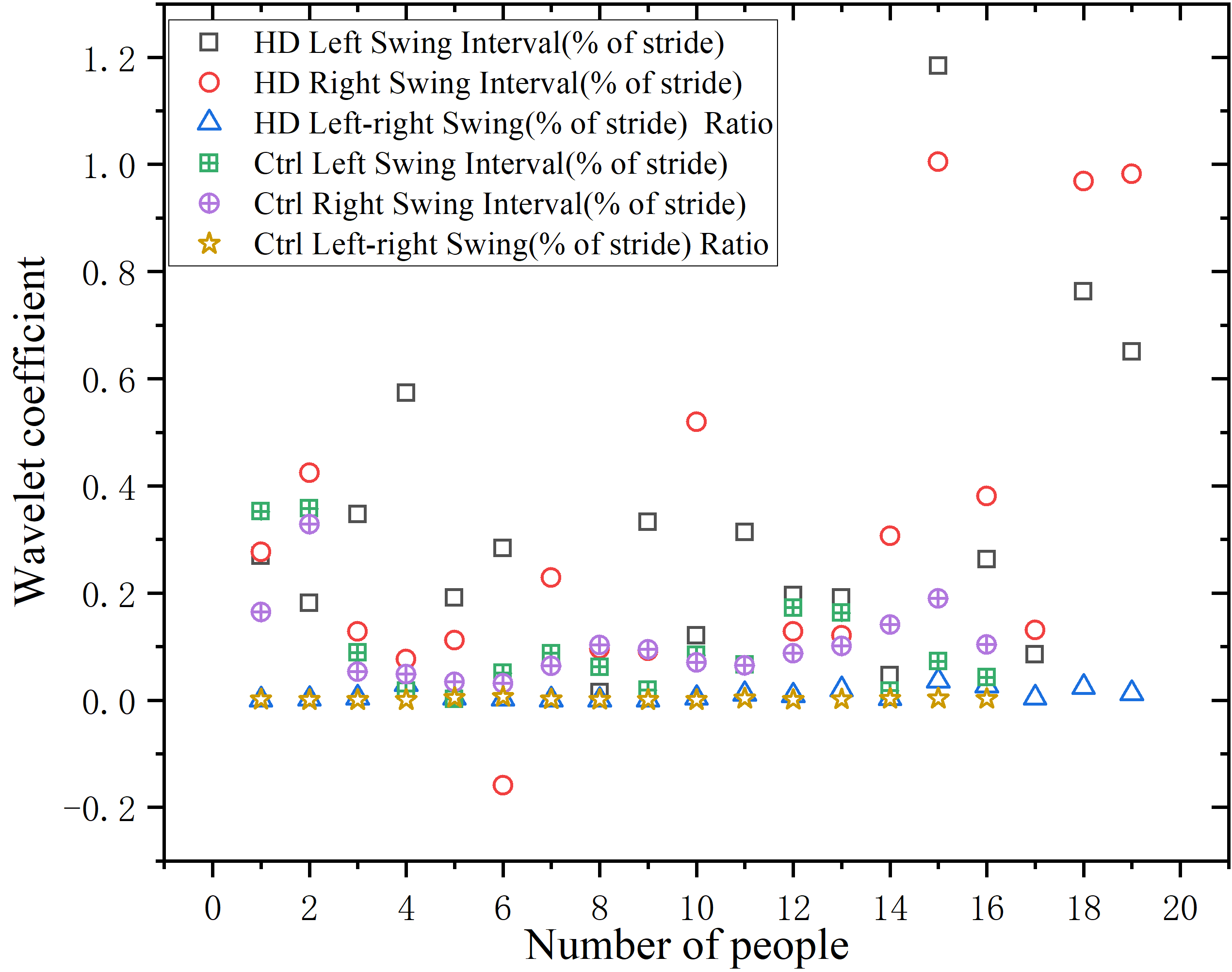

Supplement: Supplementary file 2 [file Data_Sheet_2.zip › Data Sheet 1/10c.png]

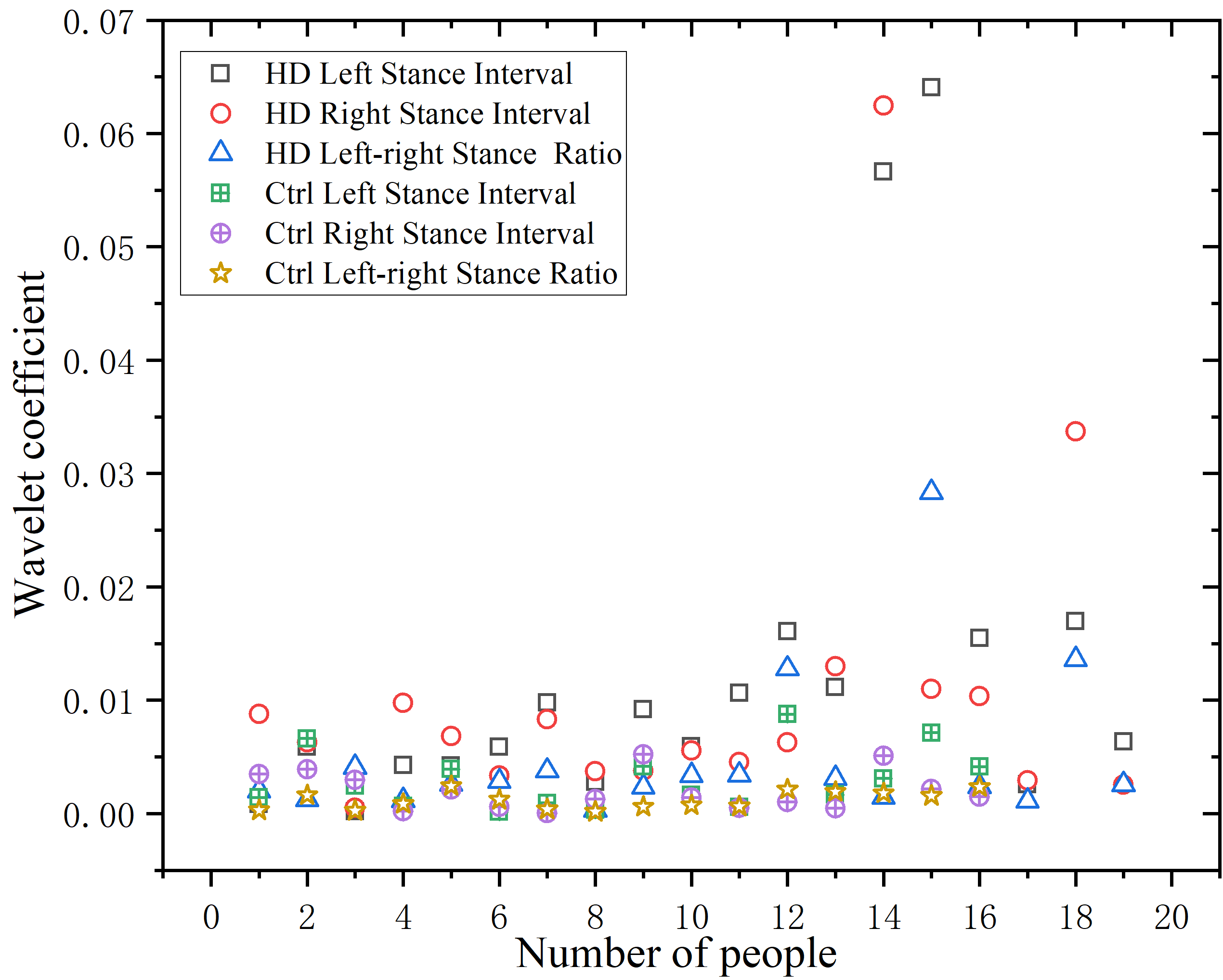

Supplement: Supplementary file 2 [file Data_Sheet_2.zip › Data Sheet 1/10d.png]

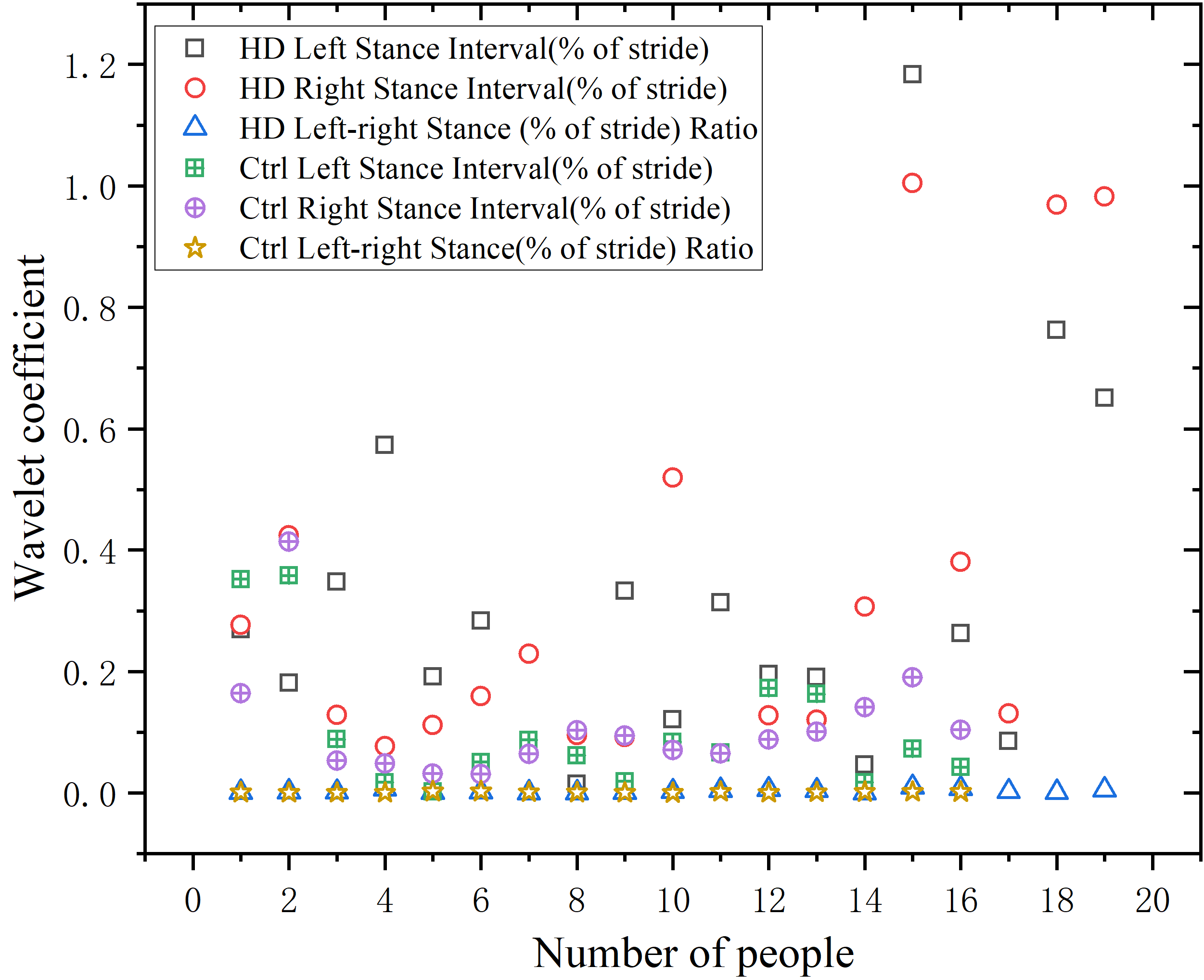

Supplement: Supplementary file 2 [file Data_Sheet_2.zip › Data Sheet 1/10e.png]

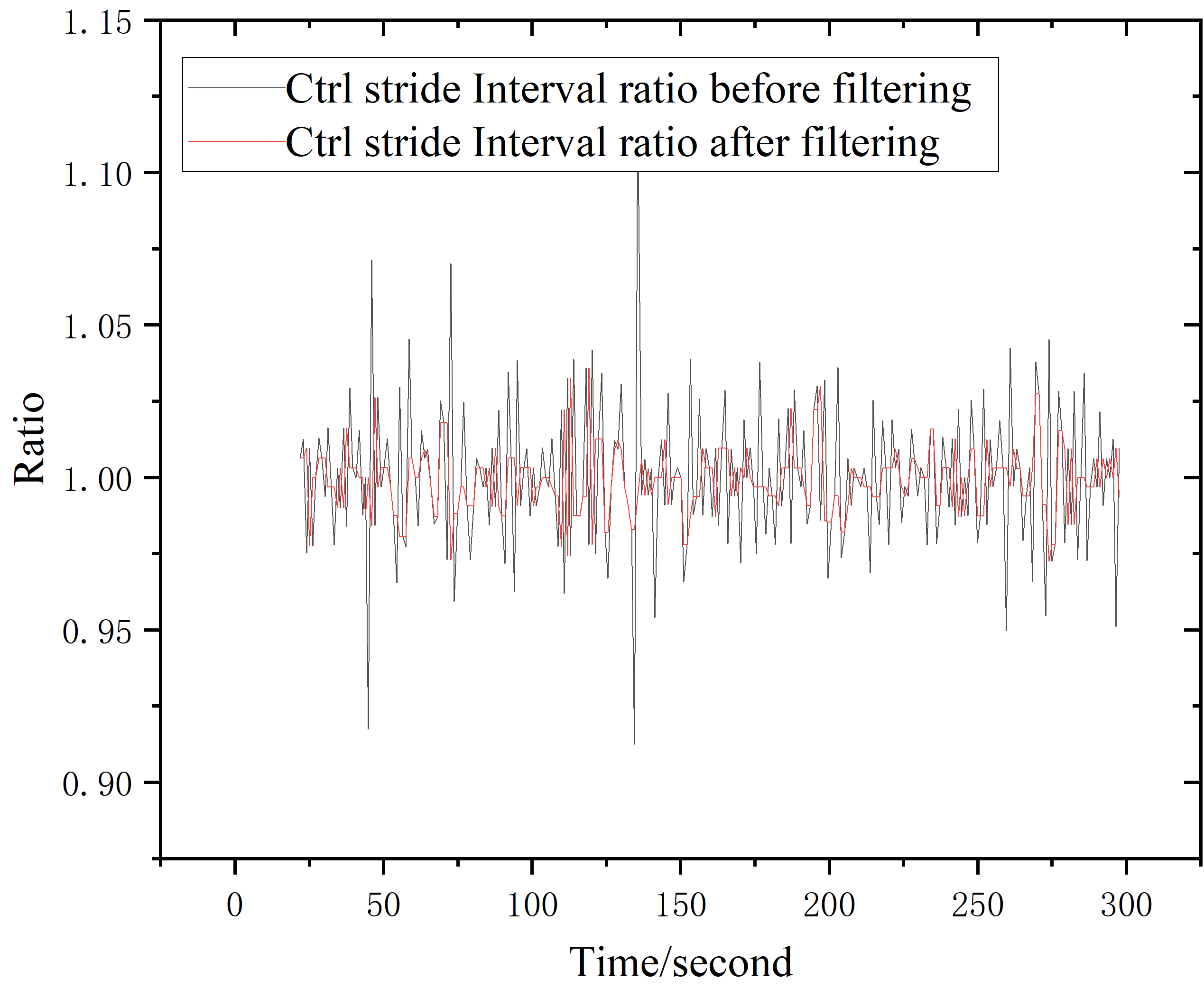

Supplement: Supplementary file 2 [file Data_Sheet_2.zip › Data Sheet 1/1a.png]

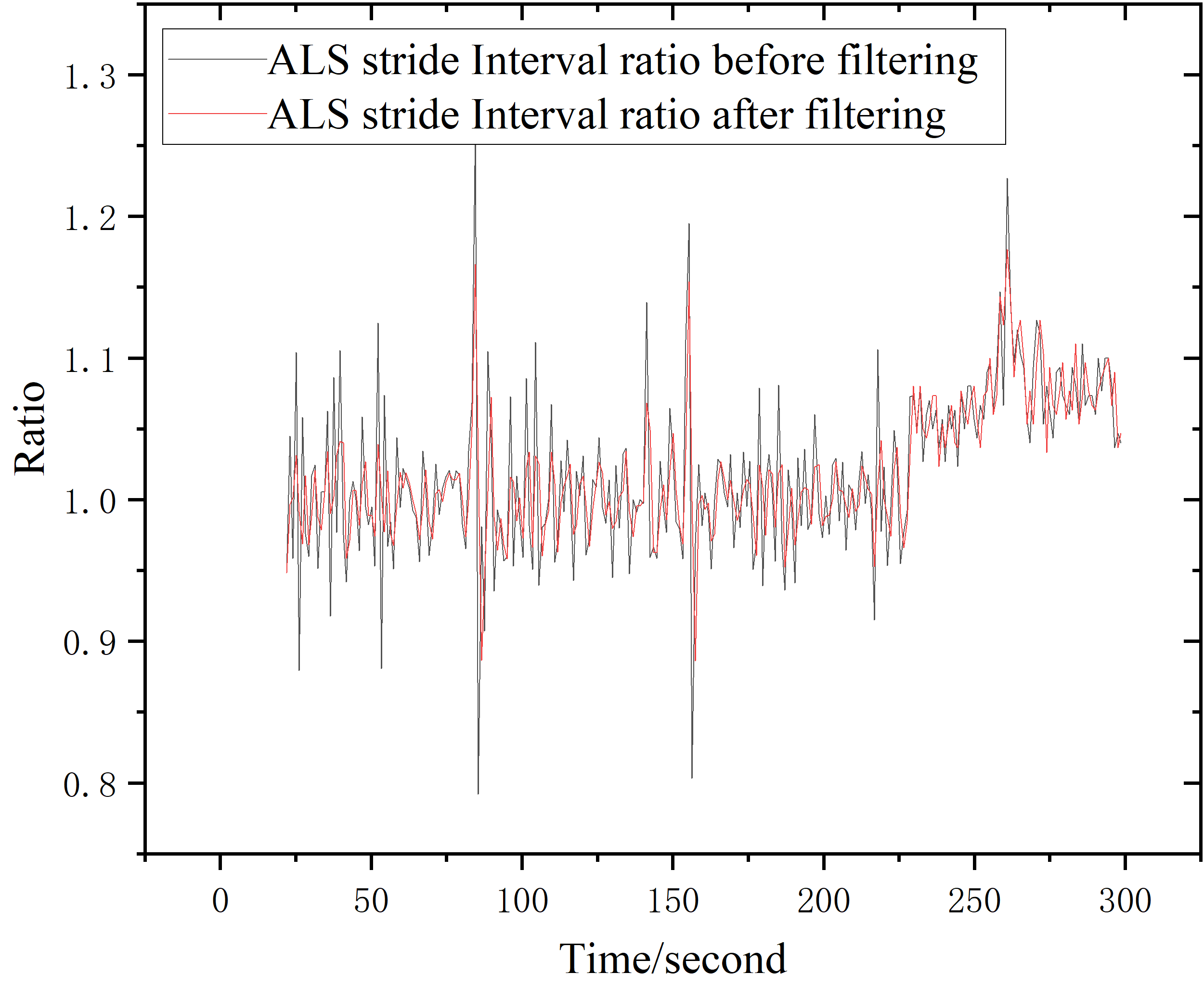

Supplement: Supplementary file 2 [file Data_Sheet_2.zip › Data Sheet 1/1b.png]

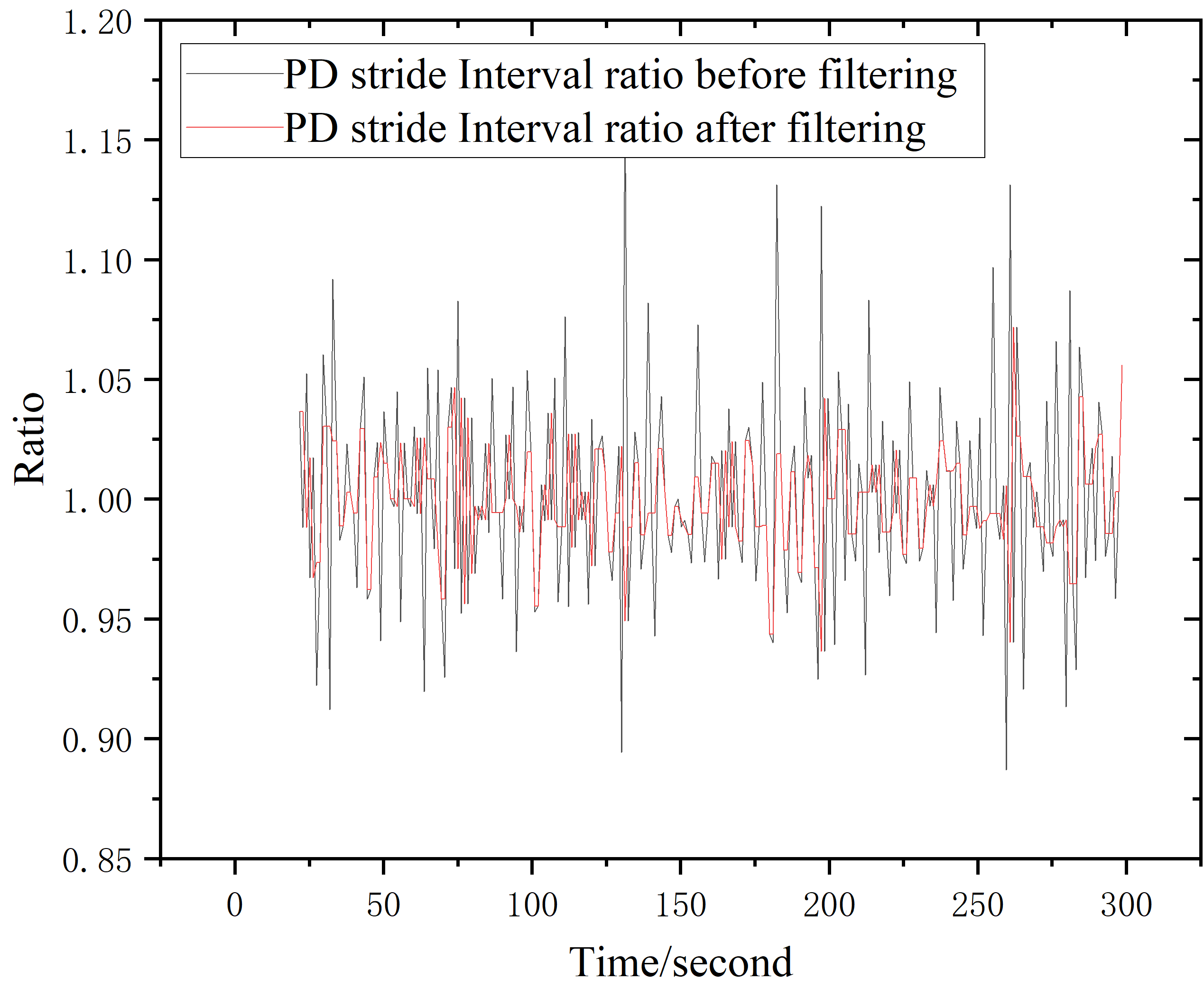

Supplement: Supplementary file 2 [file Data_Sheet_2.zip › Data Sheet 1/1c.png]

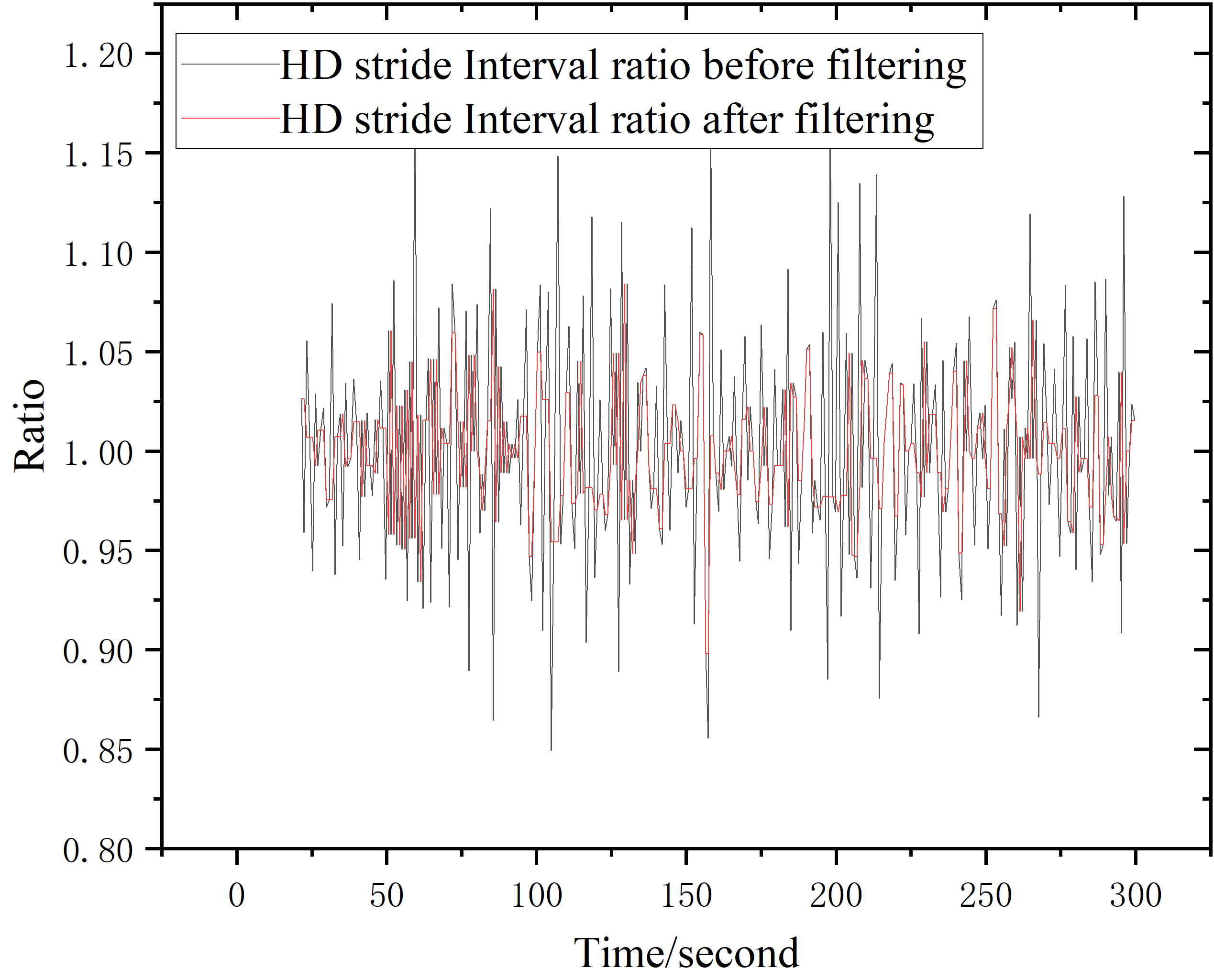

Supplement: Supplementary file 2 [file Data_Sheet_2.zip › Data Sheet 1/1d.png]

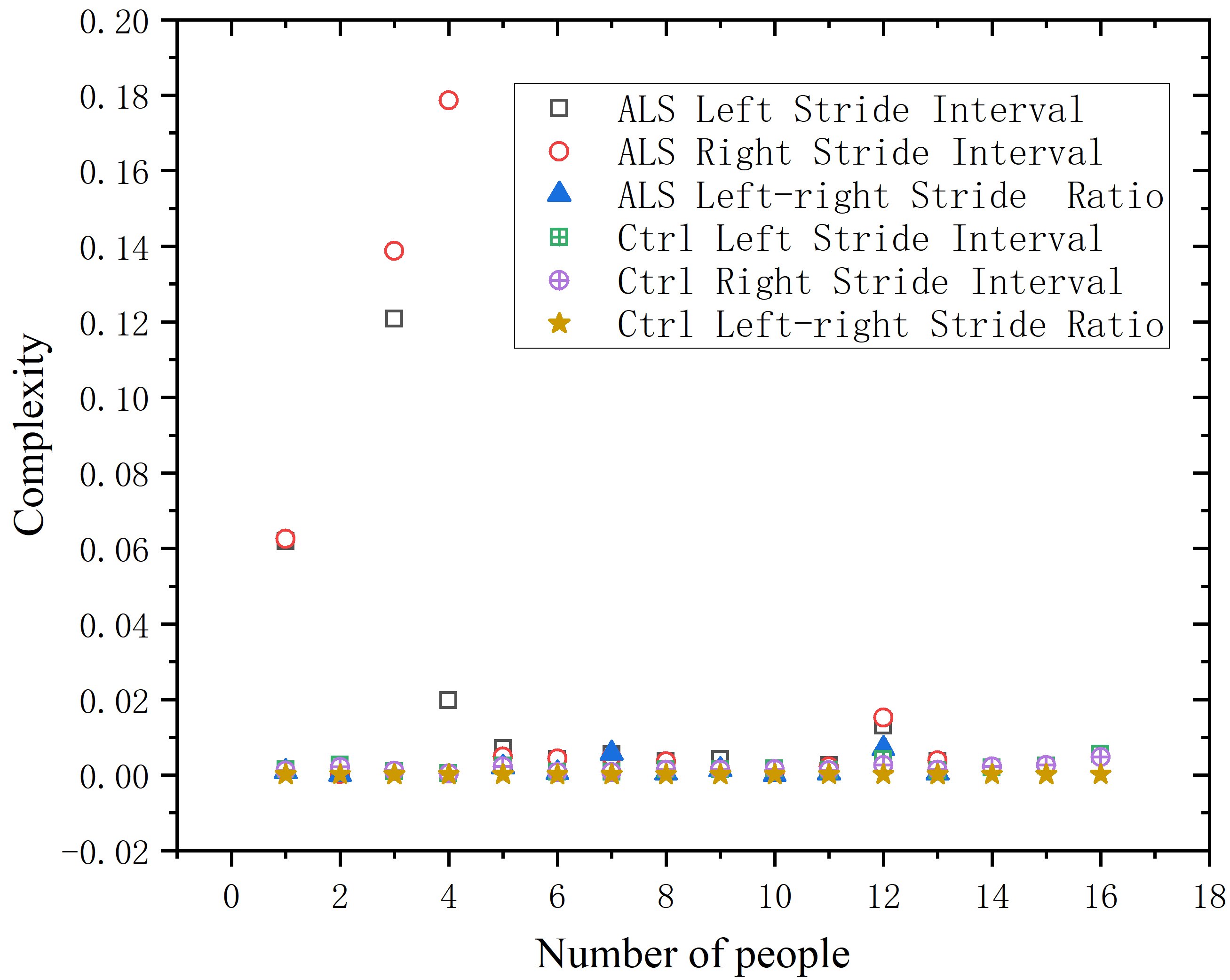

Supplement: Supplementary file 2 [file Data_Sheet_2.zip › Data Sheet 1/2a.png]

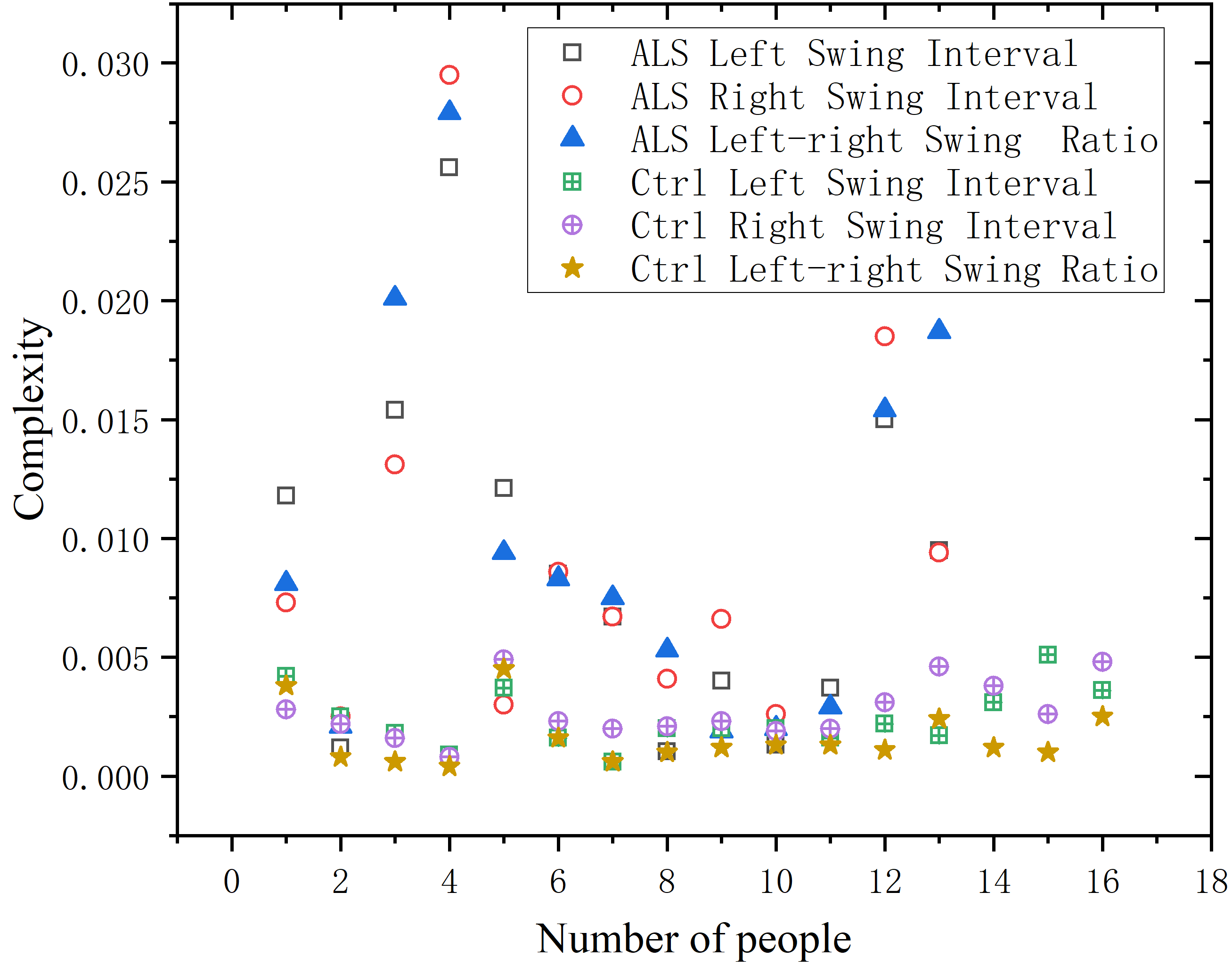

Supplement: Supplementary file 2 [file Data_Sheet_2.zip › Data Sheet 1/2b.png]

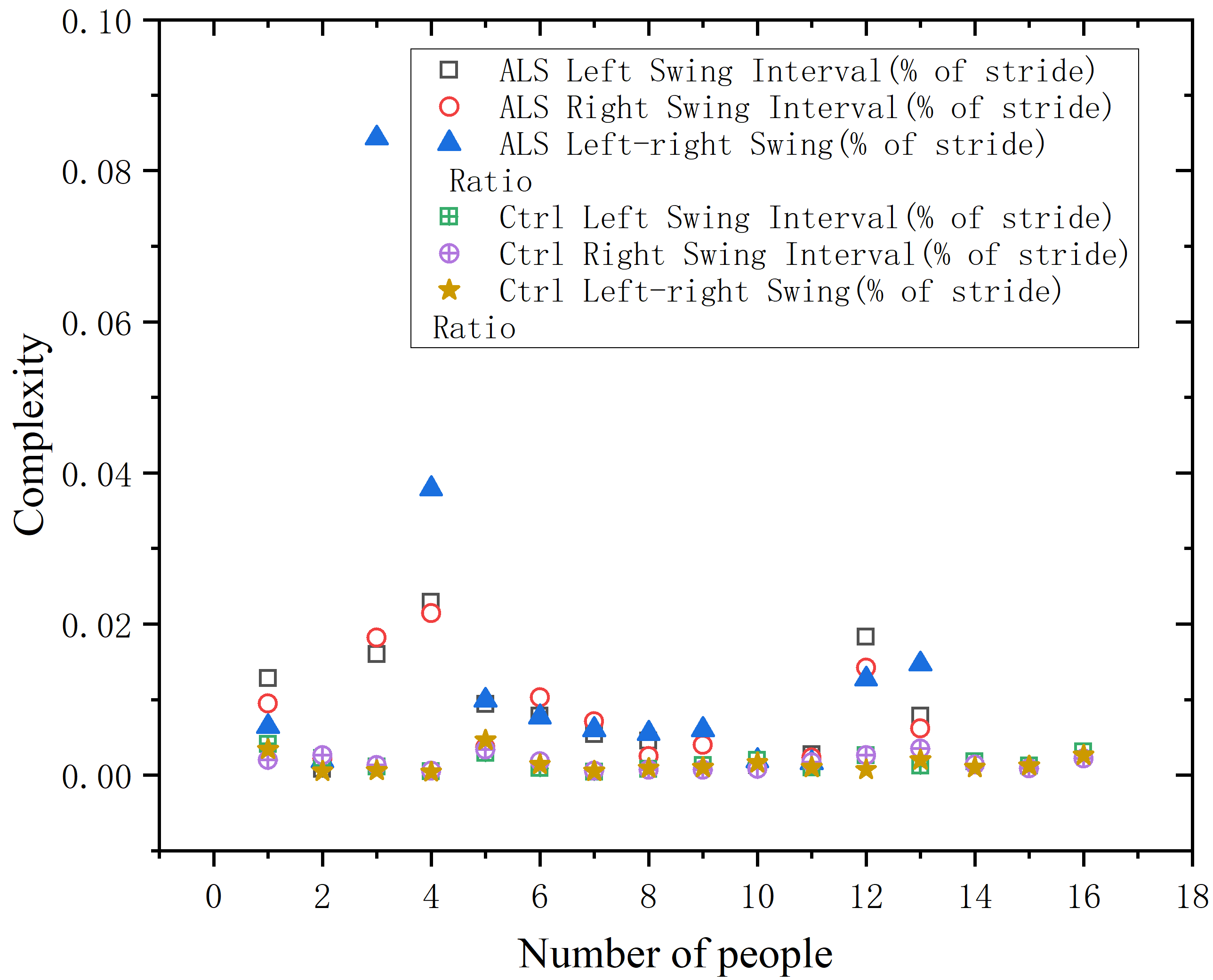

Supplement: Supplementary file 2 [file Data_Sheet_2.zip › Data Sheet 1/2c.png]

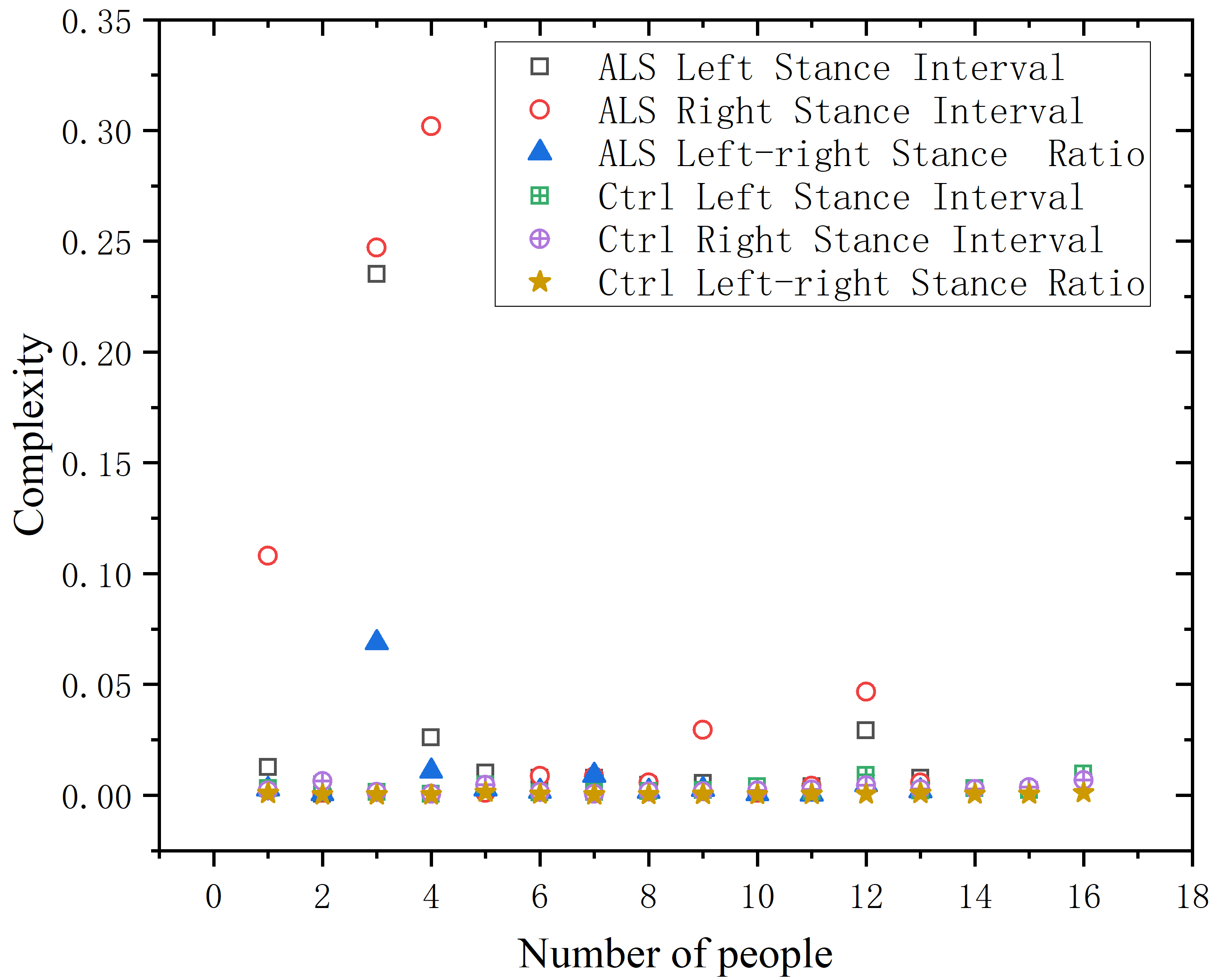

Supplement: Supplementary file 2 [file Data_Sheet_2.zip › Data Sheet 1/2d.png]

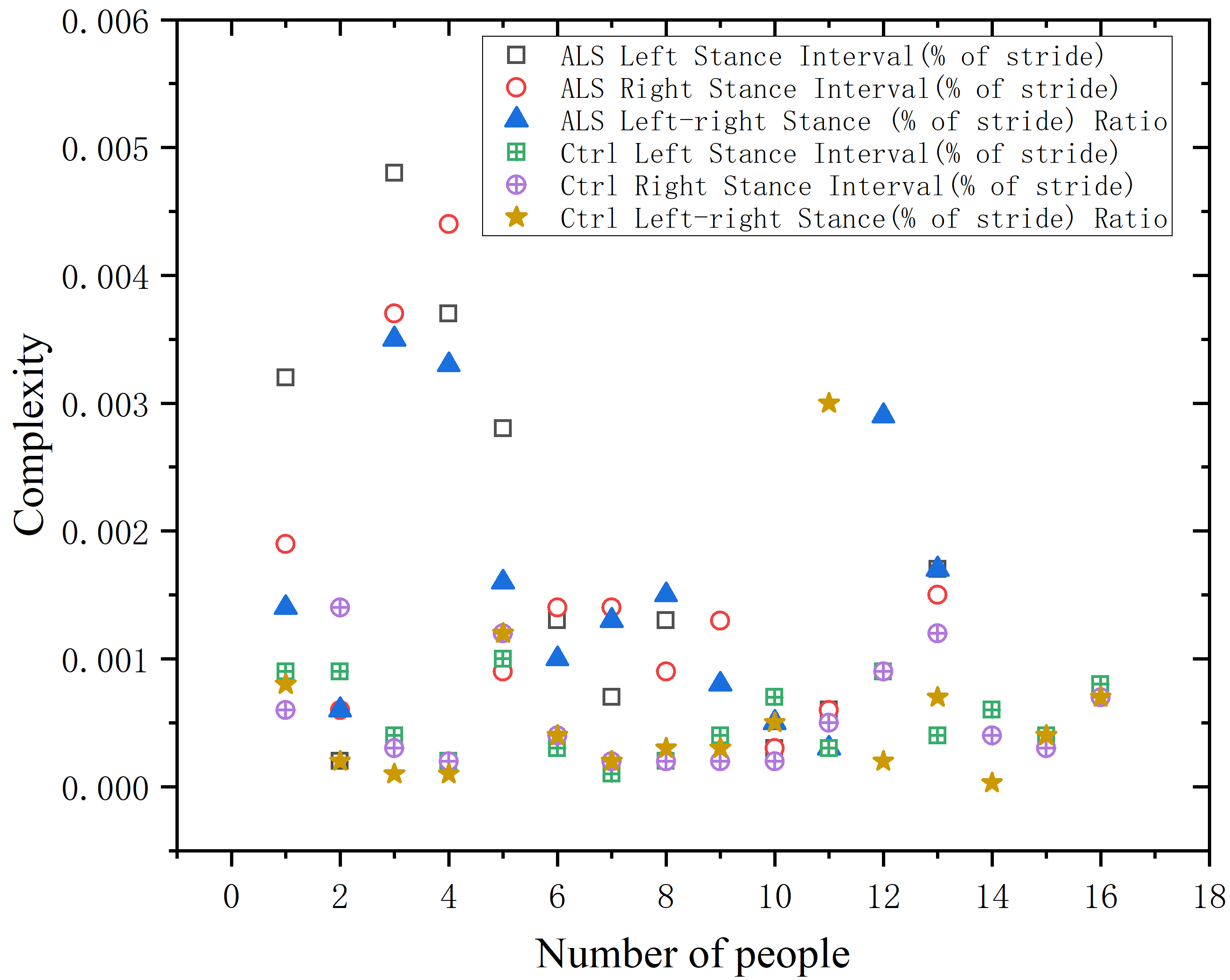

Supplement: Supplementary file 2 [file Data_Sheet_2.zip › Data Sheet 1/2e.png]

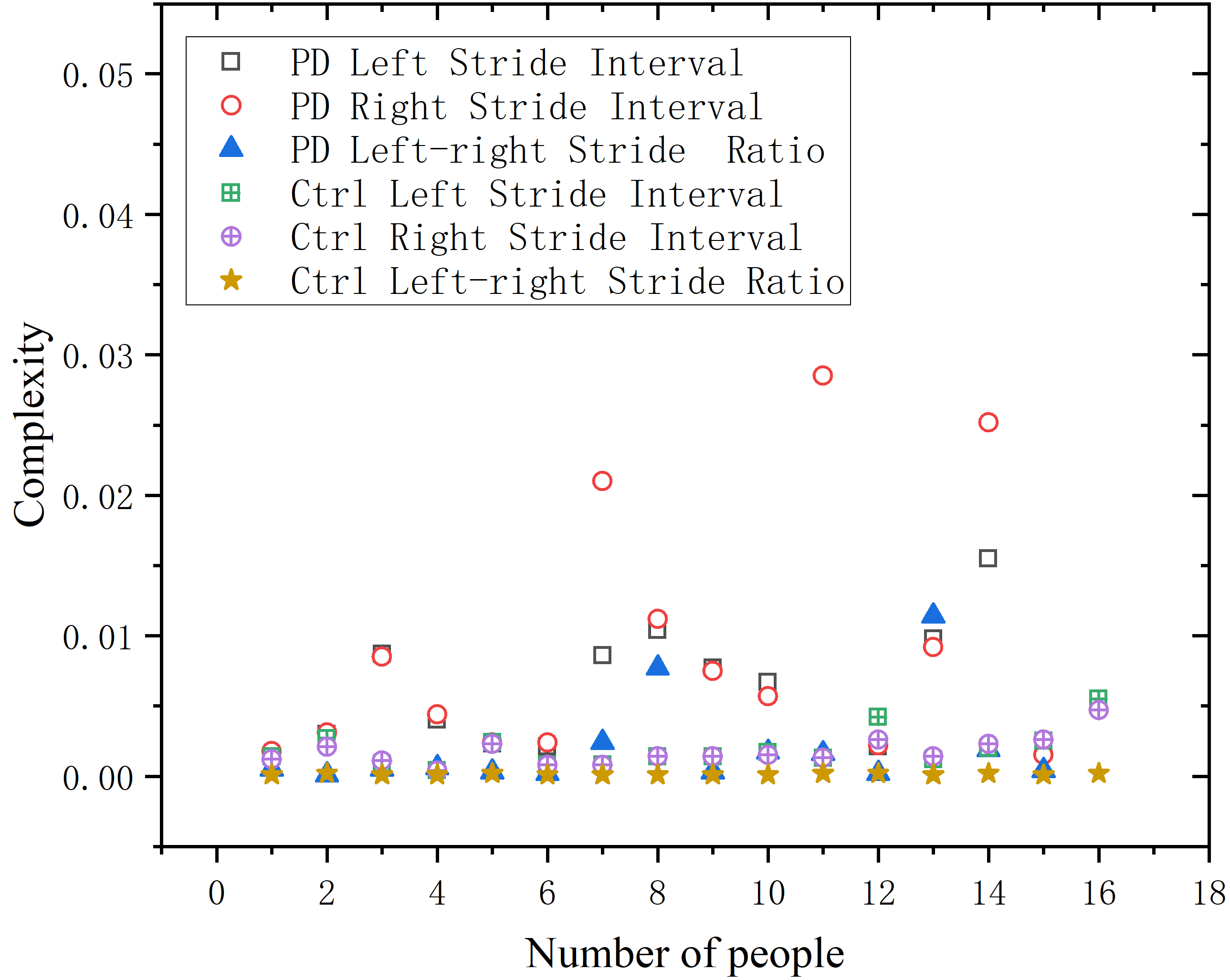

Supplement: Supplementary file 2 [file Data_Sheet_2.zip › Data Sheet 1/3a.png]

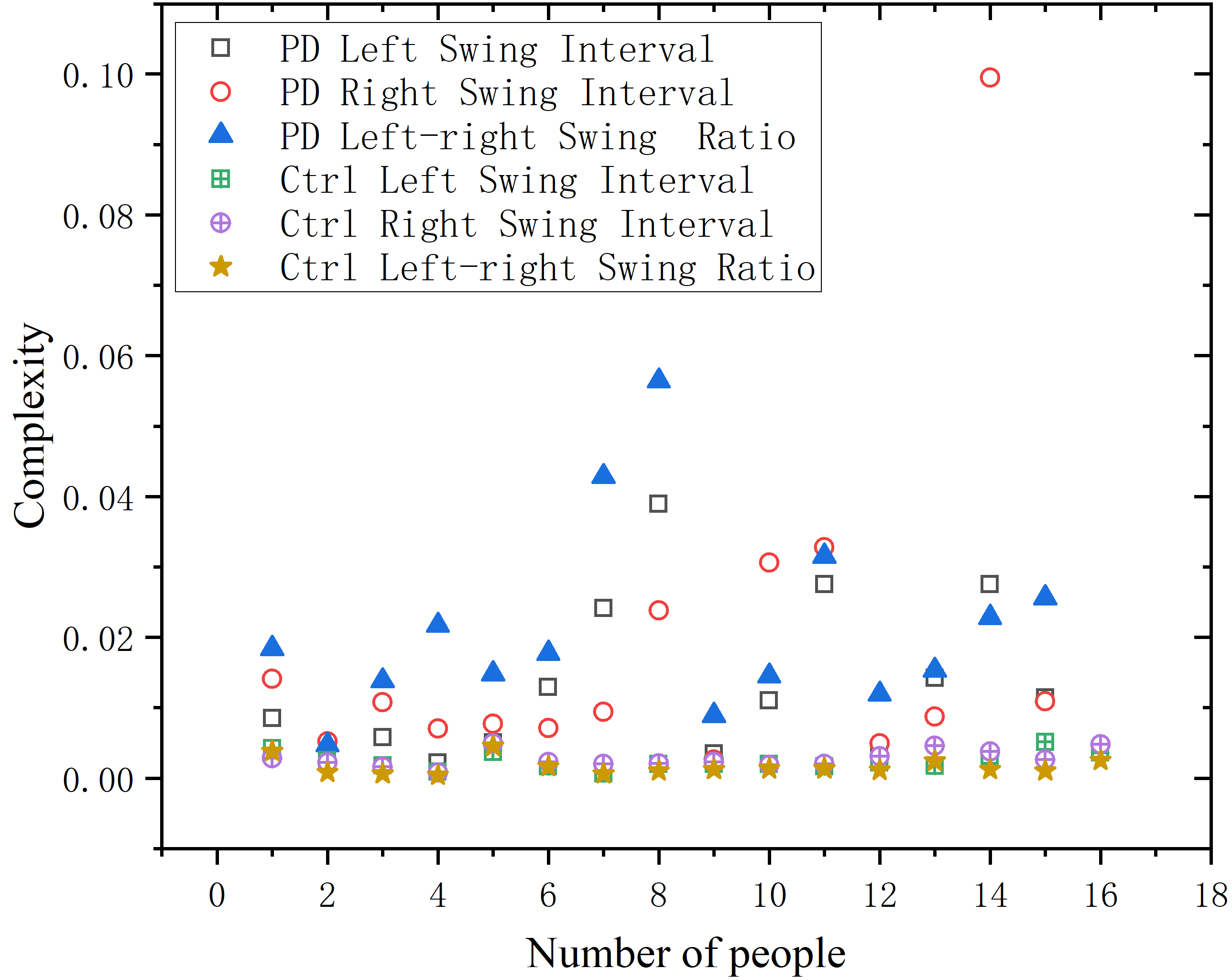

Supplement: Supplementary file 2 [file Data_Sheet_2.zip › Data Sheet 1/3b.png]

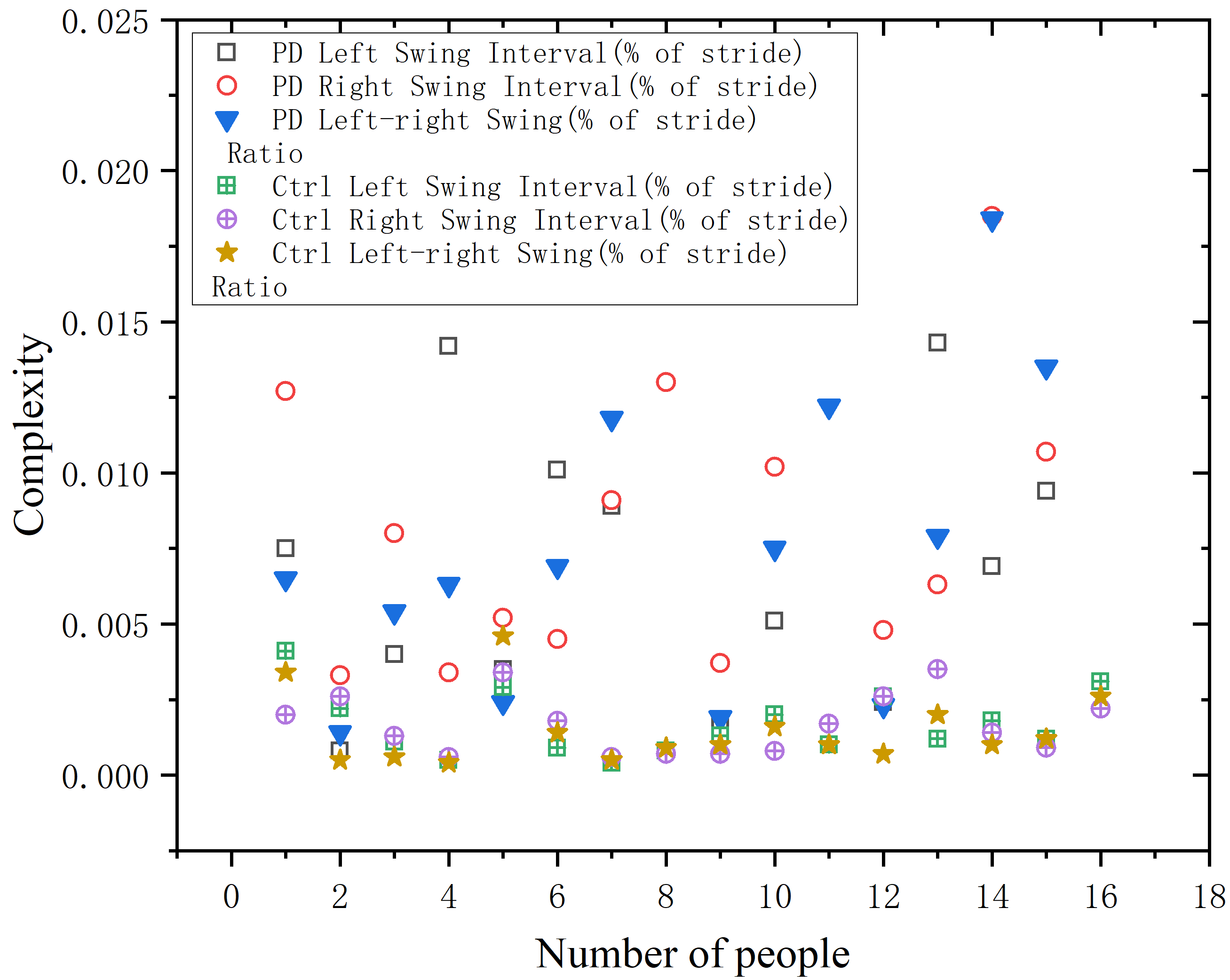

Supplement: Supplementary file 2 [file Data_Sheet_2.zip › Data Sheet 1/3c.png]

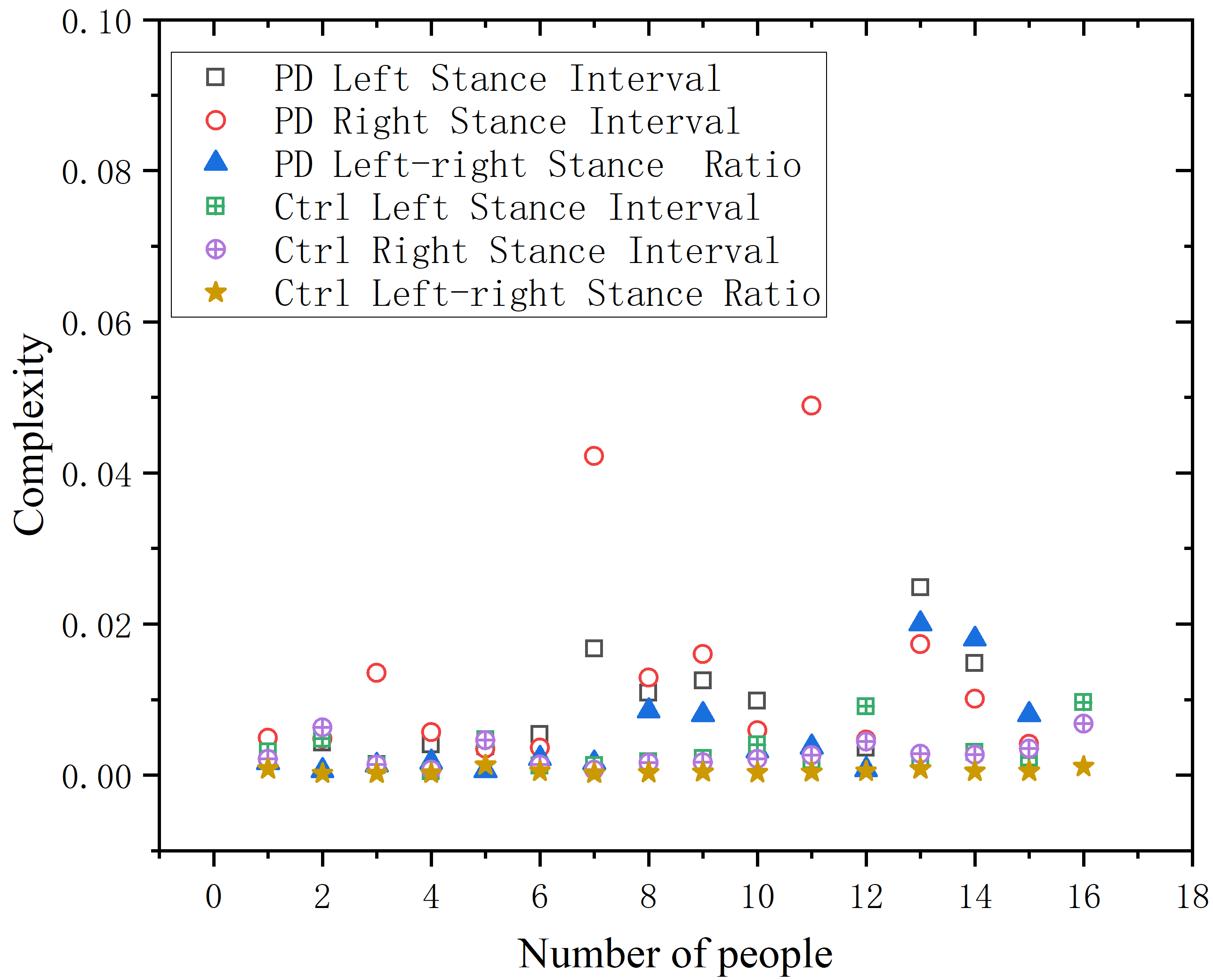

Supplement: Supplementary file 2 [file Data_Sheet_2.zip › Data Sheet 1/3d.png]

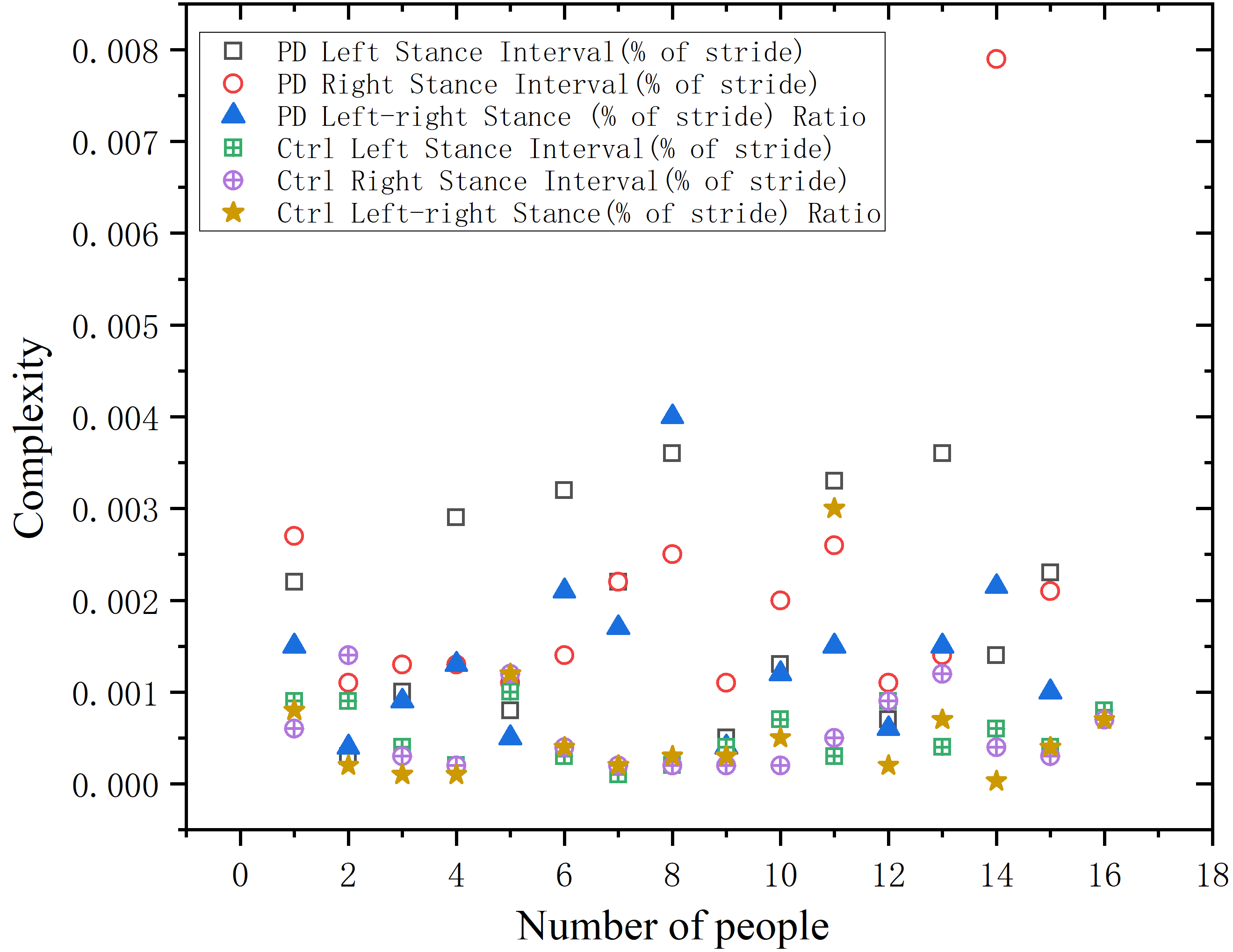

Supplement: Supplementary file 2 [file Data_Sheet_2.zip › Data Sheet 1/3e.png]

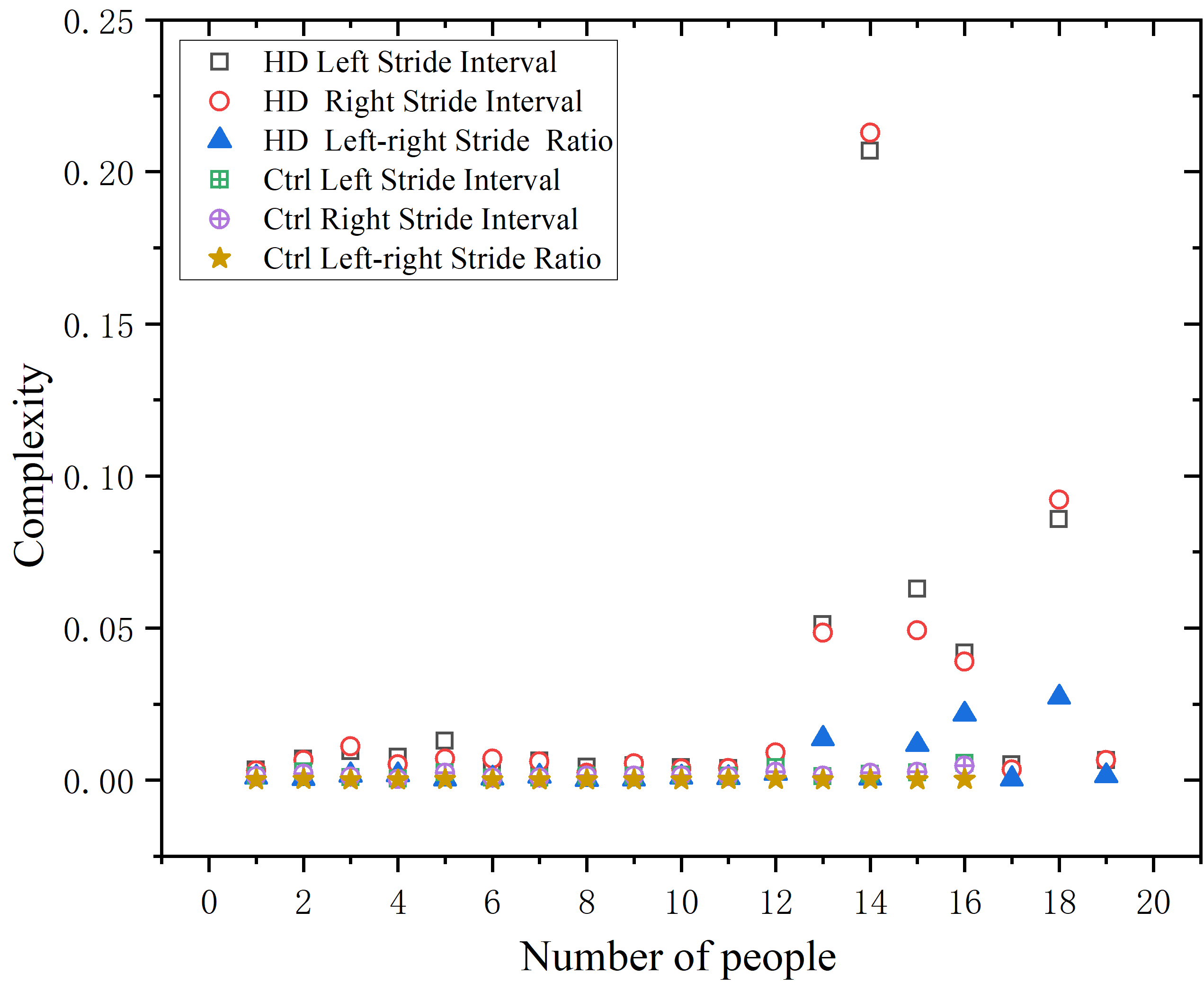

Supplement: Supplementary file 2 [file Data_Sheet_2.zip › Data Sheet 1/4a.png]

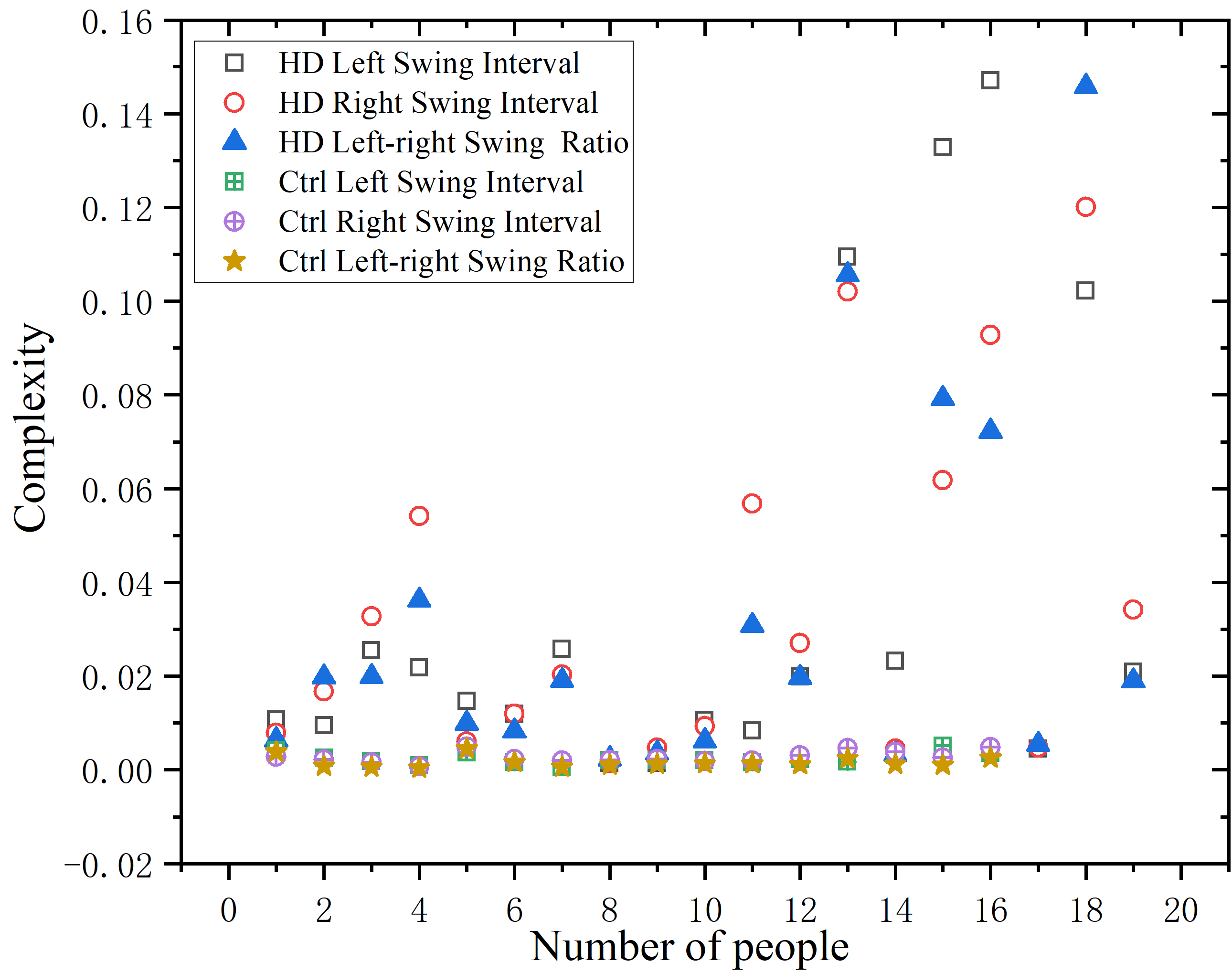

Supplement: Supplementary file 2 [file Data_Sheet_2.zip › Data Sheet 1/4b.png]

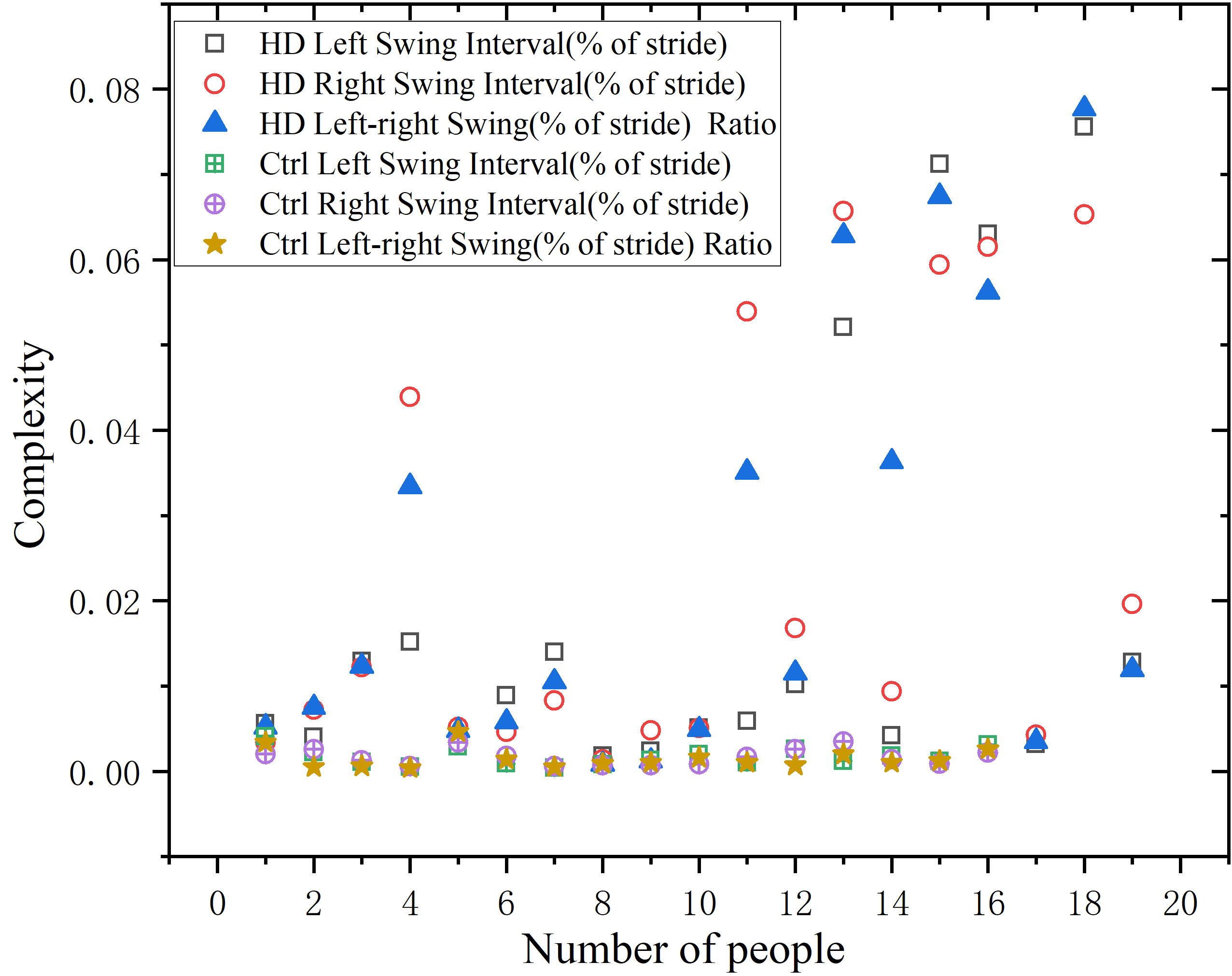

Supplement: Supplementary file 2 [file Data_Sheet_2.zip › Data Sheet 1/4c.png]

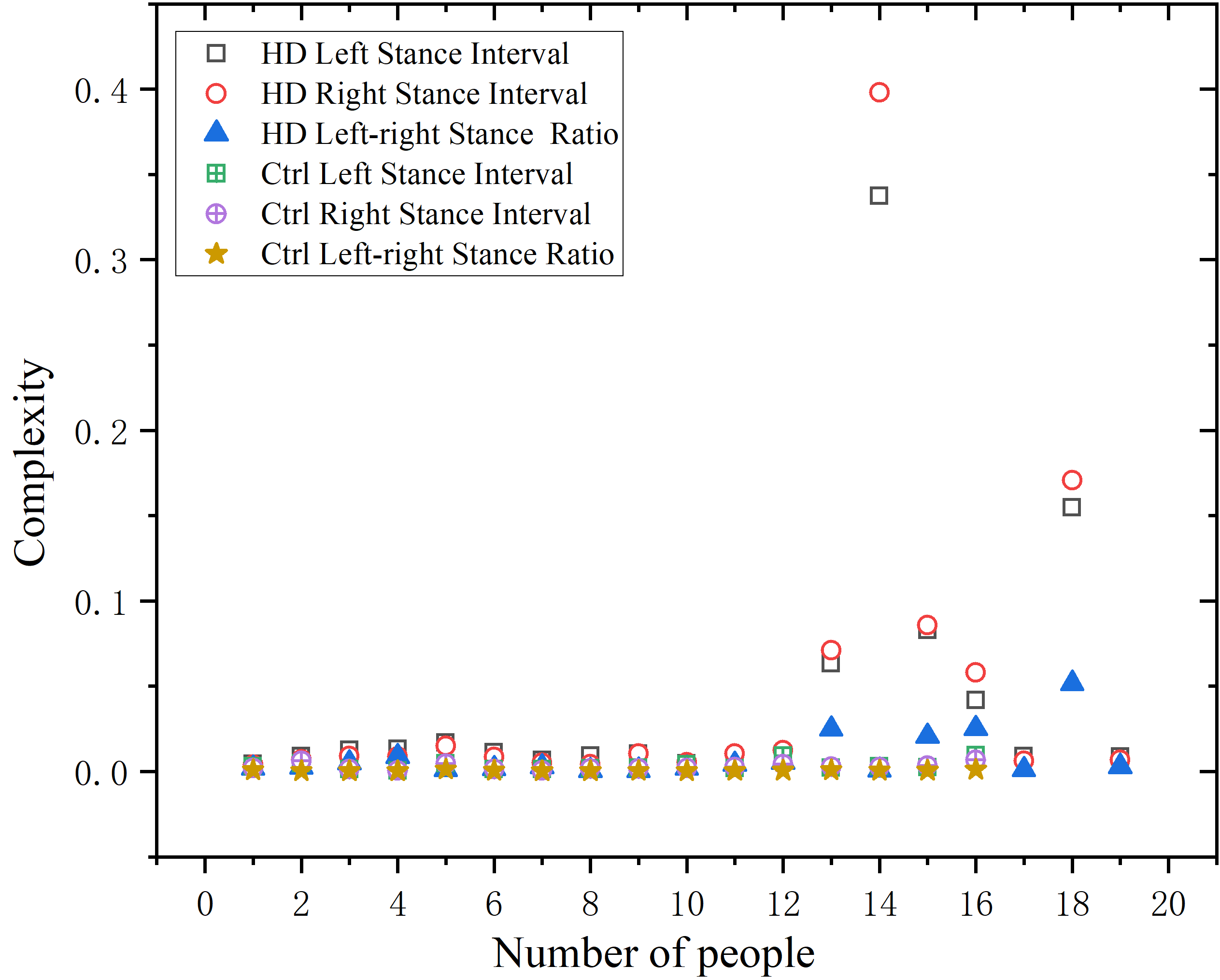

Supplement: Supplementary file 2 [file Data_Sheet_2.zip › Data Sheet 1/4d.png]

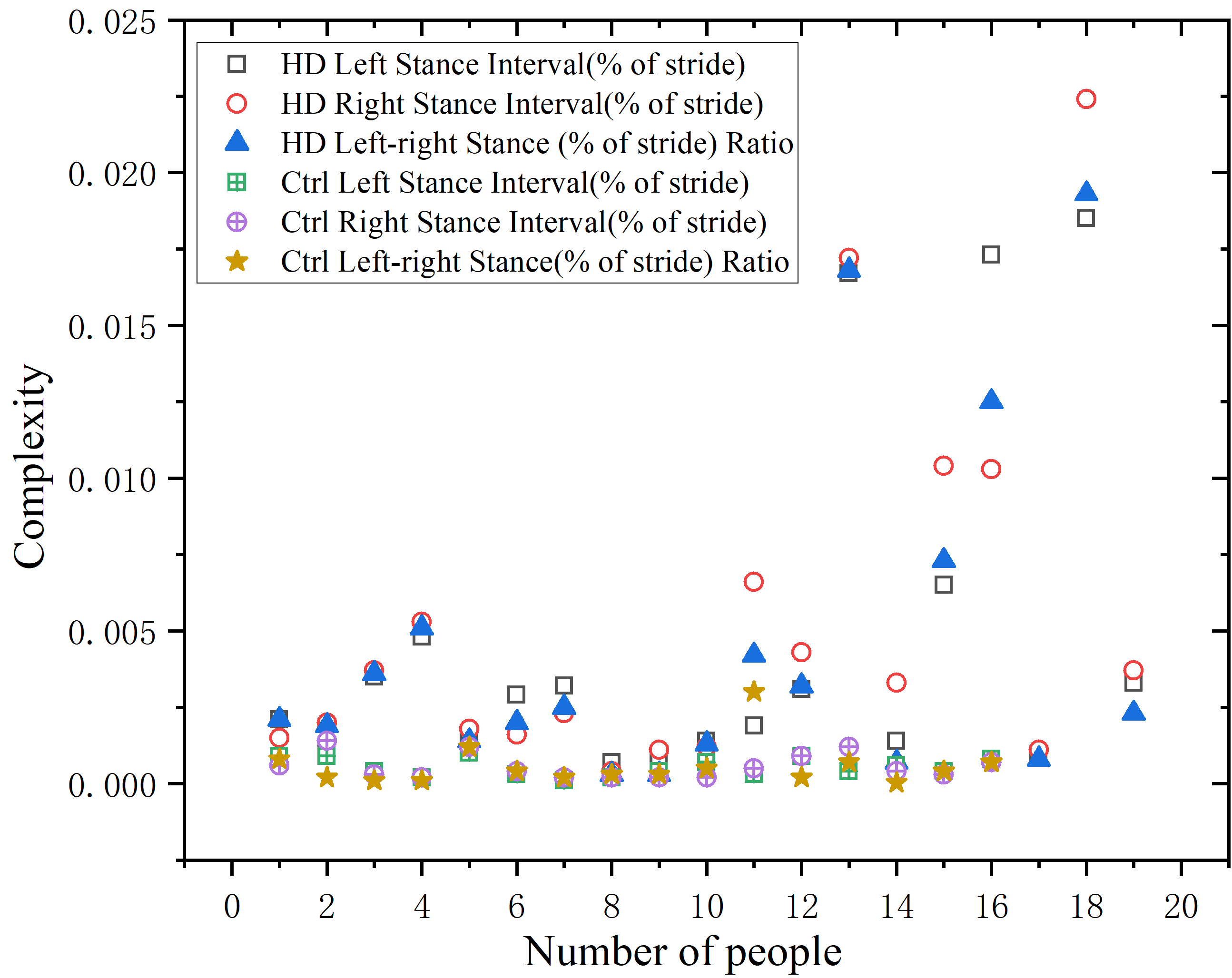

Supplement: Supplementary file 2 [file Data_Sheet_2.zip › Data Sheet 1/4e.png]

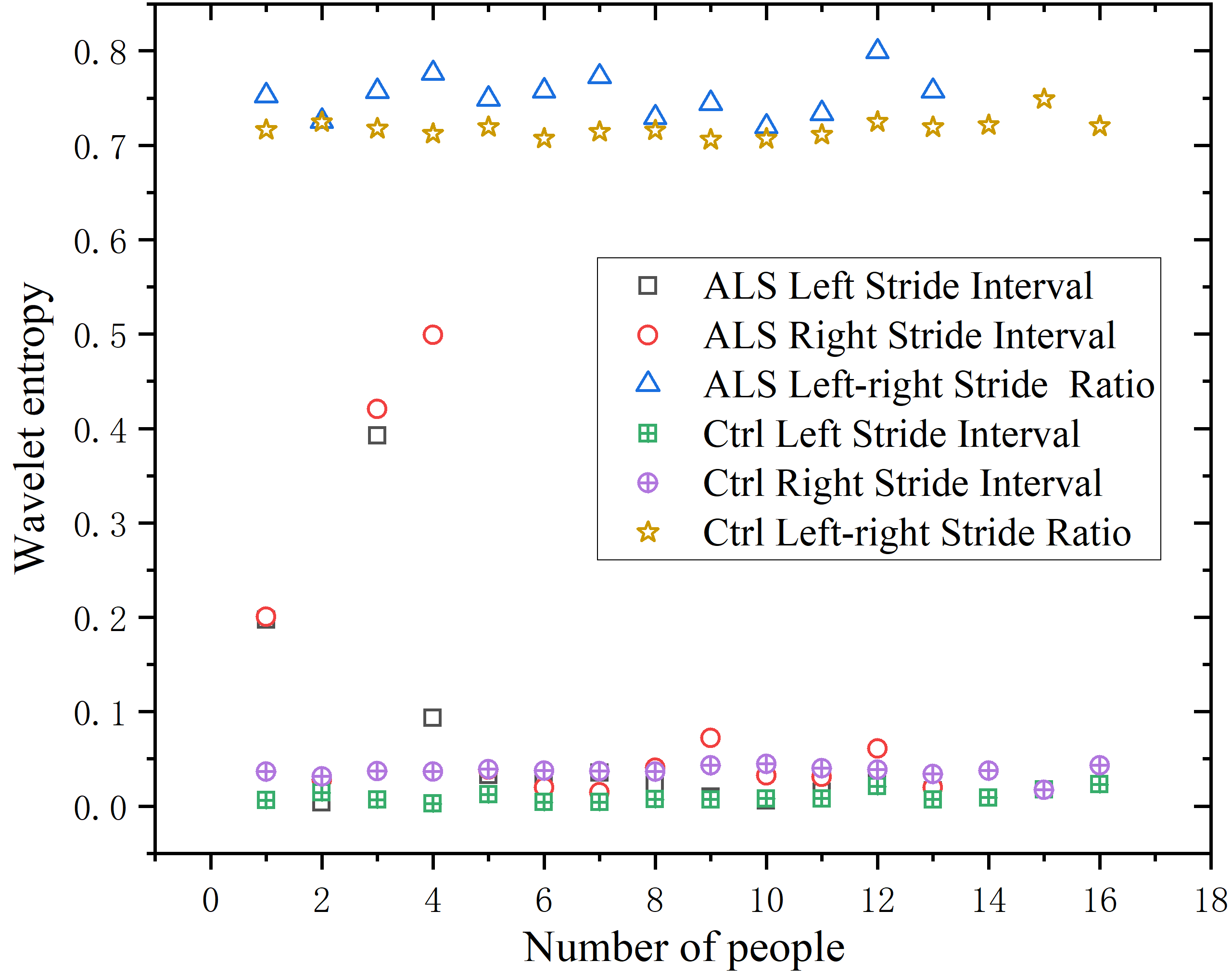

Supplement: Supplementary file 2 [file Data_Sheet_2.zip › Data Sheet 1/5a.png]

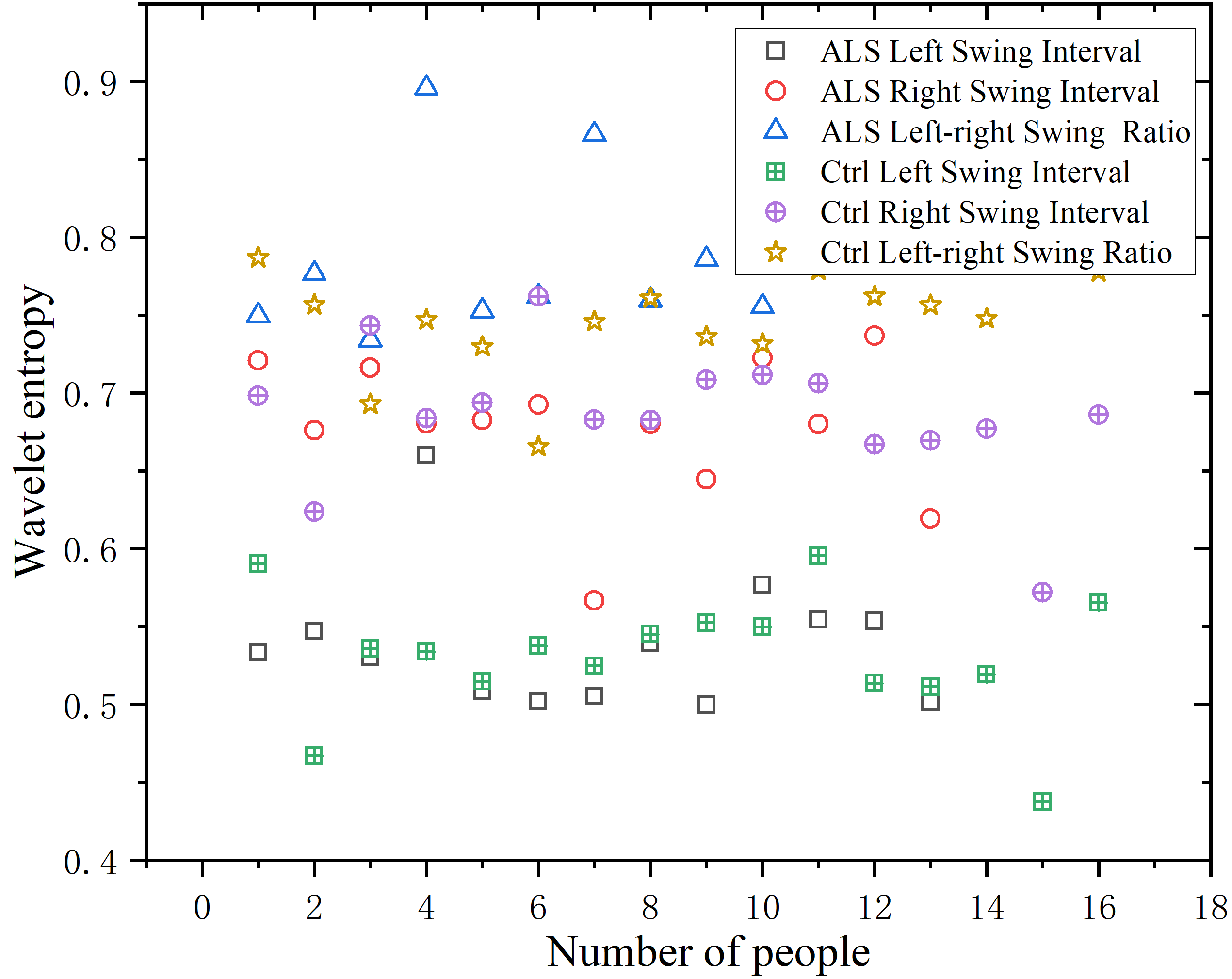

Supplement: Supplementary file 2 [file Data_Sheet_2.zip › Data Sheet 1/5b.png]

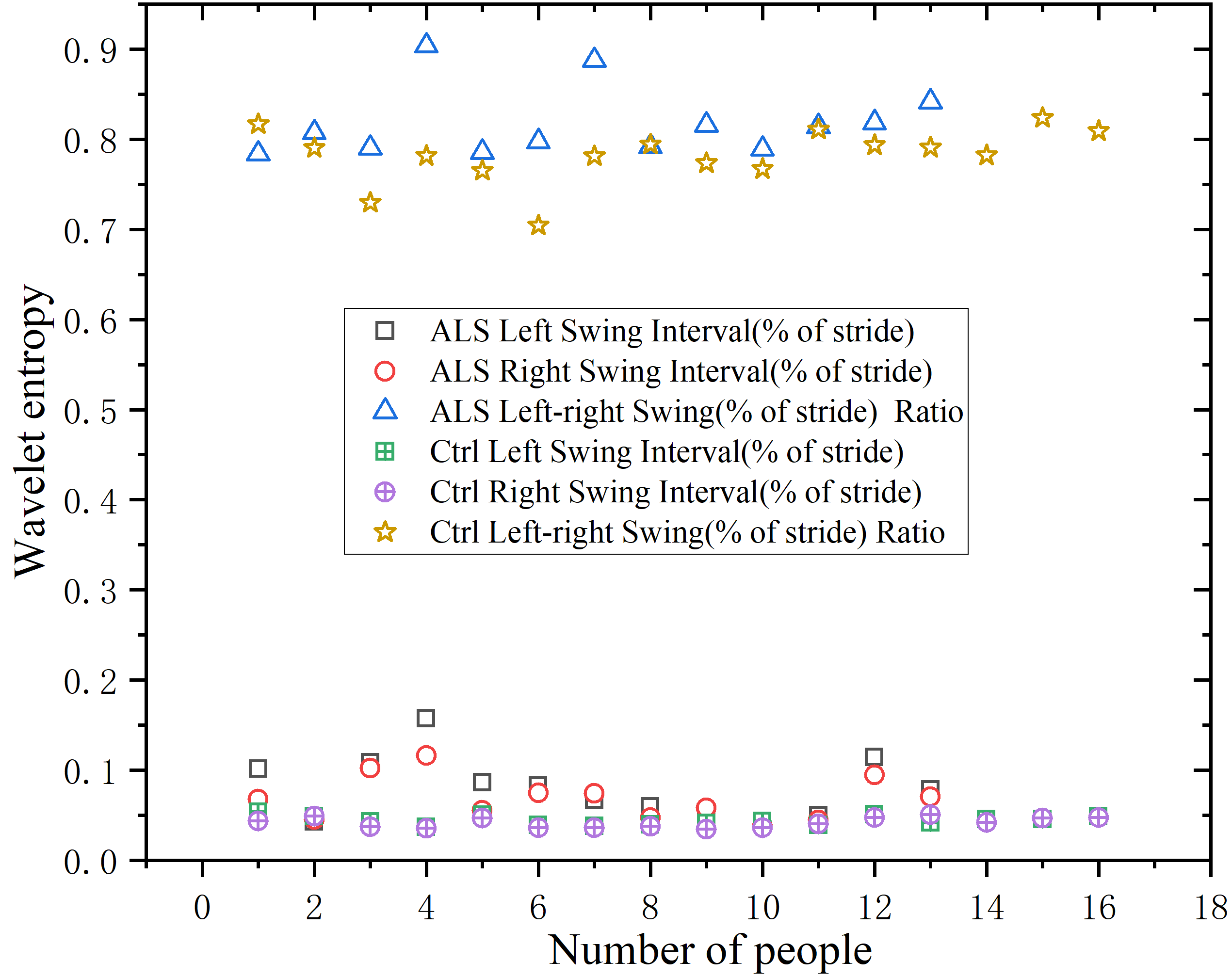

Supplement: Supplementary file 2 [file Data_Sheet_2.zip › Data Sheet 1/5c.png]

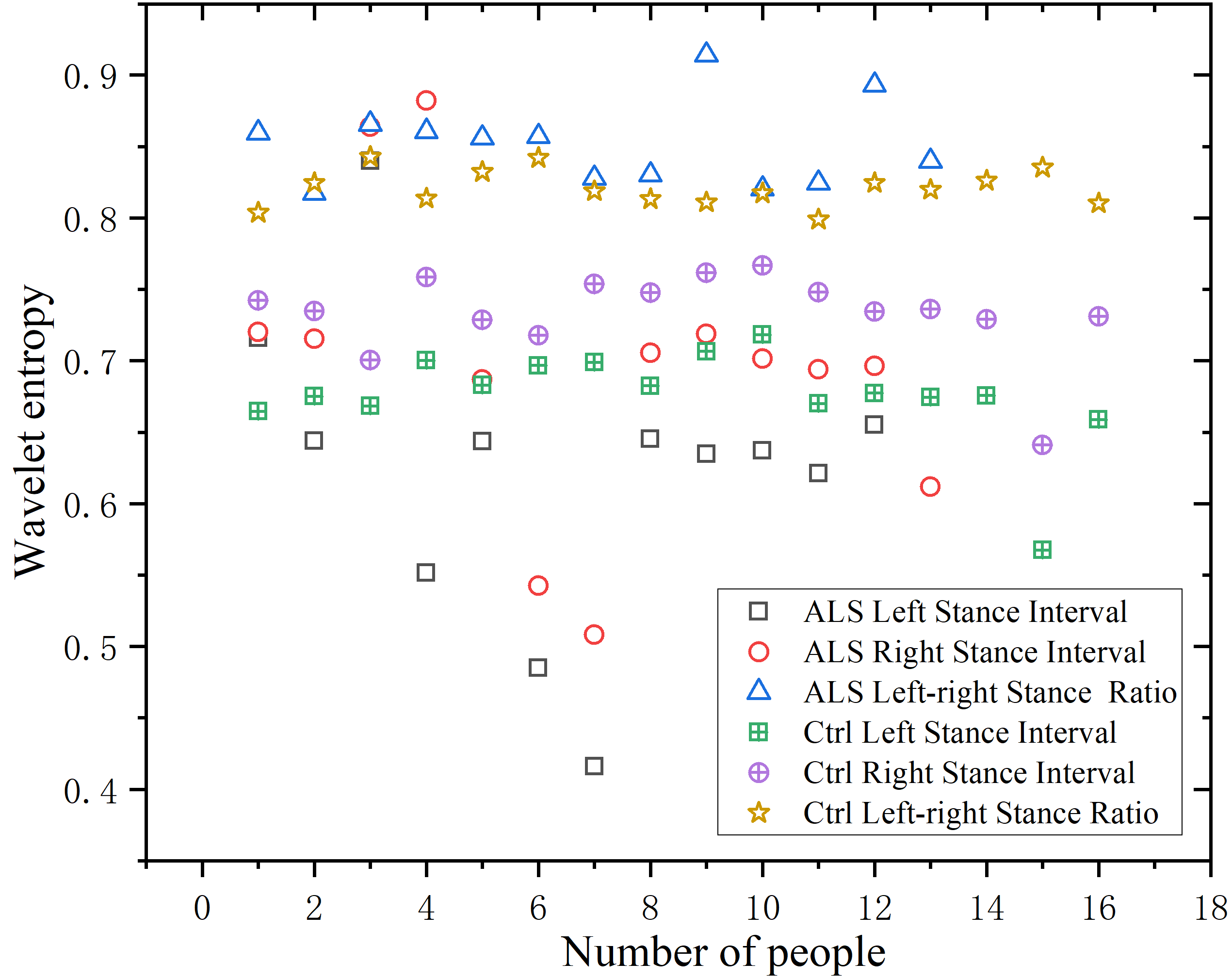

Supplement: Supplementary file 2 [file Data_Sheet_2.zip › Data Sheet 1/5d.png]

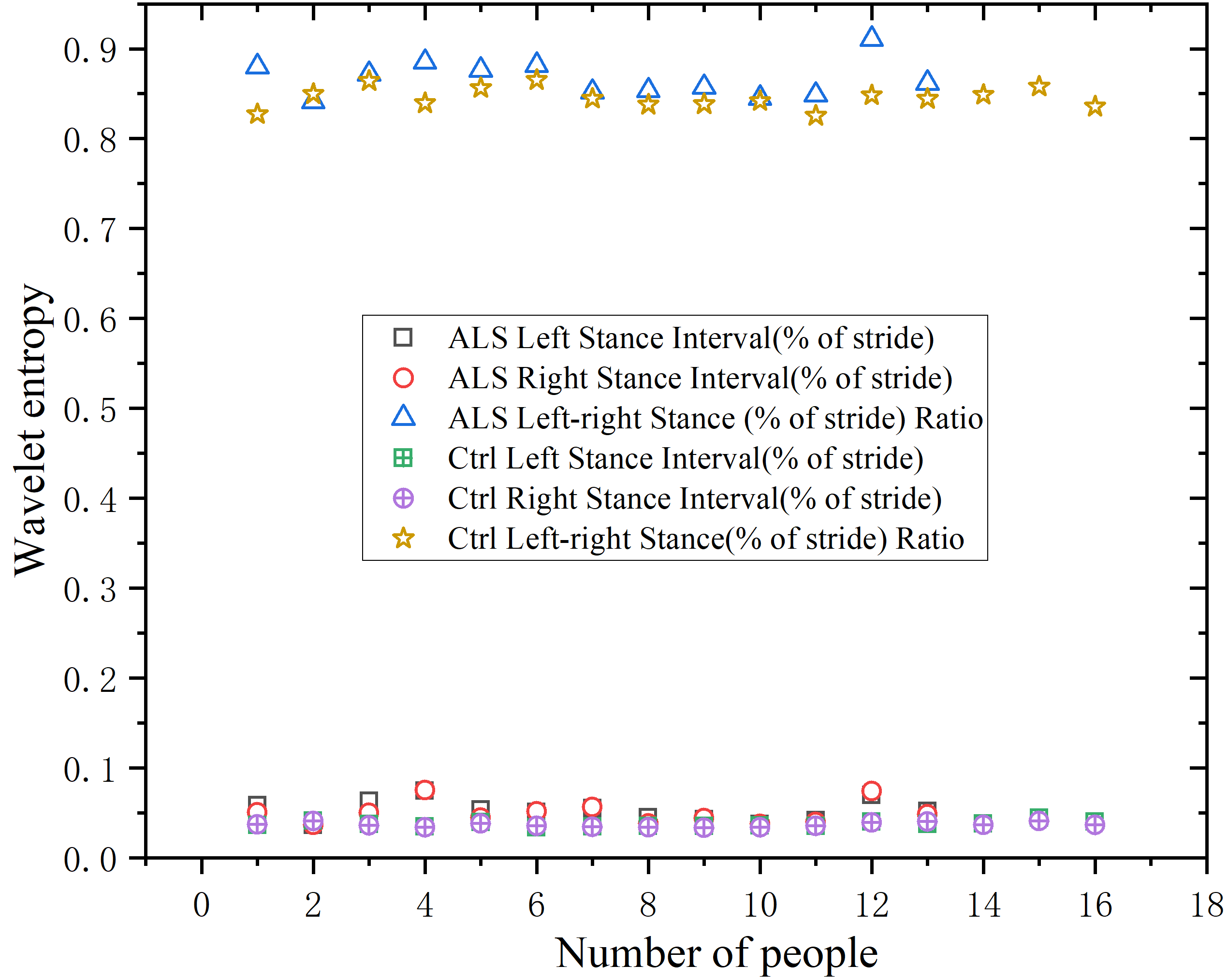

Supplement: Supplementary file 2 [file Data_Sheet_2.zip › Data Sheet 1/5e.png]

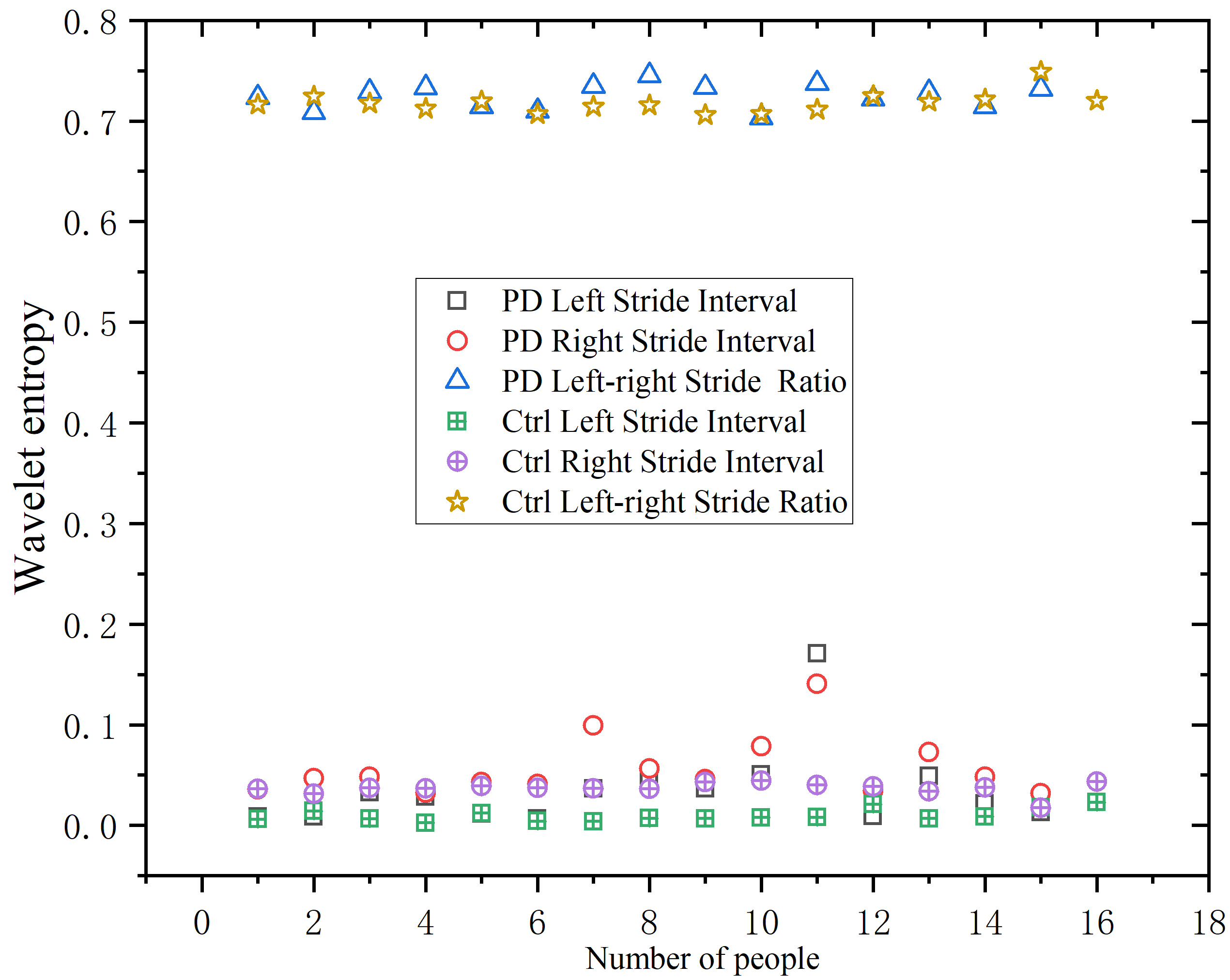

Supplement: Supplementary file 2 [file Data_Sheet_2.zip › Data Sheet 1/6a.png]

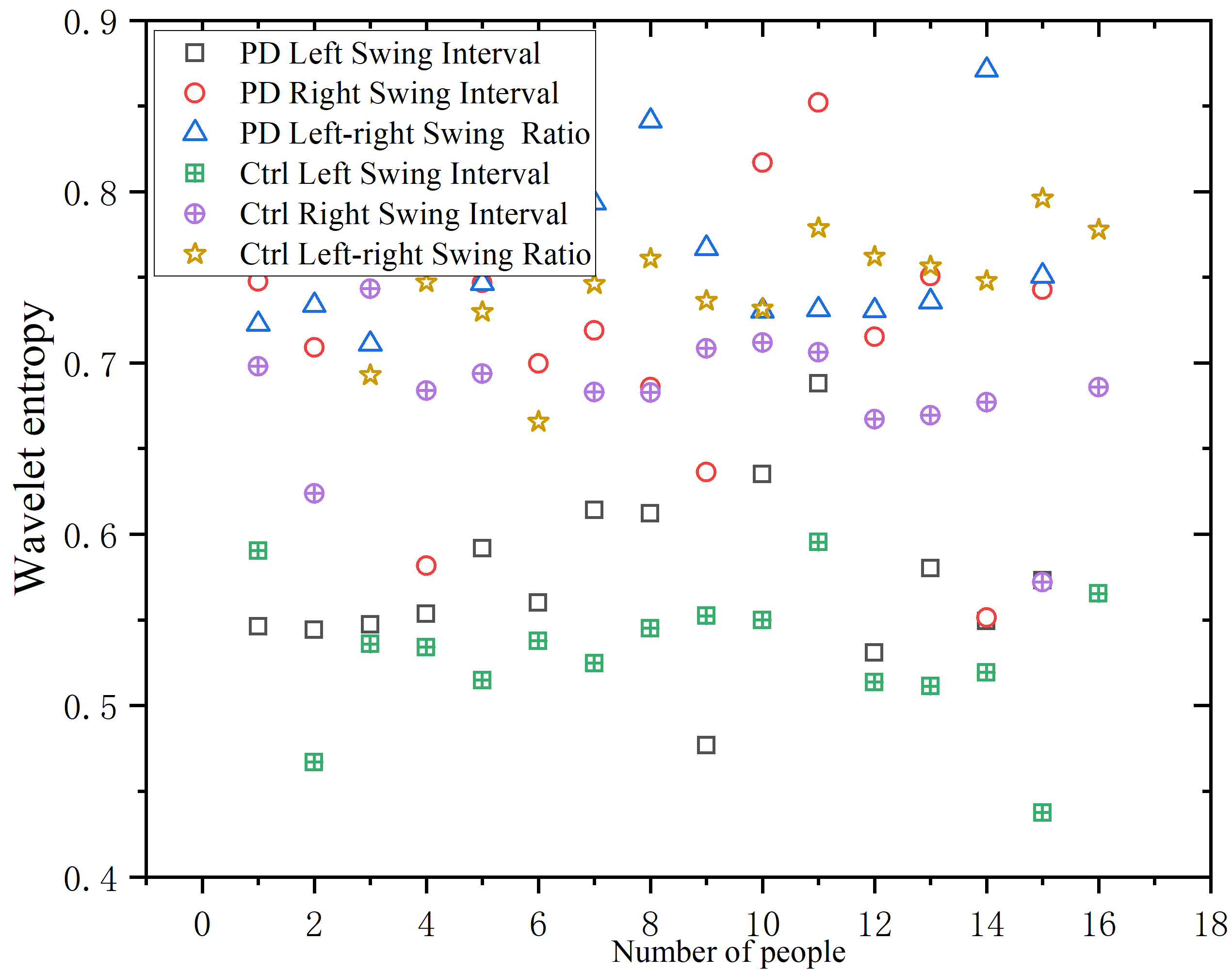

Supplement: Supplementary file 2 [file Data_Sheet_2.zip › Data Sheet 1/6b.png]

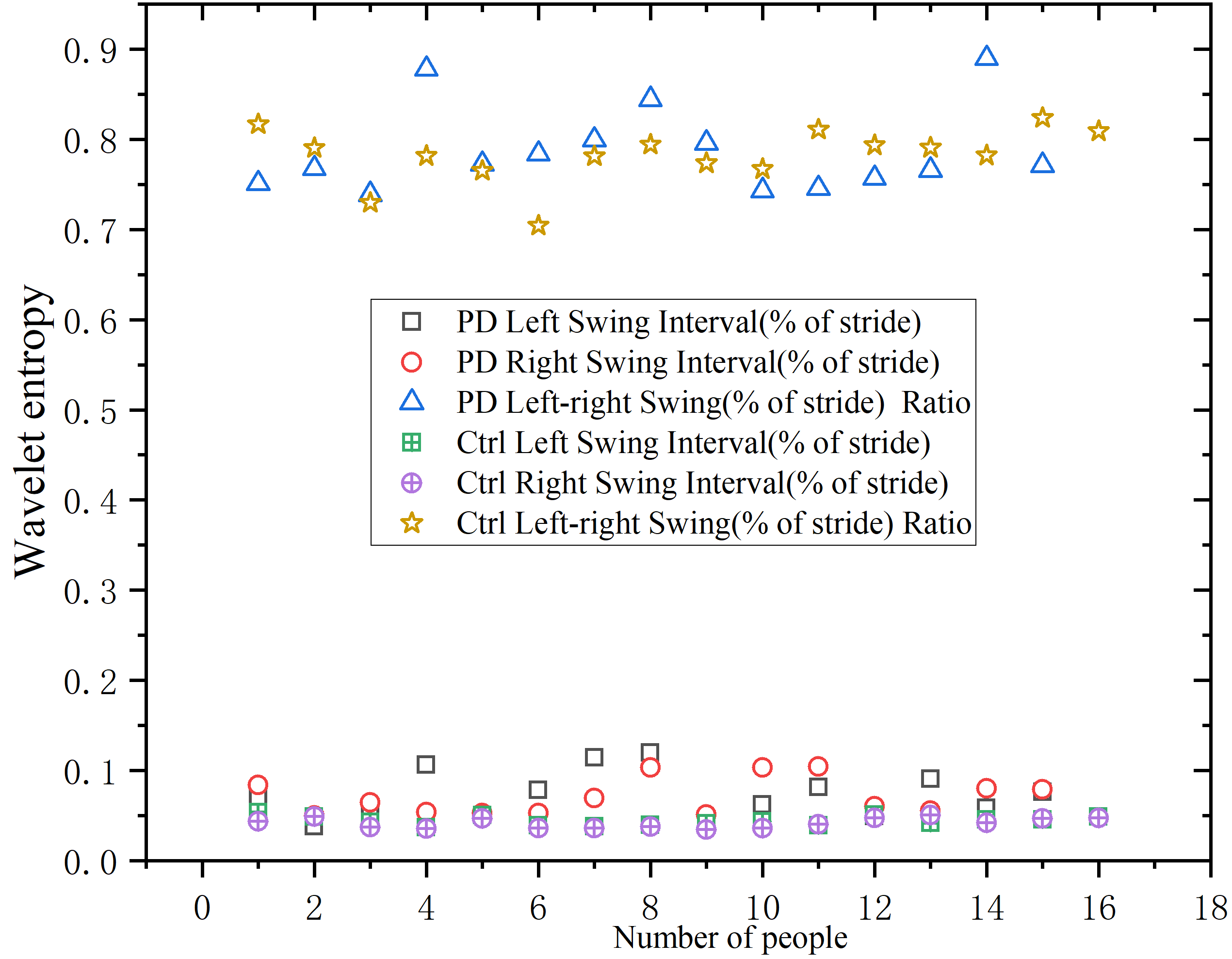

Supplement: Supplementary file 2 [file Data_Sheet_2.zip › Data Sheet 1/6c.png]

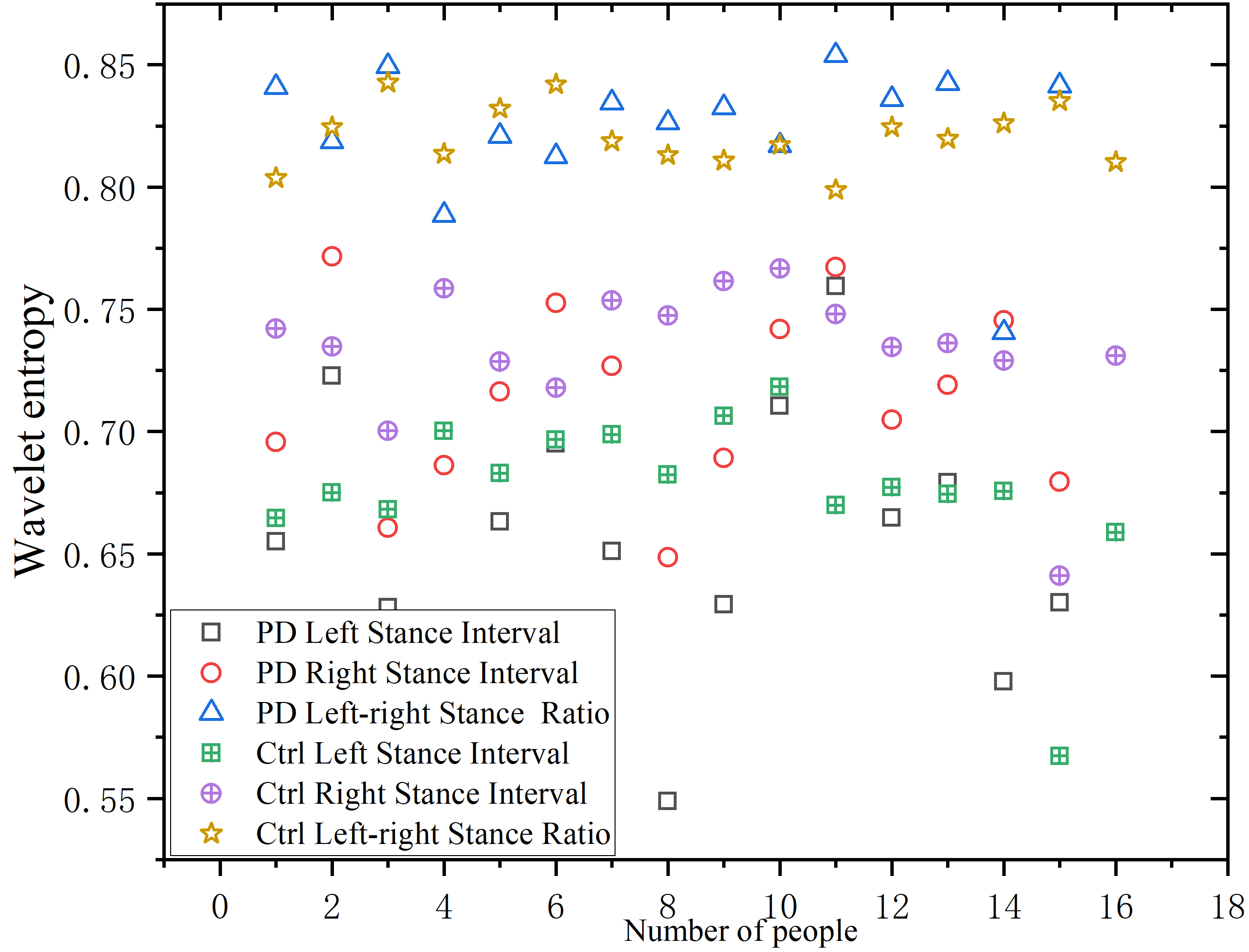

Supplement: Supplementary file 2 [file Data_Sheet_2.zip › Data Sheet 1/6d.png]

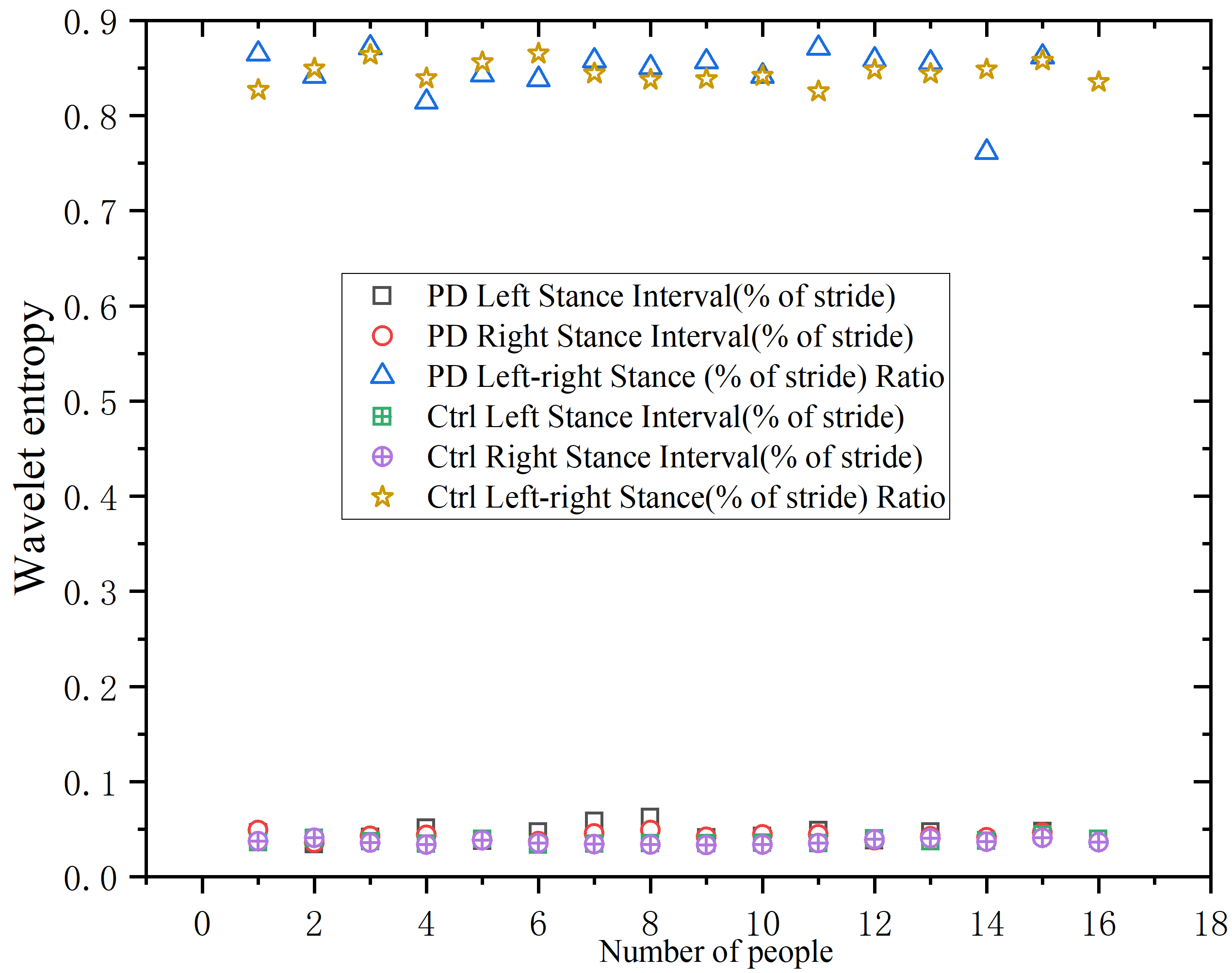

Supplement: Supplementary file 2 [file Data_Sheet_2.zip › Data Sheet 1/6e.png]

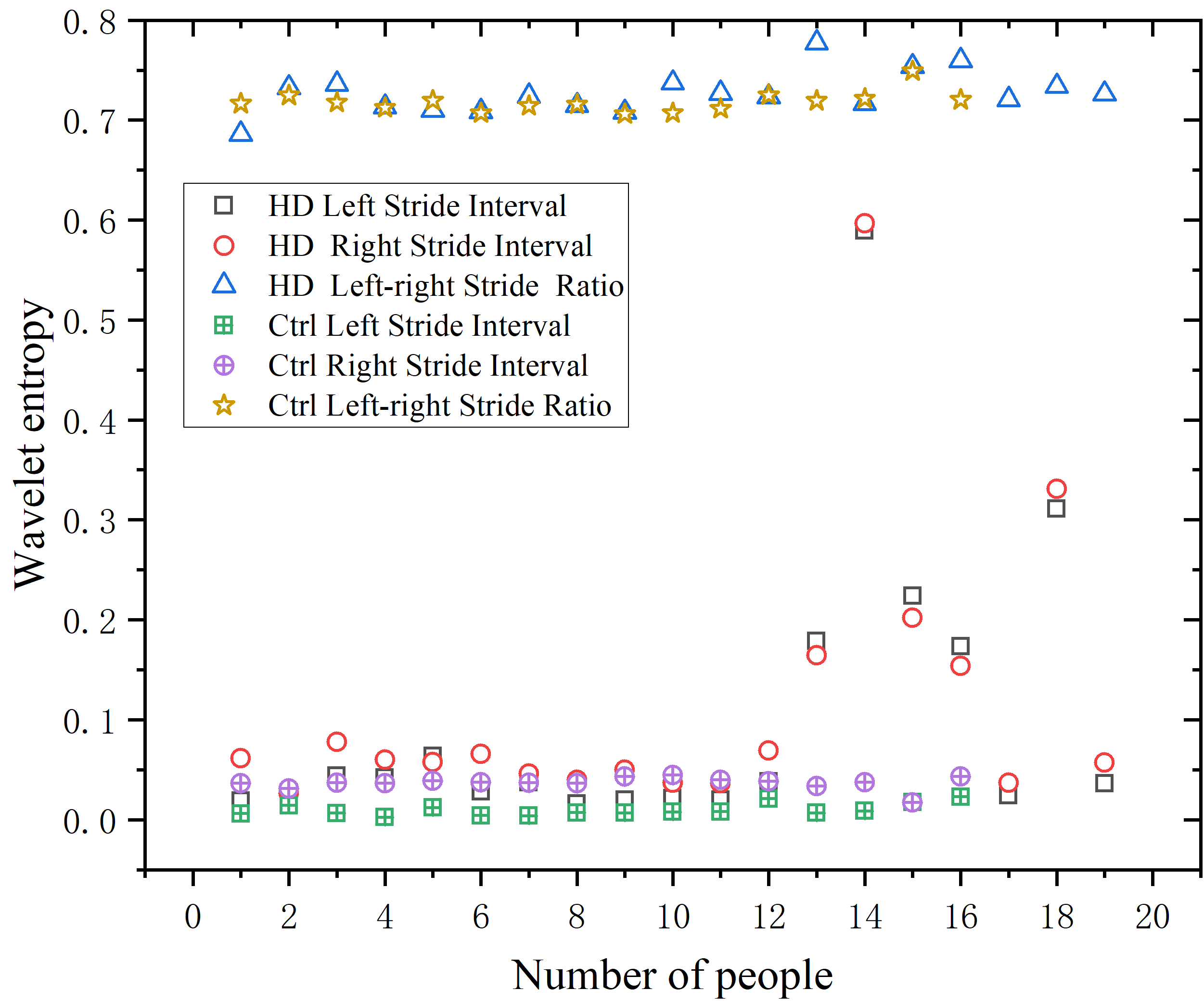

Supplement: Supplementary file 2 [file Data_Sheet_2.zip › Data Sheet 1/7a.png]

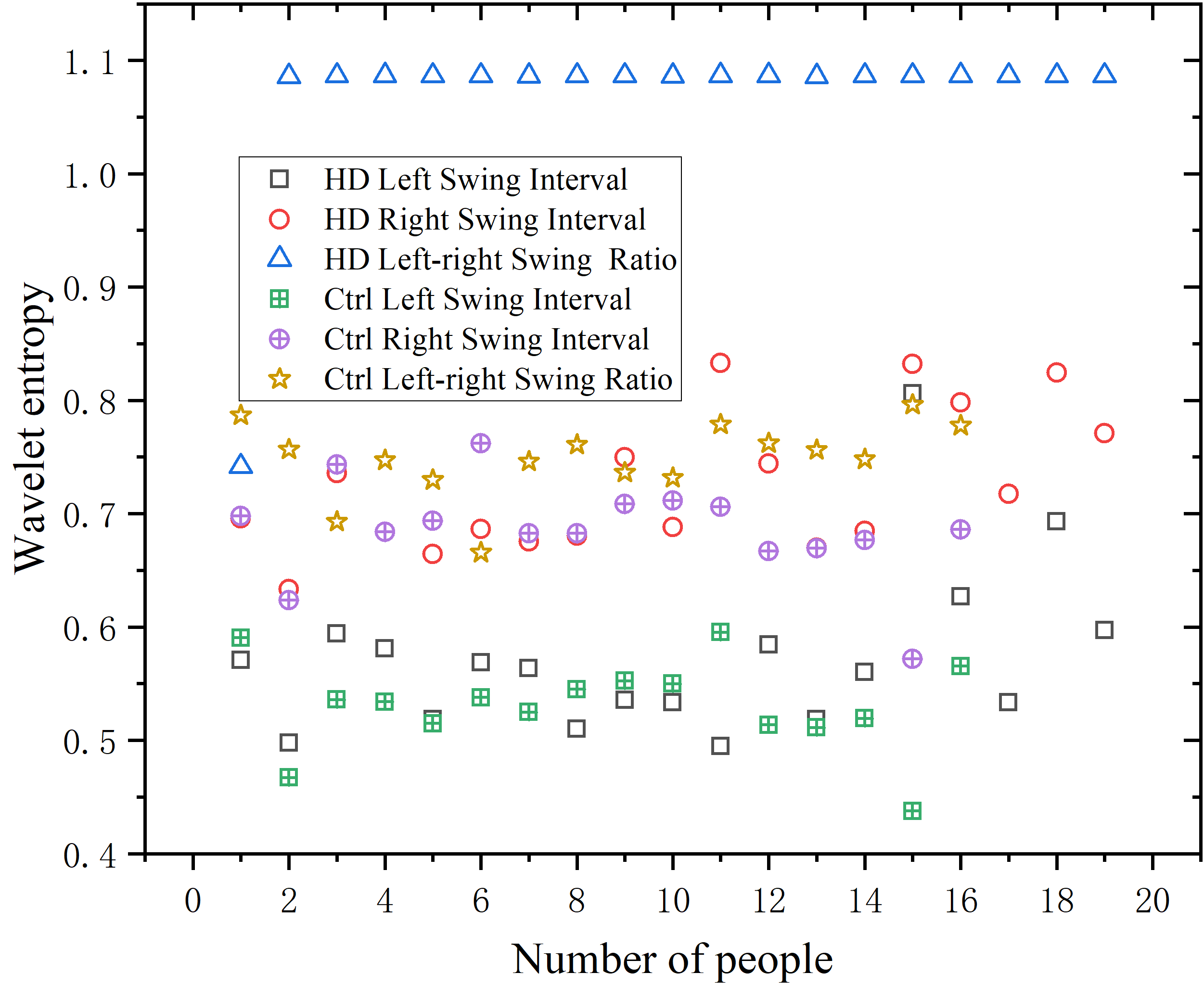

Supplement: Supplementary file 2 [file Data_Sheet_2.zip › Data Sheet 1/7b.png]

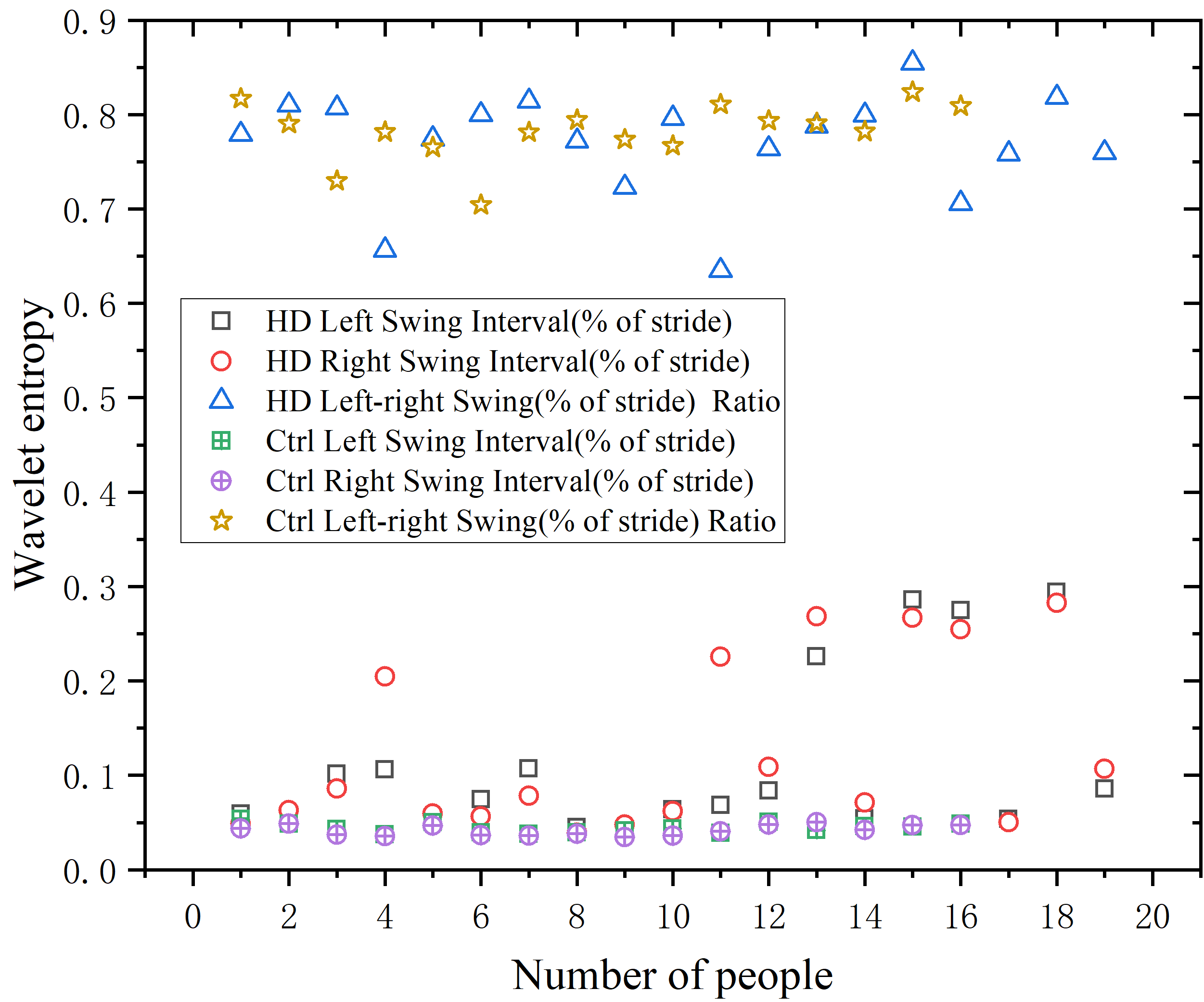

Supplement: Supplementary file 2 [file Data_Sheet_2.zip › Data Sheet 1/7c.png]

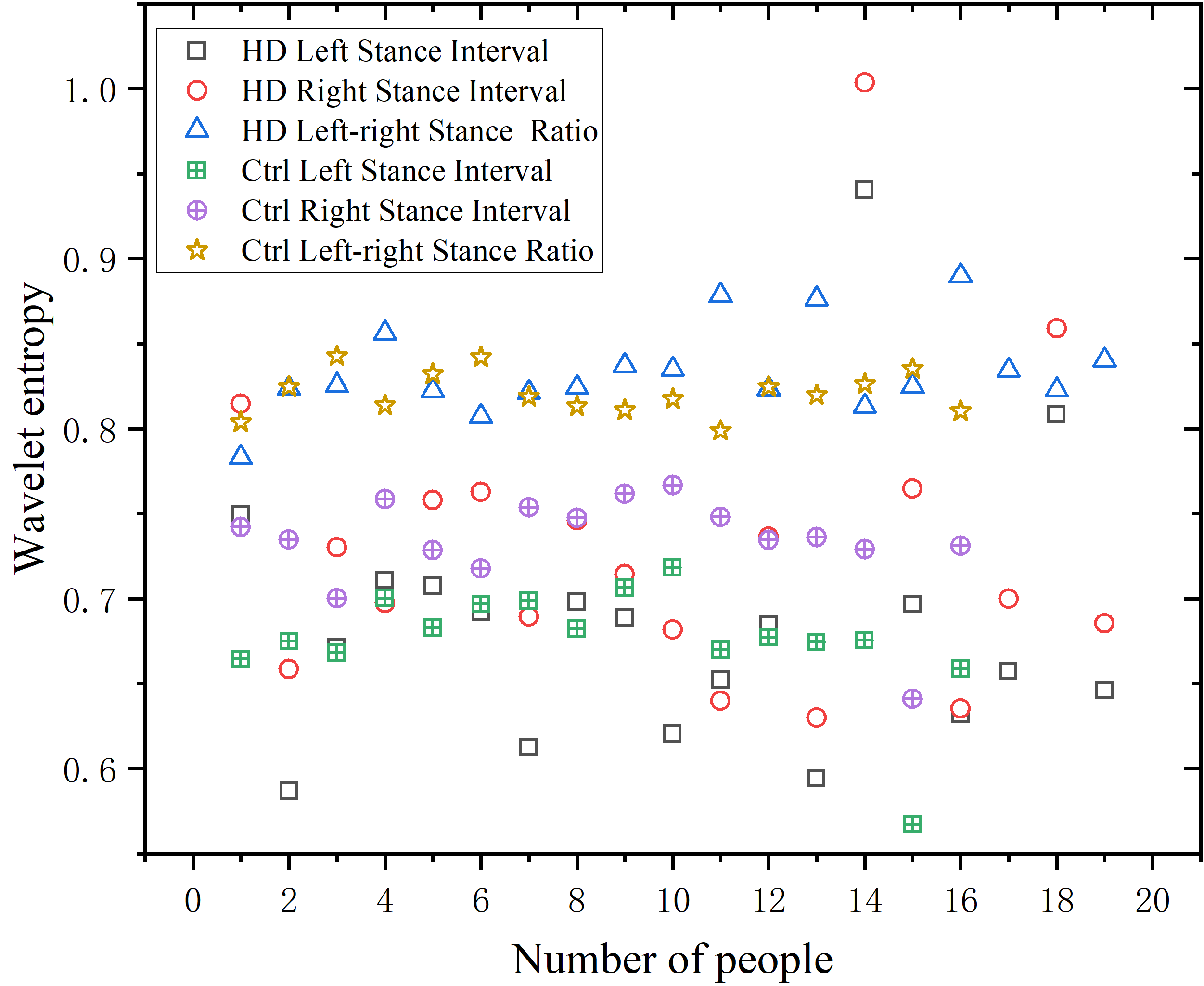

Supplement: Supplementary file 2 [file Data_Sheet_2.zip › Data Sheet 1/7d.png]

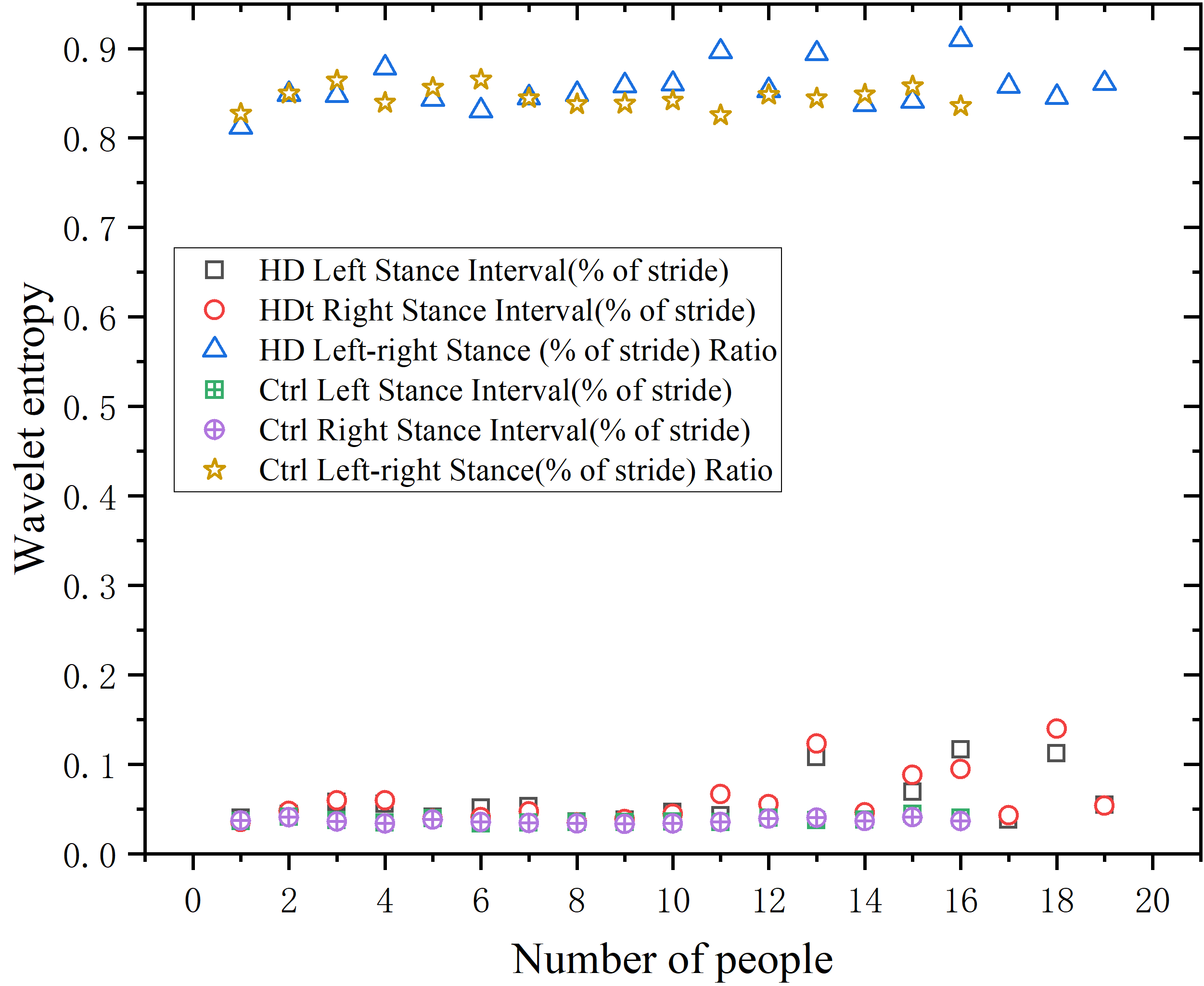

Supplement: Supplementary file 2 [file Data_Sheet_2.zip › Data Sheet 1/7e.png]

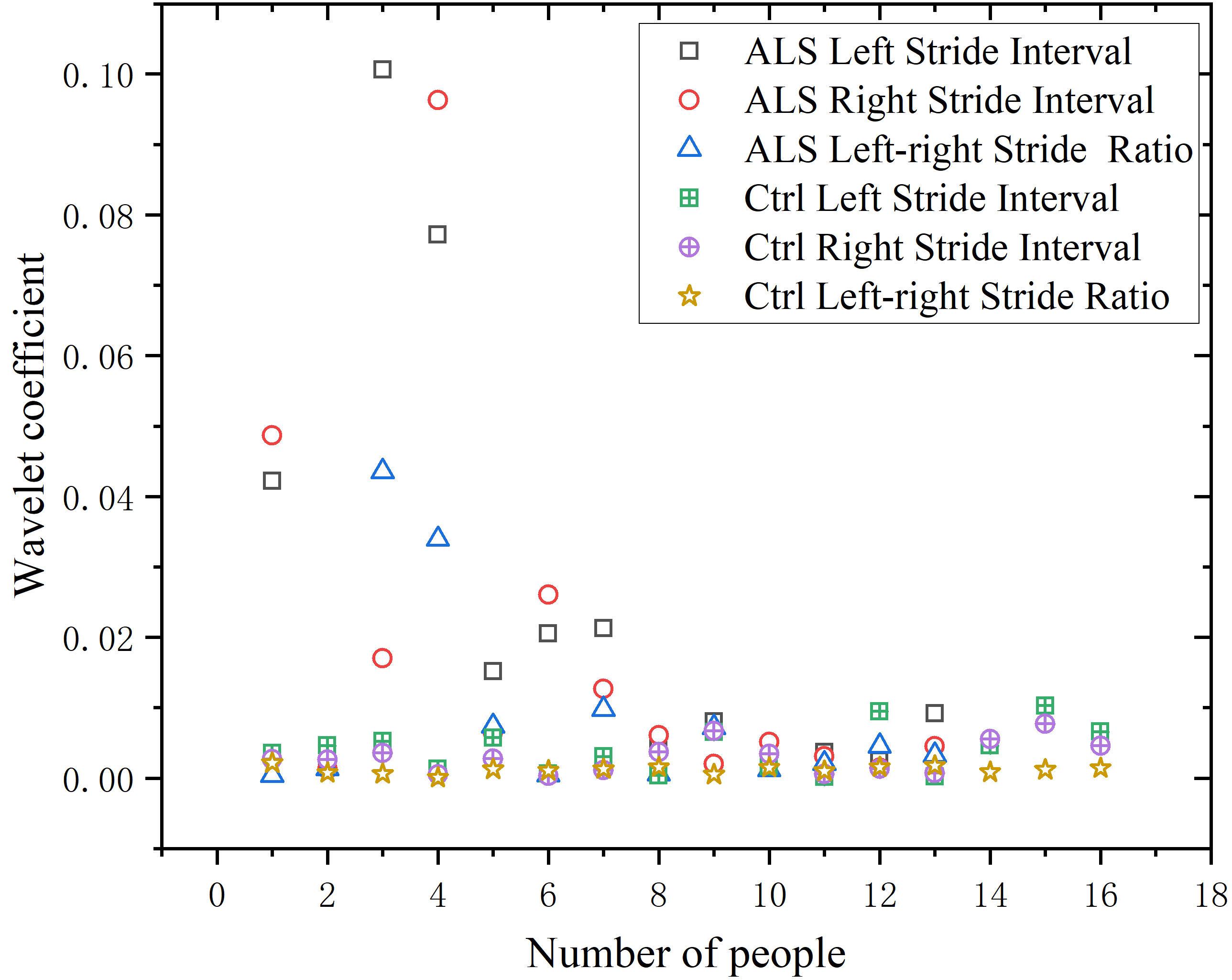

Supplement: Supplementary file 2 [file Data_Sheet_2.zip › Data Sheet 1/8a.png]

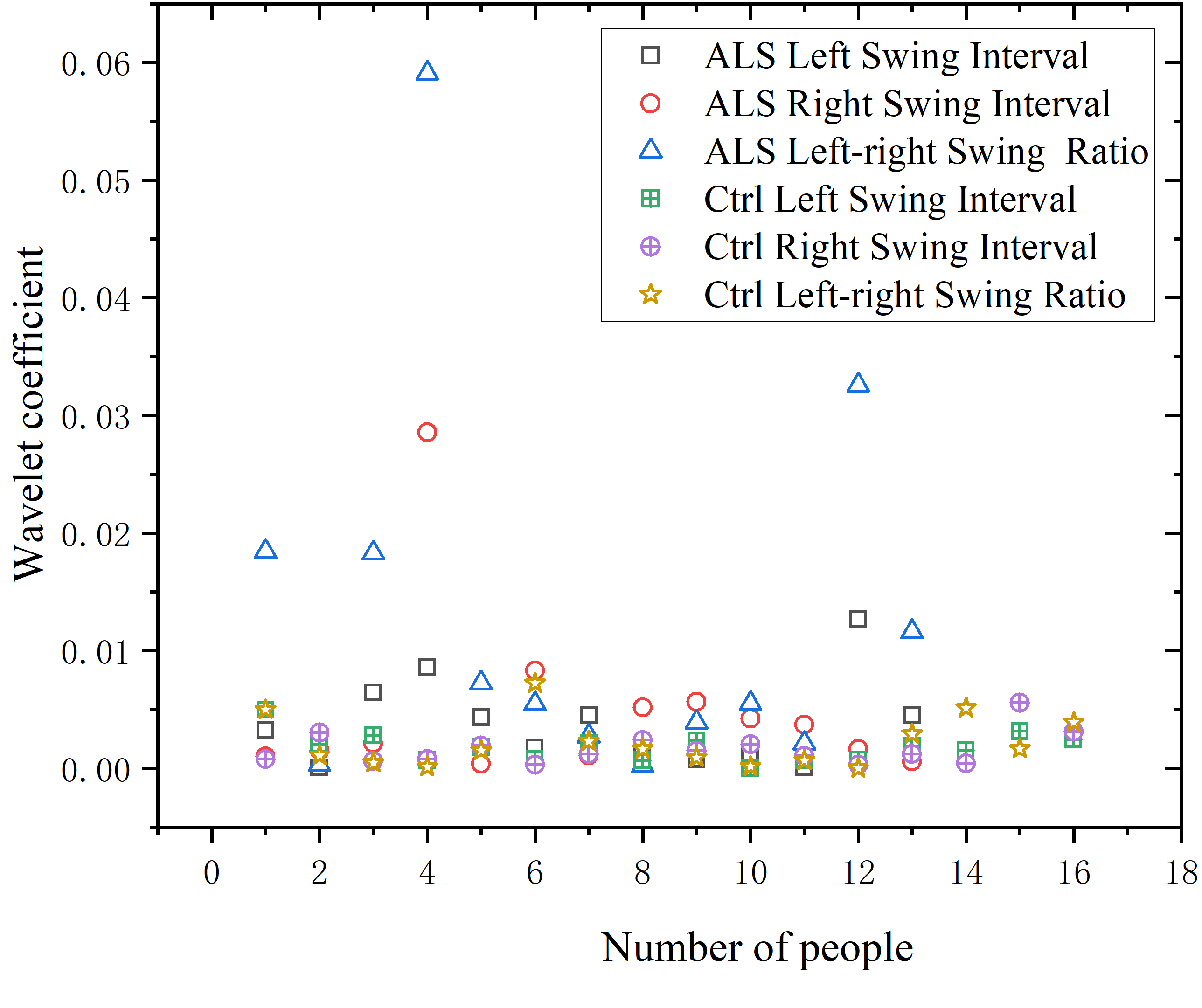

Supplement: Supplementary file 2 [file Data_Sheet_2.zip › Data Sheet 1/8b.png]

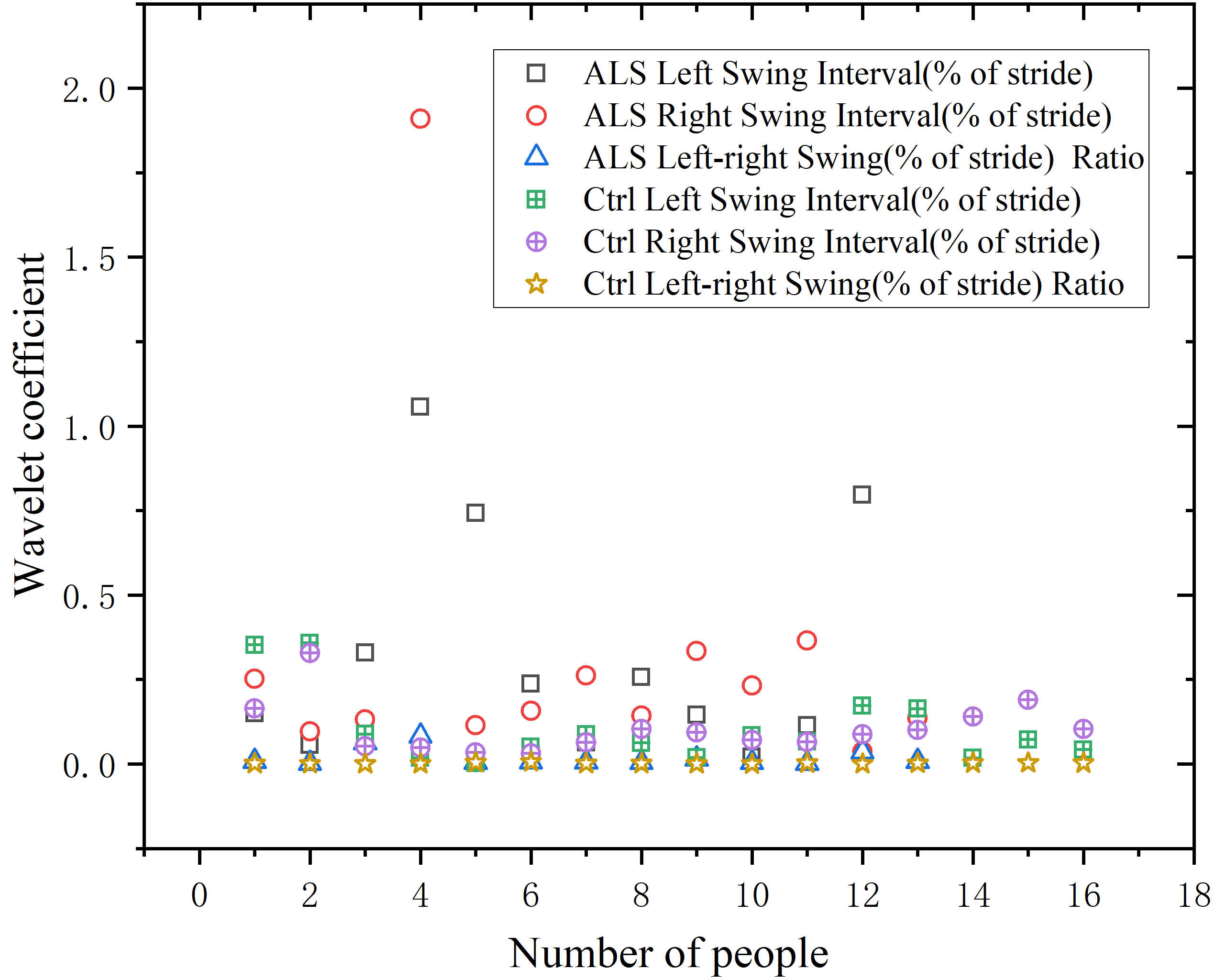

Supplement: Supplementary file 2 [file Data_Sheet_2.zip › Data Sheet 1/8c.png]

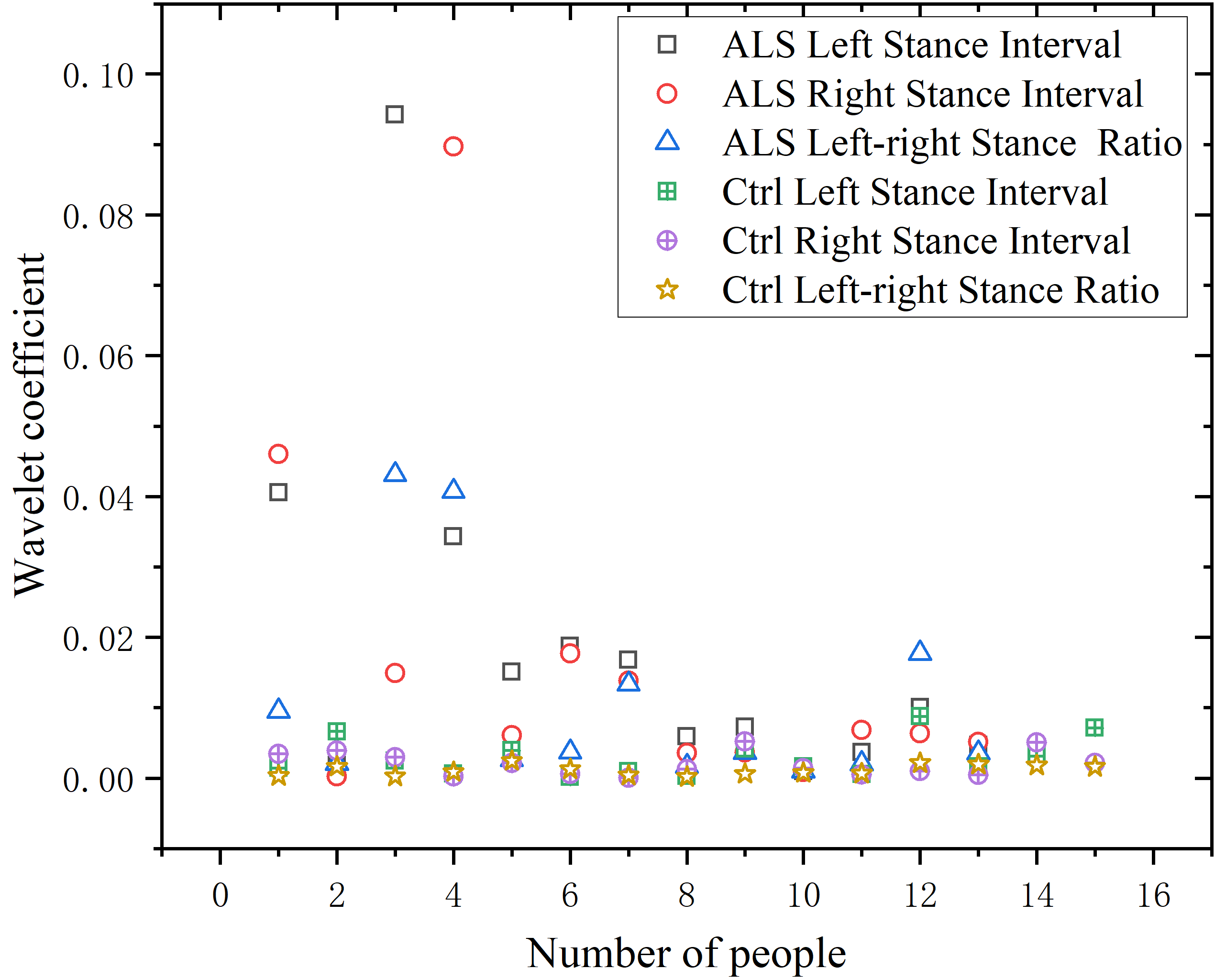

Supplement: Supplementary file 2 [file Data_Sheet_2.zip › Data Sheet 1/8d.png]

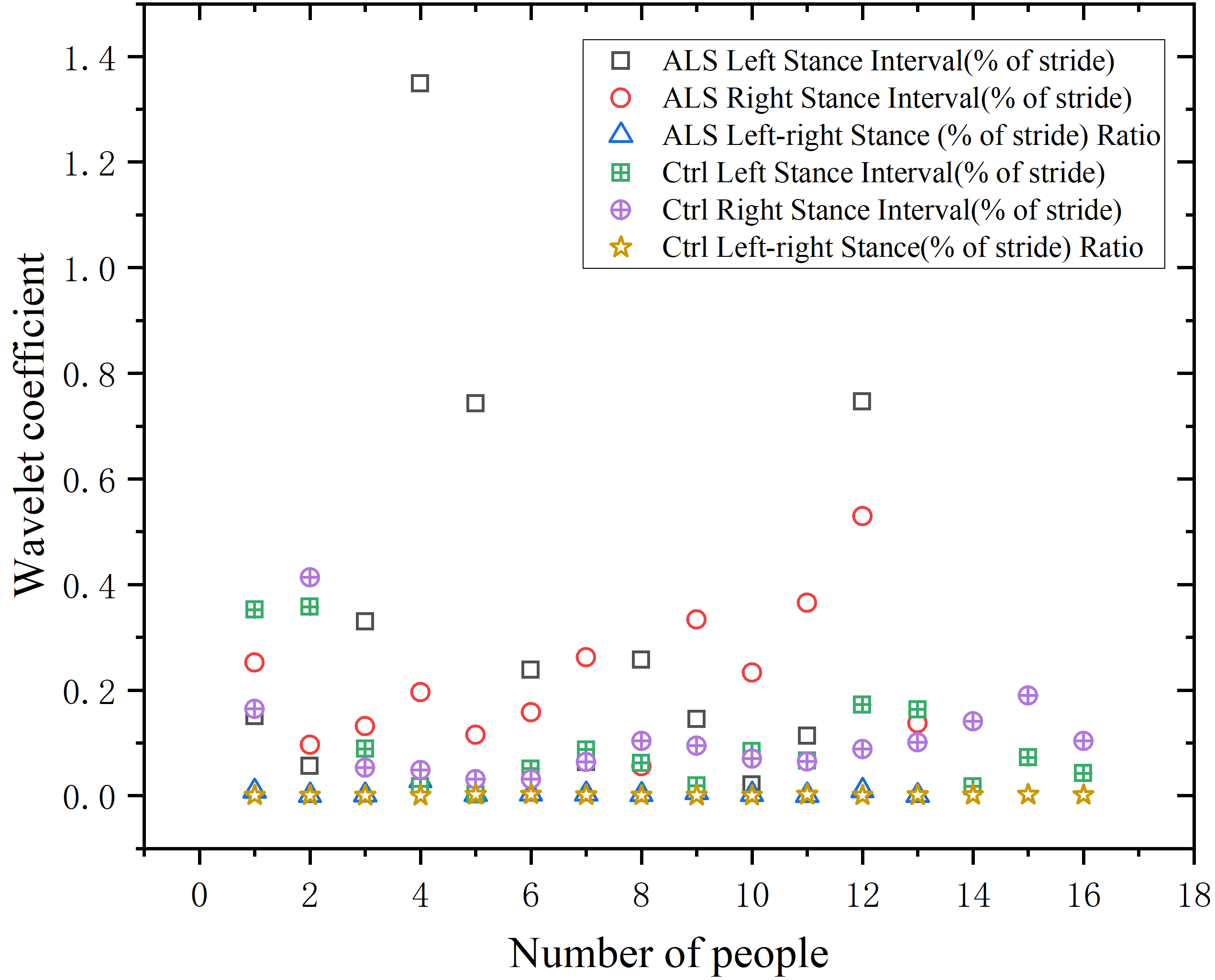

Supplement: Supplementary file 2 [file Data_Sheet_2.zip › Data Sheet 1/8e.png]

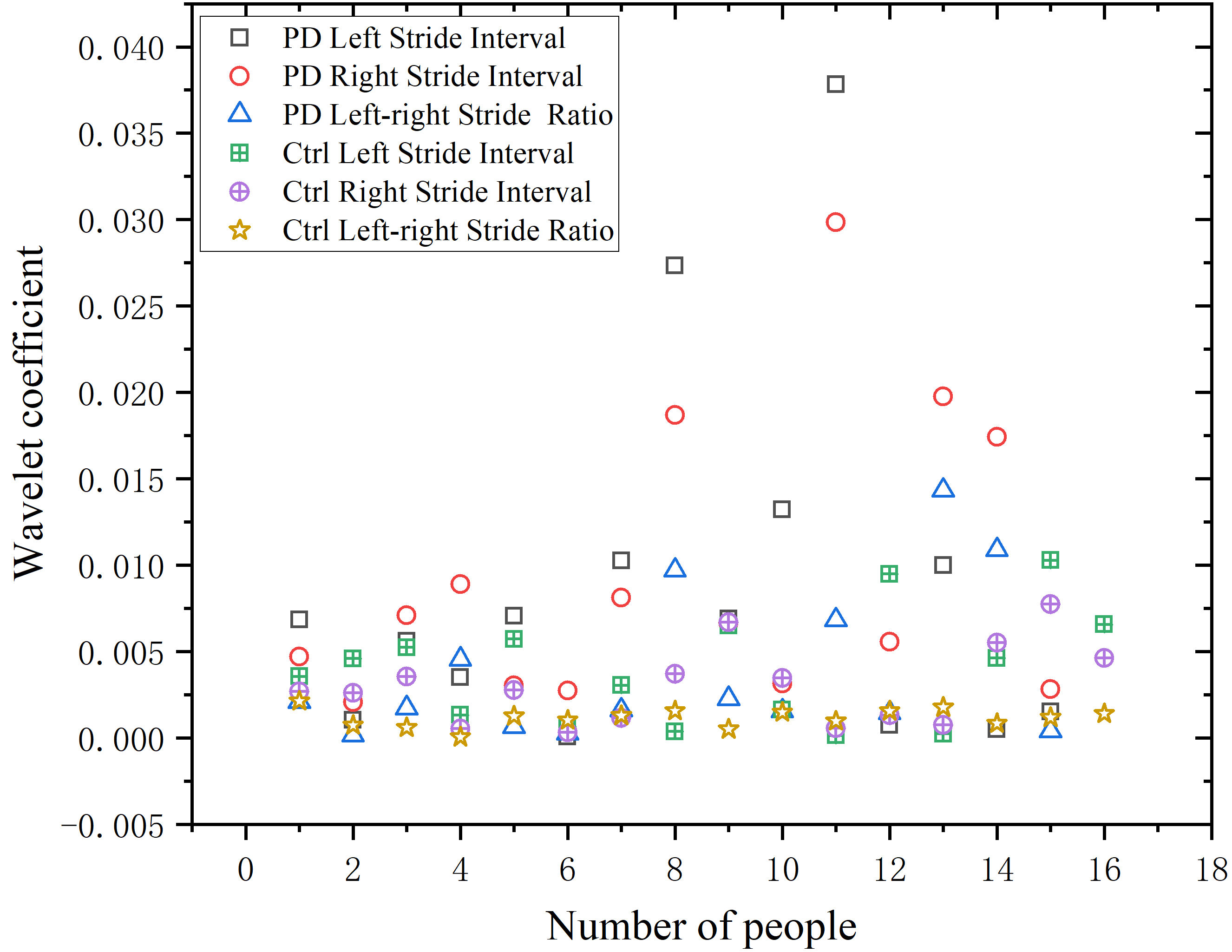

Supplement: Supplementary file 2 [file Data_Sheet_2.zip › Data Sheet 1/9a.png]

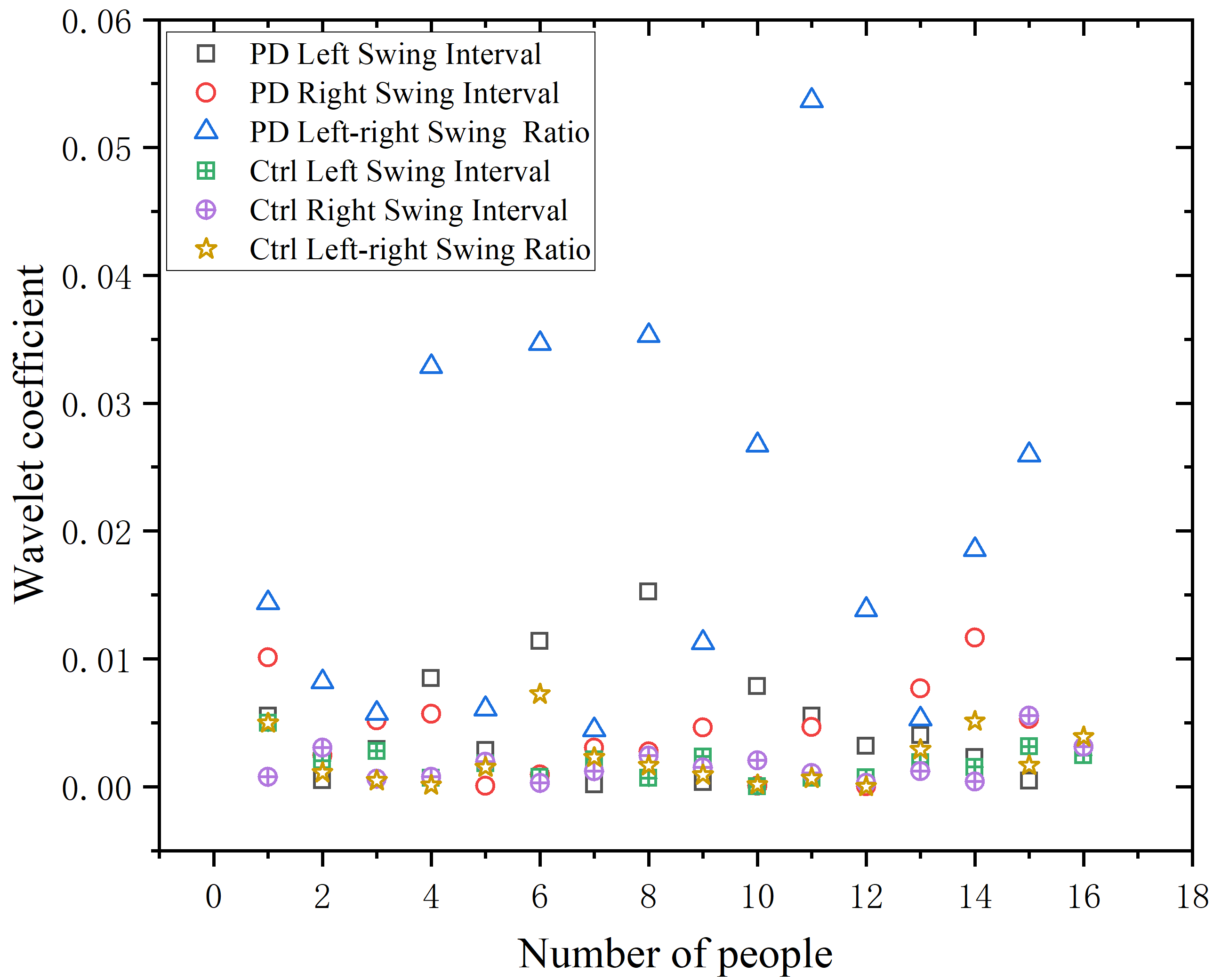

Supplement: Supplementary file 2 [file Data_Sheet_2.zip › Data Sheet 1/9b.png]

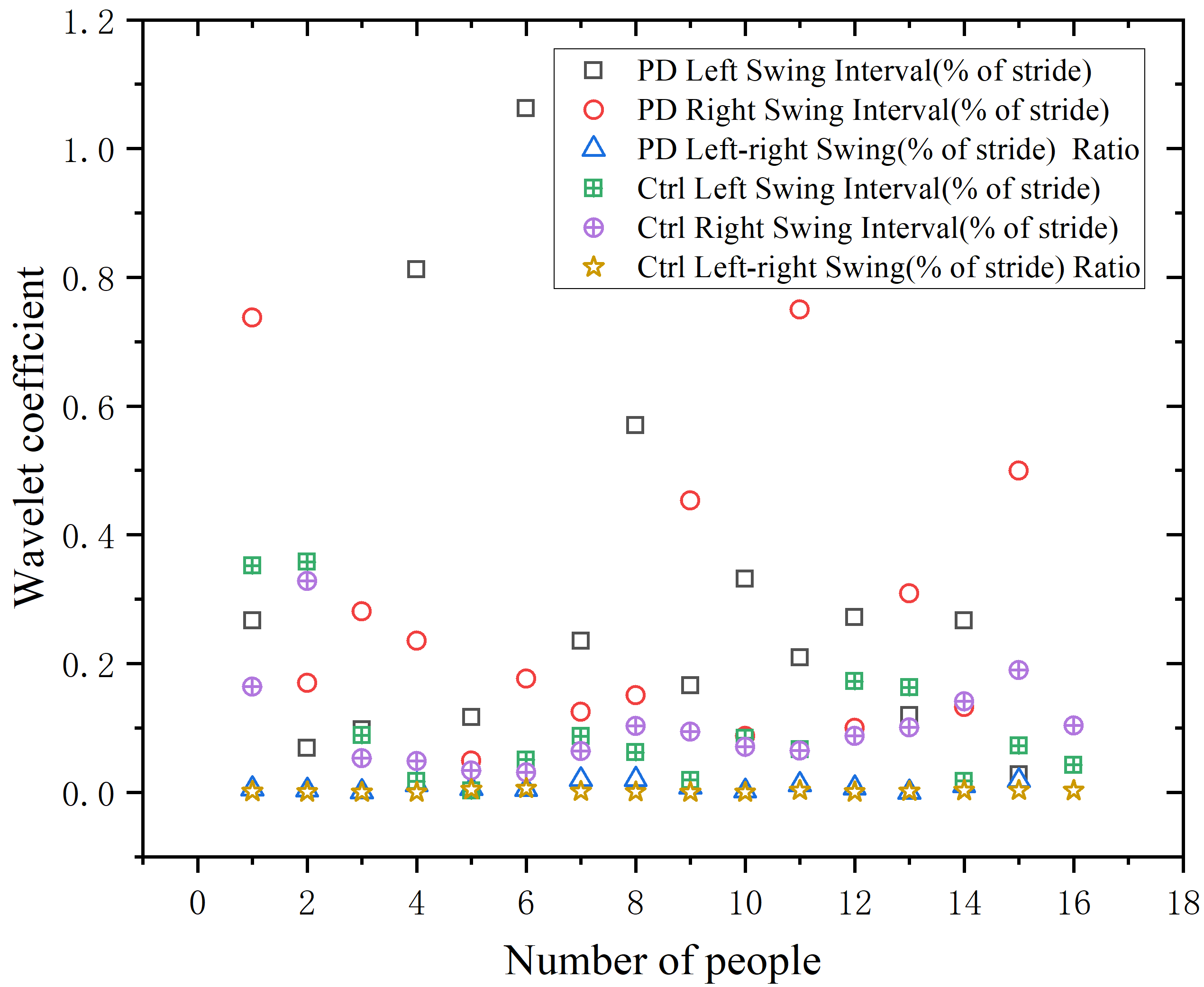

Supplement: Supplementary file 2 [file Data_Sheet_2.zip › Data Sheet 1/9c.png]

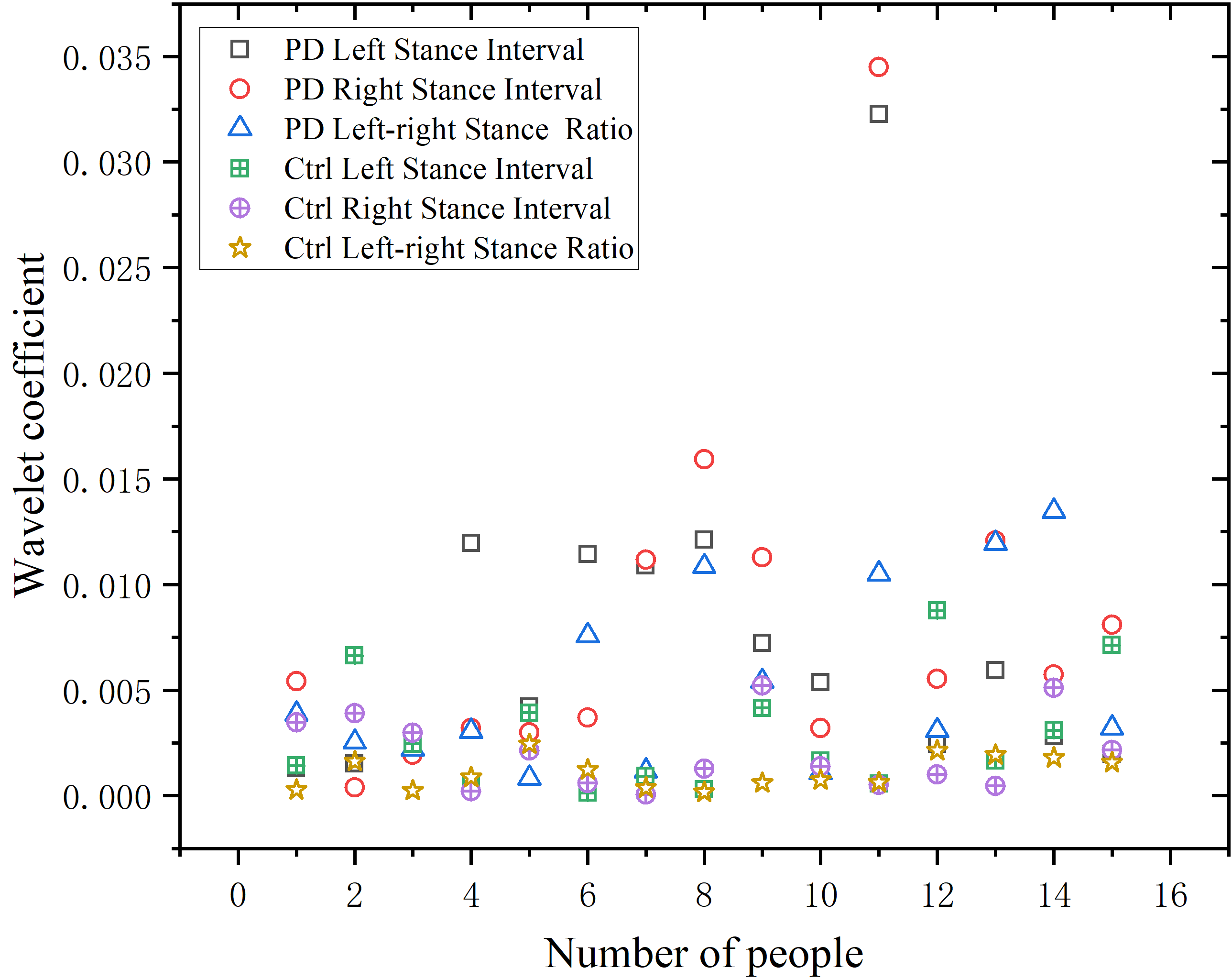

Supplement: Supplementary file 2 [file Data_Sheet_2.zip › Data Sheet 1/9d.png]

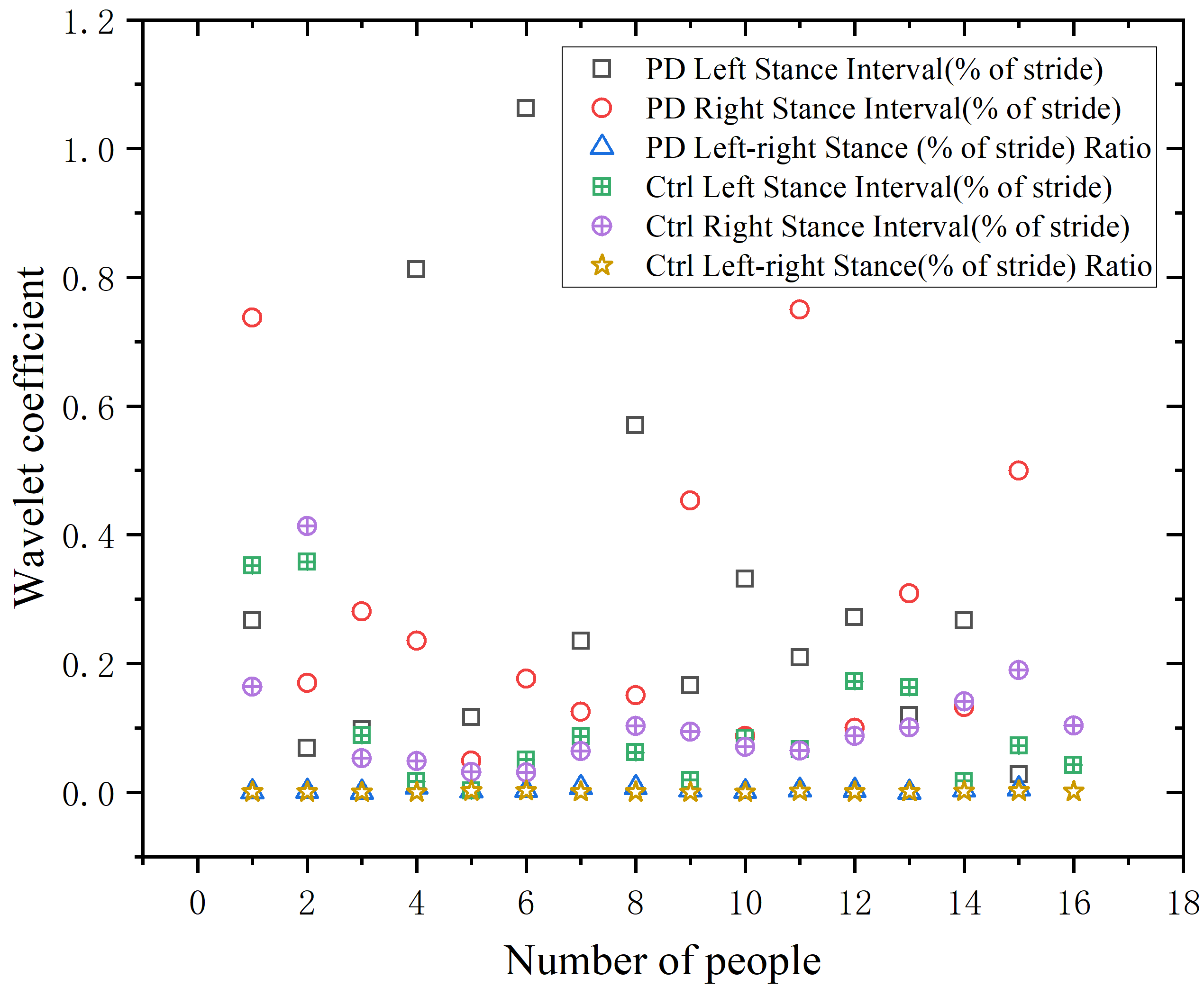

Supplement: Supplementary file 2 [file Data_Sheet_2.zip › Data Sheet 1/9e.png]
